# Evaluating progesterone receptor agonist megestrol plus letrozole for women with early-stage estrogen-receptor-positive breast cancer: the window-of-opportunity, randomized, phase 2b, PIONEER trial

**DOI:** 10.1038/s43018-025-01087-x

**Published:** 2026-01-05

**Authors:** Rebecca A. Burrell, Sanjeev Kumar, Elena Provenzano, Cleopatra Pike, Alimu Dayimu, Stuart A. McIntosh, Vassilis Pitsinis, Polly King, Beatrix Elsberger, Sasi Govindarajulu, Lucy Satherley, Sirwan Hadad, Peter Schmid, Amit Agrawal, Bodiere Akpuluma, Steven Bell, John R. Benson, Carlos Caldas, Danya Cheeseman, Igor Chernukhin, Parto Forouhi, Tulay Gulsen, Eleftheria Kleidi, Karen Pinilla, Wendi Qian, Jean E. Abraham, Jason S. Carroll, Richard D. Baird

**Affiliations:** 1 Cancer Research UK Cambridge Centre, Cambridge, UK; 2 NIHR Cambridge Biomedical Research Centre, Cambridge, UK; 3 Department of Oncology, University of Cambridge, Cambridge, UK; 4 Cambridge Clinical Trials Unit Cancer Theme, Cambridge, UK; 5 Patrick G Johnston Centre for Cancer Research, Queen’s University Belfast, Belfast, UK; 6 Department of Breast Surgery, Ninewells Hospital and Medical School, Dundee, UK; 7 Royal Cornwall Hospitals NHS Trust, Truro, UK; 8 Aberdeen Royal Infirmary/University of Aberdeen, Breast Unit, Aberdeen, UK; 9 Southmead Hospital, North Bristol NHS Trust, Bristol, UK; 10 Cardiff University-Peking University Cancer Institute, Cardiff University School of Medicine, Cardiff, UK; 11 Women’s Health, University College London Hospitals NHS Foundation Trust, London, UK; 12 Barts Cancer Institute, Queen Mary University of London, London, UK; 13 Cambridge Breast Unit, Addenbrooke’s Hospital, Cambridge, UK; 14 Cancer Research UK Cambridge Institute, Cambridge, UK; 15 Present Address: Chris O’Brien Lifehouse, Camperdown, New South Wales Australia

**Keywords:** Cancer, Cancer

## Abstract

The use of progestogens in breast cancer has been controversial. Recent preclinical studies have shown that ligand-bound progesterone receptor interacts directly with the estrogen receptor (ER) and reprograms ER transcriptional activity. Progestogen cotreatment enhances the antitumor activity of antiestrogen therapy in mouse xenografts. We report PIONEER, a 198-participant, three-arm, randomized phase 2b window-of-opportunity study for women with early-stage ER^+^ breast cancer, which evaluated letrozole with or without megestrol at 40 mg or 160 mg daily. The primary endpoint was the change in tumor proliferation measured by Ki67 immunohistochemistry. Secondary and exploratory endpoints included a comparison of low versus higher dose of megestrol, safety, tolerability and biomarker subgroup analyses. The trial met its primary endpoint, with a greater reduction in proliferation seen when megestrol was added to letrozole. This effect was accompanied by reduced ER genomic binding at canonical binding sites in paired tumor biopsies, indicating reduced ER transcriptional activity. These results support further evaluation of low-dose megestrol, which has two mechanisms for potentially improving breast cancer outcomes in combination with standard antiestrogen therapy: alleviating hot flashes and thereby helping with treatment adherence, as well as a direct antiproliferative effect (NCT03306472).

## Abstract (web summary)

Baird et al. present the phase 2 PIONEER trial findings on the antitumor activity of combining aromatase inhibitor letrozole with megestrol in postmenopausal women with operable estrogen-receptor-positive human epidermal-growth-factor-receptor-2-negative breast cancer.

## Main

Approximately three quarters of breast cancers express the transcription factor estrogen receptor-α (hereafter ER). Inhibition of ER activity is the backbone of therapy for early-stage and advanced-stage ER^+^ breast cancer. However, these treatments fail for many persons and side effects mean that many prematurely stop adjuvant therapy, adversely impacting clinical outcomes^1,2^.

Clinical trial data^3–9^ support the use of progestogens (compounds that activate the progesterone receptor (PR)) to treat some persons with ER^+^ breast cancer. Megestrol acetate (megestrol, also known as Megace) is licensed for treatment of metastatic ER^+^ breast cancer at the higher dose of 160 mg daily. Hot flashes are frequent among women taking antiestrogen therapy^10^. Low doses of megestrol (20–40 mg daily) can alleviate these symptoms in 75–85% of women^10,11^, potentially improving cancer treatment adherence, but are not currently licensed for this indication. With long-term use, the side-effect profile of high megestrol doses (160–800 mg daily) can include weight gain, hypertension and increased risk of venous thromboembolism (VTE), whereas lower doses have a more favorable profile^8,10–12^.

Some clinicians have hesitated to use progestogens for breast cancer therapy or for treatment of intolerable hot flashes because of the controversial results of some menopausal hormone therapy (MHT) trials^9,13–16^. In particular, initial data from the Women’s Health Initiative (WHI) suggested that MHT containing the progestogen medroxyprogesterone acetate caused an increased risk of breast cancer^17^. Prolonged follow-up (18 years) from the WHI trial concluded that overall mortality was not affected by the inclusion of a progestin^18^, in contrast to the initial conclusions that drove a decrease in MHT use worldwide^19^. In other studies, MHT containing progesterone or dydrogesterone was not associated with increased breast cancer risk^20,21^ and high circulating levels of endogenous progesterone have been correlated with a reduced risk of breast cancer in premenopausal women^22^. As such, different progestogens have distinct pharmacology and should not be treated as one class of therapeutics^23^.

More recently, laboratory studies revealed that treating ER^+^ breast cancer cells with progesterone induces an ER–PR interaction, dramatically altering ER transcriptional activity and decreasing tumor cell proliferation^24,25^. Treating mouse xenograft models with both progesterone and antiestrogen therapy led to greater inhibition of tumor growth than either treatment alone^24^. This improved antitumor activity probably reflects a direct inhibition of ER activity plus sequestration of ER away from canonical target genes to different genomic loci through an induced interaction with PR.

To assess this potential therapeutic strategy, we designed the PIONEER trial, (preoperative-window study of letrozole plus PR agonist megestrol acetate versus letrozole alone in postmenopausal women with ER^+^ breast cancer) to evaluate whether combining the aromatase inhibitor (AI) letrozole with megestrol improves antitumor activity in postmenopausal women with operable ER^+^ human epidermal growth factor receptor 2 (HER2, also known as ERBB2)-negative breast cancer. PIONEER compared two megestrol doses: the higher dose of 160 mg (known to have therapeutic activity in persons with ER^+^ breast cancer) and the lower dose of 40 mg, which can alleviate hot flashes associated with antiestrogen therapy. The 40-mg dose has a favorable side-effect profile relative to 160 mg but has not previously been assessed for tumor antiproliferative activity.

## Results

### Recruitment and baseline characteristics

**Fig. 1:**
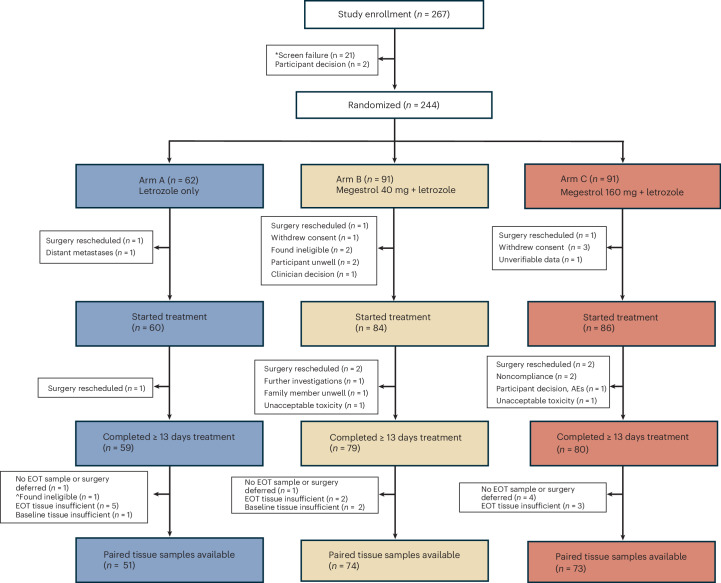
Consort diagram. *A total of 13 participants failed screening because of a planned surgery date that was incompatible with the trial treatment window, many of which occurred during the COVID19 pandemic. ^One participant was belatedly reported to be HER2 positive.

Between July 2017 and October 2022, 244 women with early-stage ER^+^ breast cancer from ten UK hospitals were randomized 2:3:3 to receive preoperative treatment in one of three arms: arm A, letrozole alone; arm B, letrozole + lower-dose megestrol (40 mg); arm C, letrozole + higher-dose megestrol (160 mg) (Fig. 1). The primary objective was to assess the change in tumor proliferation (measured by Ki67) between baseline and end of treatment (EOT) in the combination arms (B and C) compared to the control arm (A). Further details of the study design are provided in the Methods. A total of 230 participants took at least one dose of treatment and constituted the safety analysis population. A total of 218 participants completed a minimum of 13 days of treatment, of which 201 participants had an adequate EOT sample for Ki67. Reasons for withdrawal before and during trial treatment are summarized in Fig. 1. The primary analysis included 198 evaluable participants who completed at least 13 days of treatment and had tumor samples with sufficient cellularity for Ki67 assessment at both baseline and EOT (arm A, *n* = 51; arm B, *n* = 74; arm C, *n* = 73).

Baseline participant and tumor characteristics were similar across treatment arms (Table 1). There were more participants with grade 3 tumors in the control arm but mean Ki67 values were well balanced at baseline. A total of 194/198 (98%) tumors had an ER Allred score of 7–8 and 11% (*n* = 22/198) were defined as PR^−^ (<1% positive cells^26^), with 22% having a PR Allred score of 0–3 (an alternative definition of PR negativity^27^).

**Table 1:** Baseline participant characteristics for the evaluable population (*n* = 198)

	Arm A (*n* = 51)	Arm B (*n* = 74)	Arm C (*n* = 73)	Arms B + C (*n* = 147)
Age, median (IQR)	67.2 (10.3)	67.9 (12.1)	68.4 (11.0)	68.1 (11.9)
**ECOG performance status**				
0	43 (84%)	66 (89%)	60 (82%)	126 (86%)
1	7 (14%)	7 (9%)	12 (16%)	19 (13%)
2	1 (2%)	1 (1%)	1 (1%)	2 (1%)
**Histological grade**				
1	5 (10%)	9 (12%)	4 (5%)	13 (9%)
2	32 (63%)	52 (70%)	56 (77%)	108 (73%)
3	14 (27%)	13 (18%)	13 (18%)	26 (18%)
**Histological subtype**				
Ductal	38 (75%)	60 (81%)	51 (70%)	111 (76%)
Lobular	10 (20%)	9 (12%)	14 (19%)	23 (16%)
Other	3 (6%)	5 (7%)	8 (11%)	13 (9%)
**ER Allred score**				
3–6	1 (2%)	1 (1%)	2 (3%)	3 (2%)
7–8	50 (98%)	73 (99%)	71 (97%)	144 (98%)
**T stage**				
1C	24 (47%)	48 (65%)	46 (63%)	94 (64%)
2	24 (47%)	22 (30%)	26 (36%)	48 (33%)
3	3 (6%)	4 (5%)	1 (1%)	5 (3%)
**N stage**				
0	45 (88%)	61 (82%)	65 (89%)	126 (86%)
1	4 (8%)	11 (15%)	5 (7%)	16 (11%)
2	1 (2%)	1 (1%)	1 (1%)	2 (1%)
3	-	1 (1%)	1 (1%)	2 (1%)
X	1 (2%)	-	1 (1%)	1 (1%)
PR Allred score*				
0–3	10 (20%)	14 (19%)	20 (28%)	34 (23%)
4–6	11 (22%)	12 (16%)	12 (17%)	24 (17%)
7–8	30 (59%)	47 (64%)	40 (56%)	87 (60%)
**PR status**				
Negative	7 (14%)	5 (7%)	10 (14%)	15 (10%)
Positive (>1%)	44 (86%)	69 (93%)	63 (86%)	132 (90%)
**AR status**^†^				
Negative	1 (2%)		2 (3%)	2 (1%)
Positive (>1%)	48 (98%)	73 (100%)	71 (97%)	144 (99%)
**Ki67 status**				
<10%	10 (20%)	5 (7%)	19 (26%)	24 (16%)
>10%	41 (80%)	69 (93%)	54 (74%)	123 (84%)
Mean baseline Ki67^‡^	18.9 (2.0)	20.2 (1.6)	18.1 (2.0)	19.1 (1.8)

*Two participants, one in arm B and one in arm C, had missing PR Allred scores. ^†^Three participants, two in arm A and one in arm B, had missing AR status. ^‡^The geometric mean and s.d. are reported. IQR, interquartile range.

### Tumor proliferation

**Fig. 2:**
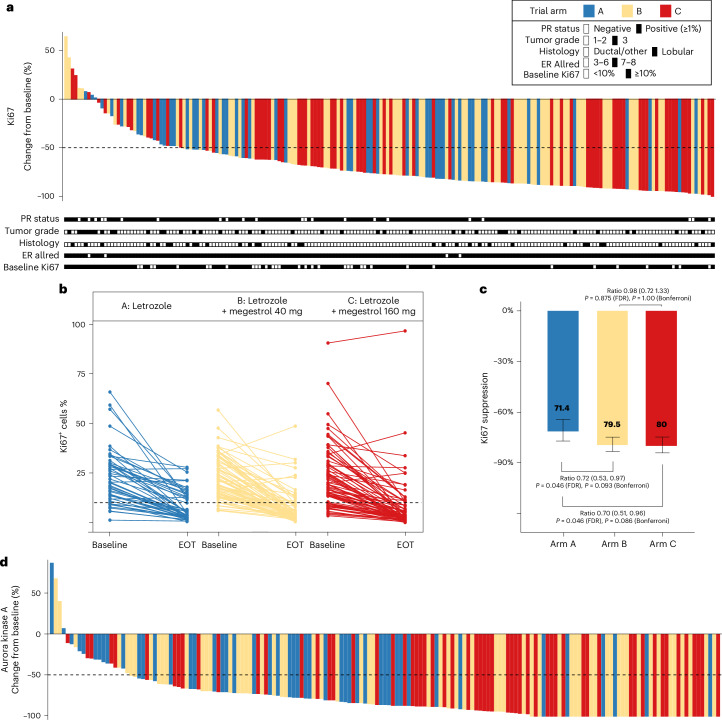
Antiproliferative response to treatment in all evaluable participants. **a**, Percentage change in Ki67 for each participant, sorted from low to high. PR status, tumor grade, histological subtype, ER Allred score and baseline Ki67 are indicated in black and white boxes under each participant (*n* = 198). **b**, Individual changes in percentage Ki67 positivity from baseline to EOT. **c**, Pairwise comparisons of Ki67 suppression for participants in arms A (*n* = 51), B (*n* = 74) and C (*n* = 72), defined as the geometric mean of Ki67 proportional change (EOT/baseline − 1). Error bars represent the 95% CI. Statistical analysis was conducted using two-sided *t*-tests of the geometric means with either false discovery rate (FDR) or Bonferroni correction for multiple testing. **d**, Percentage change from baseline in AURKA positivity, sorted from low to high (*n* = 169 participants). Source data

**Extended Data Fig. 1:**
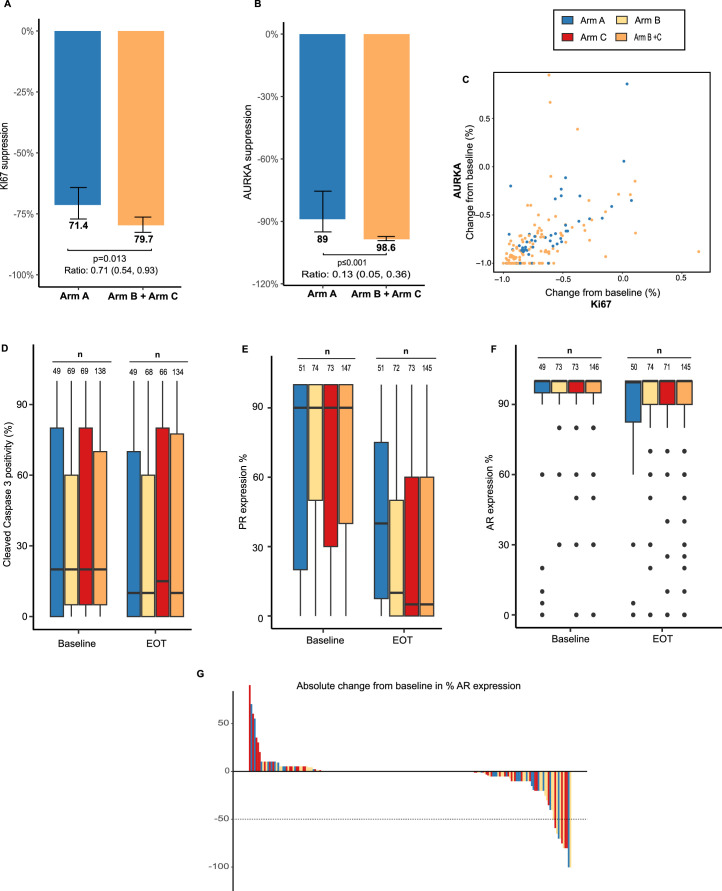
Anti-proliferative response to treatment. **A**) Ki67 suppression in Arms A vs B + C combined, defined as geometric mean of Ki67 proportional change (EOT/baseline)-1. Arms A (n = 51 patients), B (n = 74 patients), and C (n = 72 patients). **B**) AURKA suppression in Arms A vs B + C, defined as for Ki67. Arm A (n = 48 patients), Arm B (n = 121 patients). Statistical comparisons in panels A + B are two-sided t-tests of the geometric means of Ki67 proportional change. Error bars are 95% Confidence Intervals (CI). **C**) Visualised correlation between change in Ki67 and change in AURKA from baseline. Data points are coloured according to treatment allocation. Spearman’s rank correlation *r* = 0.64, p = 5.34e-21. **D**–**F**) Boxplots of **D**) cleaved caspase 3, **E**) PR expression and **F**) AR expression at baseline and end of treatment (EOT), by trial arm. Box = IQR, centre = median, whiskers = min/max (excluding outliers, defined as <Q1 – 1.5*IQR or > Q3 + 1.5*IQR). n = number of patients. **G**) Individual absolute change in AR expression from baseline to end of treatment (EOT) (n = 189 patients). Source data

The trial primary endpoint showed a significantly greater reduction in Ki67 with megestrol combinations (arms B + C) versus letrozole alone (A) (ratio of geometric mean (GMR) proportional change in Ki67: 0.71, 95% confidence interval (CI): 0.54–0.93, *P* = 0.013) (Table 2, Fig. 2a,b and Extended Data Fig. 1a). The mean Ki67 suppression for each arm was as follows: arm A (letrozole), 71.4% (95% CI: 64–77%); arm B (letrozole + 40 mg of megestrol), 79.5% (95% CI: 75–83%); arm C (letrozole + 160 mg of megestrol), 80% (95% CI: 75–84%) (Fig. 2c). This result remained significant after adjustment for tumor grade (GMR proportional change: 0.74, 95% CI: 0.57–0.96, *P* = 0.024) (Table 2).

**Table 2:** Antiproliferative response to treatment measured by Ki67 and AURKA

	Arm A	Arms B + C	Ratio (95% CI)	*P*
Ki67 EOT/baseline	0.29 (0.23–0.36) *n* = 51	0.20 (0.17–0.24) *n* = 146^^^	0.71 (0.54–0.93)	0.013
Ki67 EOT/baseline adjusted for tumor grade*	0.30 (0.24–0.38) *n* = 51	0.22 (0.18–0.27) *n* = 146	0.74 (0.57–0.96)	0.024
Ki67 EOT	5.42 (4.10–7.15) *n* = 52	3.86 (3.23–4.62) *n* = 148	0.71 (0.51–0.99)	0.043
AURKA EOT/baseline	0.11 (0.05–0.25) *n* = 48	0.01 (0.01–0.03) *n* = 121	0.13 (0.05–0.36)	<0.001
EOT Ki67 < 10%^†^	64.7 (50.1–77.6) *n* = 52	79.6 (72.2–85.8) *n* = 149	0.81 (0.64–0.99)	0.033
EOT Ki67 ≤ 2.7%^†^	26.9 (15.6–41.0) *n* = 52	39.6 (31.7–47.9) *n* = 149	0.68 (0.35–1.04)	0.102

Ki67 and AURKA EOT/baseline are the geometric means of proportional change (EOT/baseline). *P* values are based on a *t*-test of the geometric means. 95% CIs are reported for GMRs. Ki67 EOT is the geometric mean of EOT Ki67 values, presented on the original scale. ^One participant in arm C had an EOT Ki67 of 0 and was excluded from analyses requiring log transformation of Ki67 proportional change. *Analysis was adjusted for diagnostic tumor grade. ^**†**^The proportion of participants and 95% CI using the Clopper–Pearson method. The ratio of the proportions and 95% CI based on 1,000 bootstraps are reported for comparison.

There was no difference in Ki67 suppression between arms B and C (GMR: 0.98, 95%: CI 0.72–1.33) (Fig. 2c). These results suggest that both lower (40 mg) and higher (160 mg) doses of megestrol were similarly effective at further reducing breast cancer cell proliferation when combined with letrozole.

Tumor proliferation was also assessed by aurora kinase A (AURKA) immunohistochemistry (IHC)^28^. Consistent with the Ki67 results, there was a significantly greater reduction in AURKA positivity in the megestrol combination arms compared to letrozole only (GMR: 0.13, 95% CI: 0.05–0.36, *P* < 0.001) (Table 2, Fig. 2d and Extended Data Fig. 1b). Reductions in Ki67 and AURKA were highly correlated (Spearman’s rank correlation *r* = 0.64, *P* = 5.34 × 10^−21^) (Extended Data Fig. 1c), confirming the use of AURKA as an alternative measure of proliferation^28^.

The mean EOT Ki67 levels were 5.4% after letrozole alone (A) versus 3.9% after megestrol combination treatment (B + C) (GMR: 0.71, 95% CI: 0.51–0.99, *P* = 0.043) (Table 2). Exploratory analysis of previously published Ki67 values to define response to treatment^29,30^ revealed that the proportion of participants with an EOT Ki67 value of ≤10% was 64.7% in arm A versus 79.6% in arms B + C (risk ratio: 0.81, 95% CI: 0.64–0.99, *P* = 0.033) (Table 2). Complete cell-cycle arrest (EOT Ki67 ≤ 2.7%), a previously established marker of excellent response to antiestrogen monotherapy and combination therapies^29–32^, was also more frequent after megestrol combination treatment (arm A, 26.9%; arms B + C, 39.6%; *P* = 0.102). Cleaved caspase 3 staining revealed an overall slight reduction in positivity at EOT compared to baseline and no significant difference between control and megestrol combination arms (Extended Data Fig. 1d), in keeping with the predominantly cytostatic action of endocrine therapies.

### Adverse events (AEs)

The short treatment duration in window-of-opportunity studies allows for only a limited assessment of treatment safety and tolerability. Among participants taking at least one dose of trial treatment (*n* = 230), there were similar rates of AEs reported across arms A, B and C (arm A, 58.3%; arm B, 60.7%; arm C, 66.3%), with the majority being grade 1 (Supplementary Table 1). Grade ≥3 AEs were infrequent (arm A, 3.3%; arm B, 2.4%; arm C, 4.7%). A higher proportion of participants reported grade 2 AEs following megestrol combination therapy (arm A, 11.7%; arm B, 21.4%; arm C, 23.3%).

The most common AEs were arthralgia, fatigue, headache, nausea and hot flashes. AEs observed specifically after megestrol combination therapy included dry mouth (arm B, 10%; arm C, 5%), dyspnea (arm B, 2%; arm C, 6%) and vaginal bleeding (arm B, 4%; arm C, 3%). A total of five participants (5.81%, *n* = 5/86) in arm C (higher-dose megestrol) had grade 2/3 hypertension reported during the trial. Treatment-emergent hypertension was not observed in arms A or B (Supplementary Tables 2 and 3). Figure 1 and Supplementary Table 4 provide further detail on AEs.

There were three serious AEs: a grade 3 postoperative hematoma requiring surgical intervention (arm A) and two episodes of VTE (*n* = 1 each in arms B and C) (Supplementary Table 3). Both episodes of VTE were considered possibly related to trial treatment and probably related to breast cancer surgery as they occurred 1–2 weeks postoperatively in participants with a body mass index of ≥33. Additionally, one participant was found to have metastatic disease during investigations for VTE. We did not observe VTE at higher-than-expected frequencies after breast cancer surgery during the trial^33^.

### Nuclear receptor expression and activity

**Fig. 3:**
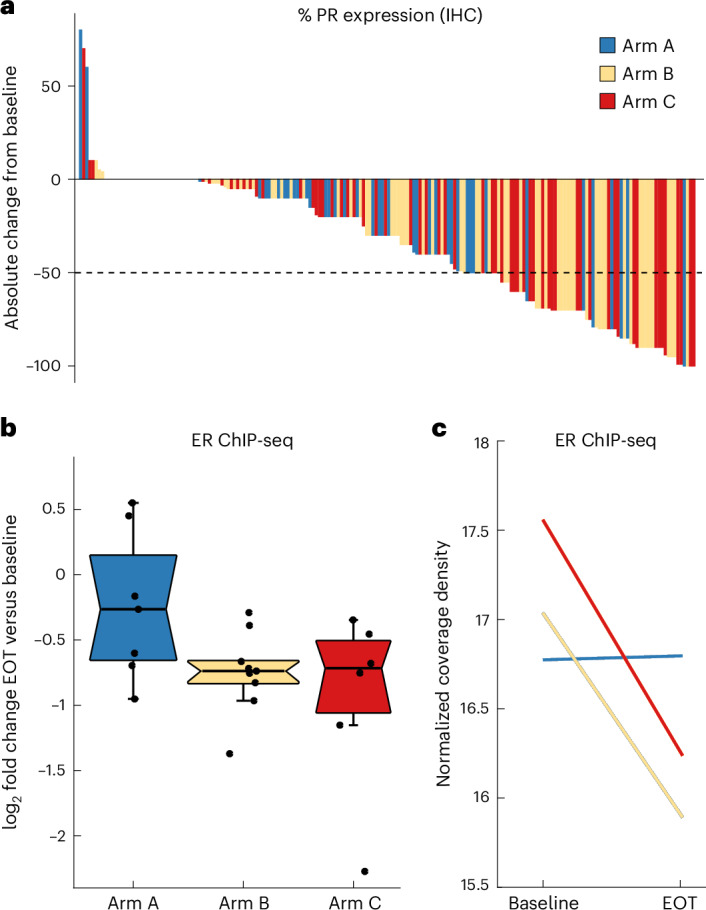
ER transcriptional activity by IHC and ChIP-seq. **a**, Individual absolute change in percentage PR expression from baseline to EOT (*n* = 174 participants). **b**, Box plot of log-transformed fold change in binding intensity across core ER sites (as defined in a previous study^37^) for paired baseline and EOT samples, grouped by trial arm (*n* = 22 sample pairs). Box, IQR; center, median; whiskers, min–max (excluding outliers, defined as <Q1 − 1.5 × IQR or >Q3 + 1.5 × IQR). **c**, Least squares lines summarizing the data distribution within each sample group, showing the change in ER binding from baseline to EOT. Source data

PR expression (percentage of PR^+^ cells by IHC) was reduced following treatment in all arms, consistent with the ER dependence of PR gene expression (Fig. 3a). There was significantly greater repression of PR in the megestrol combination arms versus letrozole only (median percentage of PR^+^ cells at EOT: arm A, 40%; arms B + C, 5%; nonparametric *P* = 0.001) (Extended Data Fig. 1e), indicating greater inhibition of ER transcriptional activity, in support of the Ki67 proliferation endpoint. The degree of PR repression was similar between lower-dose and higher-dose megestrol, suggesting that 40 mg of megestrol was sufficient to decrease ER transcriptional activity. A total of 174/196 participants with paired PR IHC were PR^+^ at baseline, of which 25% (*n* = 44/174) had <1% PR^+^ cells at EOT; 91% of these (*n* = 40/44) were in arms B and C. Moreover, 61% of baseline PR^+^ tumors (*n* = 106/174) had ≥10% PR^+^ cells at EOT and 66% of these tumors were in arms B + C. No significant change in AR expression was observed in any treatment arm (Extended Data Fig. 1f,g). Previous reports suggested that AR expression may be predictive of progestin response^34,35^; however, we did not observe a relationship between AR expression level and response.

Preclinical data showed that ligand-bound PR could reprogram ER genomic binding, leading to changes in gene expression and decreased tumor growth in xenograft models^24^. These findings were further explored in tumor samples from the PIONEER trial. ER DNA-binding sites were profiled in paired samples (baseline and EOT) from a subset of participants with fresh-frozen tissue biopsies by chromatin immunoprecipitation with sequencing (ChIP-seq) (Methods). ER peak numbers ranged from 521 to 17,434 within the baseline samples and from 390 to 15,615 in the EOT samples. There was significant variability in the number and location of ER-binding peaks identified in different untreated participant samples at baseline, as previously described^36^. We therefore compared ER binding in paired baseline and EOT samples at genomic loci previously defined as conserved ER-binding events in ER^+^ tumors^37^, which represent the regulatory elements adjacent to canonical ER target genes. Sample pairs with no detectable binding at these loci at baseline were excluded from the analysis. Clinical characteristics of this cohort (*n* = 22) are summarized in Supplementary Table 5.

**Extended Data Fig. 2:**
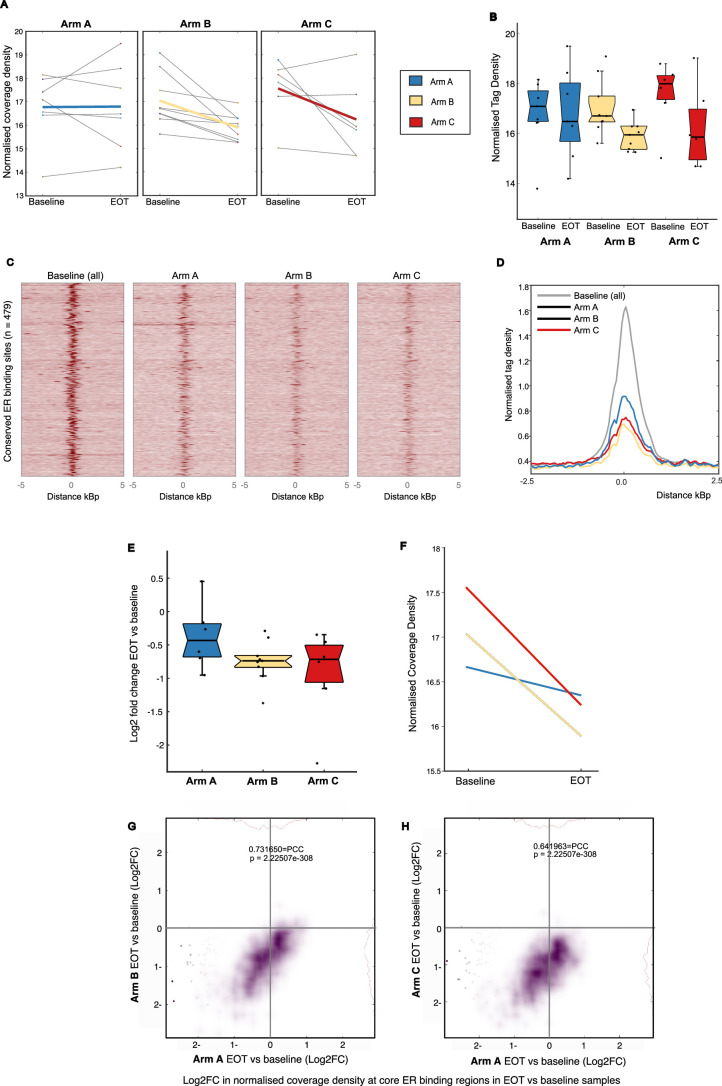
ER Transcriptional Activity by ChIPseq. **A**) Normalised coverage density across core ER sites (as defined in Ross-Innes et al. 2012) in baseline and EOT samples. Paired results for individual patients are joined by dashed black lines. Solid lines are the least squares line summarising the data distribution within each trial arm, as shown in Fig. 3c. **B**) Box plot showing distribution of median normalised coverage density at core ER sites per patient grouped by trial arm, at baseline and EOT. Box = IQR, centre = median, whiskers = min/max (excluding outliers, defined as <Q1 – 1.5*IQR or > Q3 + 1.5*IQR). **C**) ER ChIPseq – heatmap of read coverage at core ER binding sites in baseline samples (n = 22) and after letrozole (n = 7) or megestrol combination treatment (arm B n = 9, arm C n = 6). A window of ±5 kb region flanking the tag midpoint is shown. **D**) Normalised ER coverage intensity over a ± 2.5 kb region flanking the tag midpoint is shown for each trial arm, and for pre-treatment samples. **E** and **F**) One sample pair included in the Arm A ChIP-seq cohort was excluded from the trial primary/secondary endpoint analysis due to late reporting of HER2 status (borderline amplified). *HER2* amplification was not identified by whole genome sequencing for this patient. ChIP-seq analysis excluding this patient is presented - the pattern of decreased ER binding is maintained whether this sample pair was included or not (A vs B + C, p = 0.067, Mann Whitney U test). **E**) log transformed fold change in binding intensity across core ER sites (as defined in Ross-Innes et al 2012) for paired baseline and end of treatment samples, grouped by trial arm (n = 21 sample pairs). Box = IQR, centre = median, whiskers = min/max (excluding outliers, defined as <Q1 – 1.5*IQR or > Q3 + 1.5*IQR). **F**) Least squares lines summarising the distribution of baseline and EOT ER binding for patients in each trial arm (n = 21). **G**, **H**) Scatterplots showing the correlation of log2 transformed fold change in ER binding at core ER binding sites between Arm A vs Arm B (**G**) and Arm A vs Arm C (**H**). PCC = Pearson’s correlation coefficient, two-sided. Source data

As expected following antiestrogen therapy, we observed decreased ER binding in most participants but with a greater reduction relative to baseline seen in the megestrol combination arms (arm A versus arms B + C, *P* = 0.026, according to Mann–Whitney *U*-test) (Fig. 3b,c and Extended Data Fig. 2a–f). Almost all core ER-binding sites showed reduced ER binding following either dose of megestrol combination treatment, in contrast to a group of sites showing stable levels of ER-binding intensity following letrozole only (Extended Data Fig. 2g,h), suggesting that PR activation by megestrol has a quantitative impact on ER-binding potential, even at the lower dose of 40 mg. Importantly, this reduced ER binding was seen at conserved regulatory elements adjacent to known cell-cycle target genes, implying lower ER potential transcriptional activity following progestin treatment.

### Antiproliferative response in participant subgroups

**Fig. 4:**
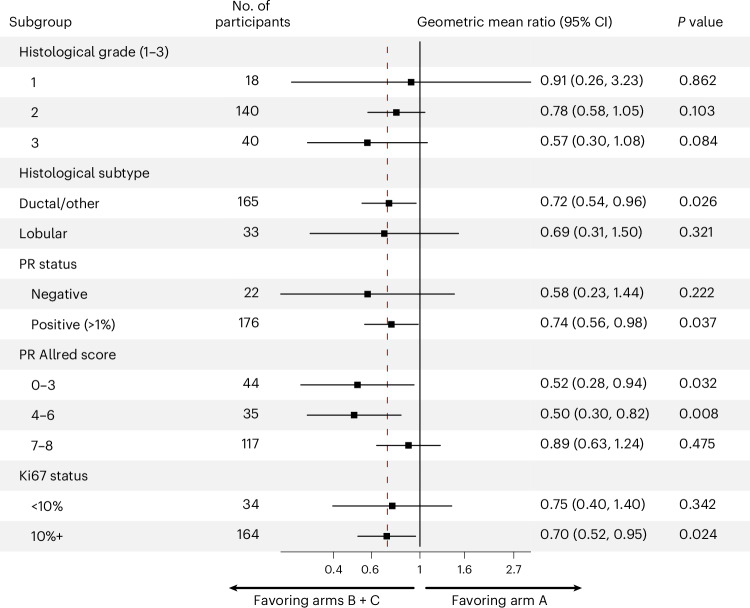
Antiproliferative response to treatment in participant subgroups. Exploratory analysis of response to treatment in different subgroups (GMR of proportional change in Ki67 in arm A versus arms B + C). The vertical red dotted line indicates the GMR in the overall trial population. Statistical analysis was conducted using two-sided *t*-tests; unadjusted *P* values are shown.

**Extended Data Fig. 3:**
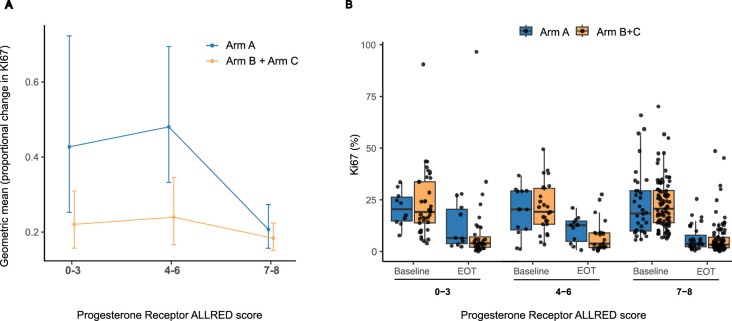
Anti-proliferative response to treatment in patient subgroups. **A**) Geometric means of proportional change in Ki67 in control (Arm A, n = 51 patients) vs megestrol combination treatment (Arms B + C, n = 145 patients) across different PR expression levels (Allred score, 0-3 (n = 44 patients), 4-6 (n = 35 patients), 7-8 (n = 117 patients)). Error bars are 95% CI. **B**) Boxplot of baseline vs EOT Ki67 (%) in subgroups with different PR expression levels (Allred score) in Arm A (n = 51 patients) vs B + C (n = 145 patients). Box = IQR, centre = median, whiskers = min/max (excluding outliers, defined as <Q1 – 1.5*IQR or > Q3 + 1.5*IQR). Source data

In a preplanned subgroup analysis of PR^+^ participants (*n* = 176), a greater reduction in Ki67 was observed in arms B + C compared to arm A, although the effect size was smaller than in the overall population (GMR: 0.74, 95% CI: 0.56–0.98, *P* = 0.038) (Fig. 4). PR positivity was defined as ≥1% positive cells by IHC^26^, which encompasses a broad range of PR expression levels. Further exploratory analysis suggested a trend toward participants with low or intermediate PR expression deriving greater benefit from the addition of megestrol to letrozole, in contrast to participants with the strongest PR expression (Allred 7–8) where the additional Ki67 suppression gained from inclusion of megestrol was limited (Fig. 4 and Extended Data Fig. 3a). There was no significant difference in baseline Ki67 percentages between participants with low or intermediate versus high PR expression (Extended Data Fig. 3b).

**Fig. 5:**
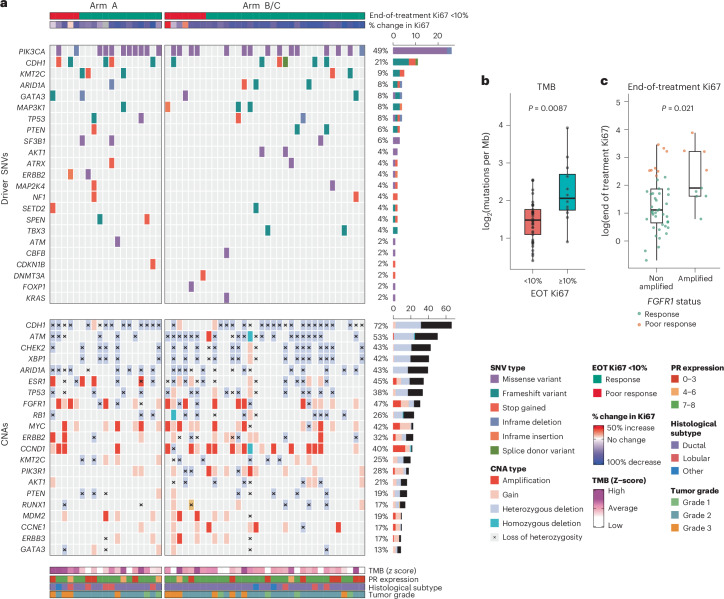
SNVs and CNAs in recurrently mutated breast cancer genes. **a**, Oncoplot depicting predicted driver SNVs (top) and CNAs (bottom) for 53 participants in genes previously described as recurrent or associated with antiestrogen resistance in ER^+^ breast cancer. For SNVs, in cases where participants had multiple driver mutations in a single gene, only the ‘most serious’ consequence is plotted. Samples are ordered by EOT Ki67. Right, bar plots showing the frequency of aberrations in the indicated genes across the whole cohort. **b**,**c**, Box plots of genome-wide TMB (log_2_ transformed) in good (*n* = 41) versus poor responders (*n* = 12) (defined as EOT Ki67 ≥ 10%) (**b**) and log-transformed EOT Ki67 values in tumors with *FGFR1* amplification (*n* = 9) versus nonamplified tumors (*n* = 44) (**c**). Data points are colored according to response (poor response: EOT Ki67 ≥ 10%). Statistical analysis was conducted using two-sided *t*-tests. Box, IQR; center, median; whiskers, min–max (excluding outliers, defined as <Q1 − 1.5 × IQR or >Q3 + 1.5 × IQR). Source data

**Extended Data Fig. 4:**
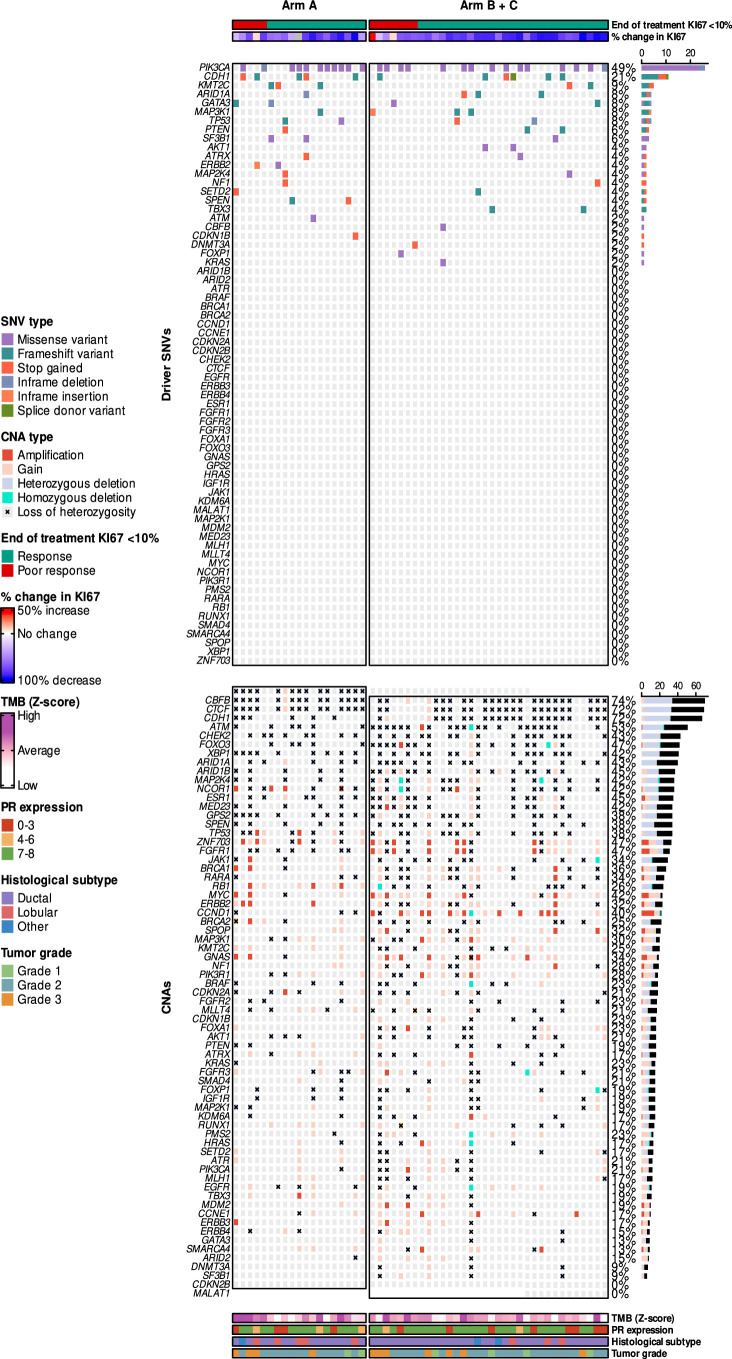
Somatic mutation frequencies in genes recurrently mutated in breast cancer. Visualisation of SNVs and CNAs across all interrogated genes in patients co-consented to a parallel tumor sequencing study (PBCP, n = 53 patients).

A total of 53/201 participants with paired or EOT Ki67 results available were co-consented to the Personalized Breast Cancer Program (PBCP) in Cambridge. Participants in this study underwent germline and somatic whole-genome sequencing. A set of somatic variations were analyzed and reported here (Methods). Clinical characteristics of this subset of participants are summarized in Supplementary Table 6. Our exploratory analysis focused on genes previously reported either as recurrently mutated in ER^+^ breast cancer or as associated with response or resistance to antiestrogen therapy (Fig. 5a and Extended Data Fig. 4). The frequencies of somatic variants in this gene set were in line with previously published cohorts^38–41^ with regard to *PIK3CA* (26/53, 49% of tumors with predicted pathogenic single-nucleotide variants (SNVs)), *CDH1* (inactivating mutations in 11/53 (21%) tumors, enriched for lobular histology), *KMT2C/MLL3* (5/53, 9%), *TP53* (4/53, 8%), *GATA3* (4/53, 8%), *MAP3K1* (4/53, 8%), *ARID1A* (4/53, 8%) and *PTEN* (3/53 tumors with inactivating mutations and associated loss of heterozygosity, plus ten cases with heterozygous deletions). As expected for a cohort of participants with early-stage breast cancer, no pathogenic *ESR1* mutations were detected (Extended Data Fig. 4).

**Extended Data Fig. 5:**
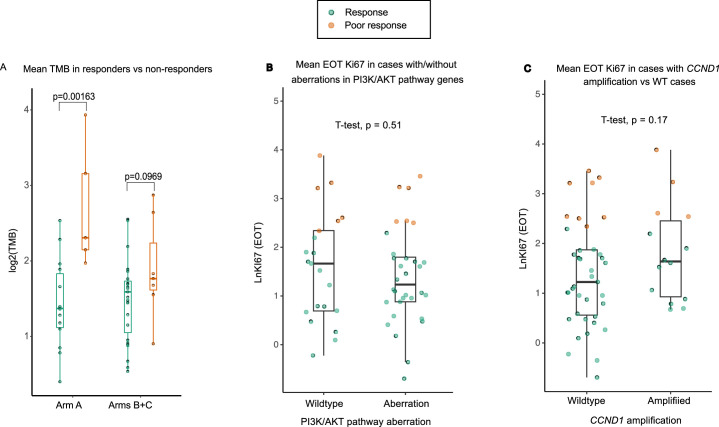
Molecular profiling of treatment response. Box plots showing: **A**) log2 transformed TMB in good (n = 41 patients) vs poor responders (n = 12 patients) (defined as EOT Ki67 ≥ 10%) in control vs megestrol combination treatment. Two-way ANOVA, with adjusted p-values (Tukey method), **B**) log transformed EOT Ki67in tumors with SNVs/CNVs in the PI3K/AKT pathway (n = 32/53, defined as predicted driver mutations in *PIK3CA, PIK3R1, AKT1, PTEN* or deletion of *PTEN/PIK3R1*) and **C**) Log transformed EOT Ki67 in tumors with (n = 14)/without (n = 39) amplification of *CCND1*. Data points are coloured according to whether EOT Ki67 was </≥ 10%. Statistic for panels B + C: two-sided t-test. Boxes = IQR, centres = median, whiskers = min/max (excluding outliers, defined as <Q1 – 1.5*IQR or > Q3 + 1.5*IQR). Source data

We explored whether reported genetic drivers of intrinsic resistance to antiestrogen therapy could explain poor responses to both control and combination treatment in PIONEER (Fig. 5a). We defined a poor response as failing to suppress Ki67 below 10% at EOT^30^. In keeping with previous reports^42,43^ tumor mutational burden (TMB), estimated across the whole genome, was higher in poor responders (mean TMB: 9.30 mutations per Mb versus 4.34 mutations per Mb in responders; *P* = 0.0087) (Fig. 5b). Poor response was also associated with higher TMB when participants receiving control and megestrol combination treatment were considered separately; however, this relationship only reached significance in control treated participants and the analysis was limited by the small number of poor responders (Extended Data Fig. 5a).

*FGFR1* amplification has been described as a mechanism of intrinsic resistance to AI therapy^44^. Participants with *FGFR1* amplification had a significantly higher EOT Ki67 compared to those without amplification (mean Ki67 EOT: 9.82% versus 3.71%; *P* = 0.021) (Fig. 5b). *FGFR1* was amplified in 4/12 (33%) of poor responders versus 5/41 (12%) of good responders (*P* = 0.18, Fisher’s exact test) (Fig. 5a). There was no significant association between aberrations in the PI3K–AKT–MTOR pathway and treatment response in this cohort (Fig. 5a and Extended Data Fig. 5b)^45^. Other established resistance mechanisms observed among poor responders included *MDM2* amplification^46^ (*n* = 2), *RB1* homozygous deletion (*n* = 1)^41^ and *ERBB2* (*n* = 2) and *ERRB3* (*n* = 1) amplifications^47^. Two participants with *ERBB2*-activating mutations (V777_G778insQGG and L755S) also had a limited response (Ki67 percentage change from baseline: +0.7% and −57%, respectively). *CCND1* amplification frequently co-occurred with *FGFR1* amplification (Fig. 5a) and has been described as a contributor to AI resistance in the *FGFR1*-amplified context^44^. However, *CCND1* amplification was not associated with a higher EOT Ki67 and did not occur more frequently in nonresponders (Extended Data Fig. 5c). Interestingly, two *GATA3* mutations were observed in poor responders, with a further mutation detected in a participant with a limited percentage change of −61% from baseline Ki67 (Fig. 5a). This is in contrast to previous reports describing *GATA3* mutations in association with a superior response to antiestrogen therapy^42,44^. Overall, these observations suggest that mechanisms of resistance to antiestrogen monotherapy may also apply to the response to combination therapy with megestrol.

## Discussion

PIONEER evaluated the antiproliferative effect of a progestogen (megestrol) combined with antiestrogen therapy (letrozole) in women with early-stage ER^+^ breast cancer. The trial met its primary endpoint, finding that adding megestrol enhanced the antiproliferative effect of letrozole. The two megestrol doses tested (lower, 40 mg; higher, 160 mg) showed comparable efficacy, with similar mean changes in the proliferation markers Ki67 and AURKA. Randomized controlled trials have reported 40 mg of megestrol as an effective means of treating hot flashes for persons taking antiestrogen therapy^10,11^, potentially helping with treatment adherence and thereby improving breast cancer outcomes^1,2^. The PIONEER trial results suggest that, in addition to this benefit, low-dose megestrol also has a direct antiproliferative effect when given in combination with an AI.

A lower mean Ki67 at EOT was observed after megestrol combination treatment, an endpoint that has been correlated with improved relapse-free survival in other studies of short-term presurgical endocrine therapy^29–31,48^. Multiple treatments for ER^+^ breast cancer have yielded positive results both in window-of-opportunity studies and in longer-term clinical trials, including AIs, PI3K inhibitors, CDK4/6 inhibitors and oral selective ER degraders^27,30,44,49–52^; the magnitude of Ki67 suppression with megestrol combination therapy appears similar to many of these treatments. As megestrol may also improve adherence to AIs, it is possible that the clinical benefit of treatment could be greater with longer-term use than the benefit suggested from improved suppression of Ki67. Since PIONEER recruitment opened, CDK4/6 inhibitors (for example abemaciclib) have become part of routine adjuvant breast cancer treatment for persons with higher-risk tumors. If found to be effective in longer-term trials, the addition of megestrol to adjuvant antiestrogen therapy could be an option for persons who do not tolerate CDK4/6 inhibitors. As megestrol is off-patent, it could also be a cost-effective option in settings in which CDK4/6 inhibitors are not affordable^53^.

Letrozole and megestrol are already in clinical use and are reasonably well tolerated as monotherapies^8,54^. Combination treatment with letrozole and megestrol demonstrated an acceptable safety profile at both lower and higher doses of megestrol, with similar rates of AEs to letrozole monotherapy. Grade 3 AEs were rare and most AEs were grade 1. VTE did not occur at above-expected rates for persons who had undergone breast cancer surgery^33^, although we cannot exclude that megestrol was a contributing factor to the two episodes of VTE observed. Hypertension was observed in participants receiving 160 mg of megestrol (arm C) but, importantly, not in participants taking lower-dose megestrol (40 mg; arm B). Although there was only a small difference in treatment-emergent hot flashes in arm A versus arms B + C, in previous trials, a longer treatment period was required to improve these symptoms^10^.

To assess the functional impact of megestrol combination therapy, we evaluated ER binding by ChIP-seq at a defined set of genomic loci that consistently bind ER across multiple distinct individual samples^37^. These sites represent a small proportion of ER-binding sites in any one participant but represent high-affinity regulatory regions that mediate canonical ER target gene expression^36,37^. We were able to map dynamic changes in ER binding at these loci between paired participant samples and could show that combining an AI with either dose of megestrol was sufficient to decrease ER binding. This implies a direct repression of ER activity by minimizing the genomic occupancy of ER at regulatory elements proximal to classical ER target genes such as *TFF1*, *XBP1* and *GREB1* (ref. ^37^).

There was extensive interparticipant heterogeneity in the number and location of ER-binding sites (as recently described^36^) and this limited our ability to interrogate PR-induced ER-binding events that could be seen in preclinical experimental conditions^24^. In contrast to the preclinical experiments, all participants received letrozole to decrease estrogen levels, resulting in lower ER binding and transcriptional activity. We did not assess global changes in gene expression by RNA sequencing; however, there is an established relationship between ER-binding levels and transcription^55^, supporting the conclusion that ER activity is suppressed to a greater degree after megestrol combination therapy. Accordingly, we observed greater repression of PR protein (a well-established ER target gene), an additional indicator of reduced ER activity in the megestrol combination arms. The repression of PR in all treatment arms (including arm A) highlights the importance of using diagnostic histology from core biopsy (rather than surgical histology) to guide adjuvant treatment for any participant treated preoperatively with antiestrogen therapy.

A planned subgroup analysis in participants with PR^+^ tumors showed similar results to the overall cohort. In an exploratory analysis, we observed similar responses to megestrol combination treatment across different PR expression levels; in contrast, participants with strongly PR^+^ tumors (Allred 7–8) responded better to letrozole alone compared to tumors with low or intermediate PR expression (Allred 0–3 or 4–6). This suggests that tumors with high ER and PR expression are enriched for those with exceptional sensitivity to AIs and, in this subgroup, there may only be marginal antiproliferative gain from adding megestrol.

The PIONEER trial had some key limitations. The short treatment duration in window studies allows only a limited assessment of safety and tolerability for megestrol in combination with letrozole. The primary endpoint did not compare the two different doses of megestrol independently against letrozole and any impact on clinical outcomes would have to be assessed in a larger, longer-term study adequately powered for disease-free and overall survival. Results suggesting that different PR expression levels (and other molecular features) may predict treatment response were unplanned exploratory analyses and will, therefore, need validation in future studies. The ChIP-seq analysis was only available for a small subset of participants.

PR was repressed to undetectable levels (<1% cells) in one quarter of PR^+^ tumors after treatment, raising the question of how and whether megestrol remains active in these tumors. A role for megestrol in inducing PR repression is suggested by the overrepresentation of arms B + C among tumors with treatment-induced PR negativity (*n* = 40/44 tumors, 91% in arms B + C). It is possible that initial megestrol-induced transcriptional reprogramming, coupled with an AI-induced fall in estrogen levels, inhibits ER activity such that PR expression is completely repressed and megestrol is no longer needed to inhibit ER in this subgroup. Alternatively, PR expression may be heterogeneous within individual tumors or there could be a residual low level of chromatin-bound PR, which is not detectable by IHC, in tumors that become PR^−^ during treatment. This could also explain some of the efficacy of megestrol combination treatment in PR^−^ (Allred 0) tumors.

Megestrol also has some affinity for the androgen and glucocorticoid receptors and preclinical data suggest that activation of these nuclear receptors is antiproliferative in ER^+^ breast cancer through a similar mechanism to PR ligands^56,57^. It is, therefore, possible that some megestrol activity is mediated through other nuclear receptors, although megestrol has generally been observed to have an antiandrogenic effect^58^ and AR antagonists have shown limited efficacy in ER^+^ disease^59^. Given the higher affinity of megestrol for PR^58^, the dominant mechanism for the antiproliferative effect of megestrol combination therapy is most likely through PR activation.

In conclusion, PIONEER has shown that combining megestrol acetate with aromatase inhibition has superior antiproliferative activity compared to an AI alone and was well tolerated over a short treatment window, particularly at the lower megestrol dose of 40 mg. These data support an evaluation of lower-dose megestrol combination therapy in further clinical trials, given its potential as a means of enhancing both the efficacy and the tolerability of AI therapy for persons with breast cancer.

## Methods

### Participants and study design

PIONEER is an open-label randomized phase 2b window-of-opportunity trial sponsored jointly by the University of Cambridge and Cambridge University Hospitals National Health Service Foundation Trust. The trial was approved by the UK Medicines and Healthcare Products Regulatory Agency and the Northeast Newcastle and North Tyneside 1 Research Ethics Committee (17/NE/0113) (NCT03306472, Eudra-CT 2016-003752-79, IRAS 210677). All participants provided written informed consent. All participants across all sites were assessed for eligibility criteria during their standard clinical evaluation. The trial was offered when it was considered clinically appropriate. There were no self-selection or site-based biases involved. Participants were able to apply for reimbursement for a contribution toward additional travel expenses associated with trial participation. The study design and conduct complied with all relevant regulations regarding the use of human study participants and was conducted in accordance with the criteria set by the Declaration of Helsinki. The study protocol is included in the Supplementary Information.

Postmenopausal women with treatment-naive early-stage (≥T1c, NX or N0–N3, M0) ER^+^ (Allred score ≥ 3), HER2^−^ breast adenocarcinoma were eligible to participate if they were scheduled for primary surgery or primary endocrine therapy either as neoadjuvant therapy or in lieu of surgery. The trial was confined to female participants because of the rarity of male breast cancer and the planned size of the trial. Menopausal status was defined as having experienced 12 months of natural (spontaneous) amenorrhea with an appropriate clinical profile (for example, ≥50 years, history of vasomotor symptoms) or 6 months of spontaneous amenorrhea with serum follicle-stimulating hormone and estradiol levels consistent with postmenopause or surgical bilateral oophorectomy (with or without hysterectomy) at least 6 weeks ago. Participants with PR− tumors were eligible, as megestrol treatment in the metastatic setting is given on the basis of ER but not PR expression and megestrol benefit in the treatment of hot flashes was not confined to those with PR^+^ disease. Exclusion criteria included known distant metastatic disease, Eastern Cooperative Oncology Group (ECOG) performance status of >2, use of hormone replacement therapy or use of tamoxifen or an AI (for a previous breast cancer diagnosis) in the previous 6 months, the presence of a progestogen-containing intrauterine system (unless removed before randomization), recurrent breast cancer and known serious disorders and contraindication or allergy to megestrol, letrozole or lactose.

Enrolled participants were randomized on a 2:3:3 ratio to one of three treatment arms: arm A (control), letrozole 2.5 mg only; arm B (research arm 1), letrozole 2.5 mg + lower-dose megestrol (40 mg); arm C (research arm 2), letrozole 2.5 mg + higher-dose megestrol (160 mg). Randomization was stratified by ER Allred score, histological subtype (ductal or lobular) and tumor grade using a minimization method with a random element. PR status was not included in the stratification as it is not routinely tested at diagnosis in all UK hospitals and central testing before randomization was not feasible in the short time frame before surgery. Treatment was given for 15 (13–19) days before either tumor excision or core biopsy. Baseline and EOT tissue was stained for the proliferation markers Ki67 and AURKA, PR, androgen receptor (AR) and cleaved caspase 3. The primary objective was to assess the change in tumor proliferation (measured by Ki67) between baseline and EOT in the combination arms (B and C) compared to the control arm (A). A planned subgroup analysis examined the effect of treatment specifically in PR+ participants (PR^+^ defined as ≥1% positive cells by IHC). Secondary endpoints were the comparison of Ki67 change in high-dose versus low-dose megestrol arms, absolute Ki67 at EOT and change in tumor apoptosis (cleaved caspase 3 IHC), proliferation (AURKA IHC), PR and AR expression and safety or tolerability. Any grade ≥3 toxicity was required to have resolved to grade 1 or less within 72 h; otherwise, participants were withdrawn from treatment. Exploratory analysis of ER chromatin binding (ChIP-seq) was conducted on paired fresh-frozen samples from a subset of participants.

### Primary human tissue

Core biopsies were obtained at baseline and EOT. Cores for IHC were fixed in 10% neutral buffered formalin for 24 h before embedding in paraffin wax. Cores for molecular analysis were stored immediately on ice and snap-frozen using either dry ice or liquid nitrogen within 30 min of collection. Fresh-frozen biopsies were stored at −80 °C until processed.

### IHC

Formalin-fixed paraffin-embedded sections (3–4 µm) from baseline and EOT were stained with hematoxylin and eosin. Further sections were stained for the following markers: Ki67 (clone MIB-1, Dako, M7240), AURKA (clone NCL-L-AK2, Novo Castra), PR (clone PgR 636, Dako, M3569), AR (clone AR441 M356201-1, Agilent, M3562), ER (clone EP1, Dako, M3643) and cleaved caspase 3 (D175) (Clone 5A1E, Cell Signaling, 9664). Antigen retrieval was heat mediated and all immunostaining was performed using a Leica bond max.

### Quantification of IHC

Slides were all scored centrally by a single expert histopathologist, blinded to treatment allocation and whether they were pretreatment or posttreatment samples. For Ki67/AURKA, tumor cell nuclei showing any intensity of staining were regarded as positive. Slides were reviewed at low power and four representative high-power fields (×40) were selected for counting; if there were clear areas with an increased proportion of cell staining (hot spots), these were included in the count. A total of 1,000 tumor cell nuclei were counted per slide; if there were <1,000 tumor cells in a biopsy, a minimum of 400 were counted. If there were <400 tumor cell nuclei in the research biopsy, then the diagnostic core biopsy or representative block from the surgical resection specimen was obtained and used for assessment. Ki67 and AURKA were scored as the percentage of tumor nuclei staining positive. Cleaved caspase 3, PR and AR were scored by visual estimation of the average percentage of positive cells across the tumor specimen.

### ChIP-seq

Flash-frozen core biopsies were cryosectioned into 10-µm sections before simultaneous thawing and fixation for 20 min in 2 mM disuccinimidyl glutarate followed by the addition of 1% methanol-free formaldehyde for a further 20 min. Crosslinking was quenched with 0.1 M glycine at room temperature and samples were then washed twice in ice-cold PBS. Extracted chromatin was fragmented using a probe sonicator (Fisher Scientific) until most DNA fragments were 100–800 bp. Chromatin was immunoprecipitated at 4 °C overnight using protein-A-bound (Invitrogen) Dynabeads with two well-validated specific antibodies to ER^60^ (a 50:50 mixture of Millipore, 06-935 and Abcam, ab3575). After washing of the beads, chromatin was eluted and decrosslinked by incubating overnight at 65 °C in elution buffer (50 mM Tris-HCl pH 8, 10 mM EDTA and 1% SDS). Samples were treated with (20 ng ml^−1^) for 30 min followed by (200 ng ml^−1^) for 1–2 h before DNA was purified by phenol–chloroform extraction. Purified DNA was subjected to library preparation using the SMARTer ThruPLEX DNA644 Seq kit (TaKaRa, R400676) and DNA HT dual index kit 96N set A (TaKaRa, R400660), followed by Illumina next-generation sequencing to reach approximately 20 million reads per sample. ChIP-seq analyses of 50-bp single-end reads were mapped to the hg38 genome using bowtie2 (version 2.2.6)^61^. Aligned reads with mapping quality < 5 were filtered out. To internally control for successful ChIP-seq, sample pairs with fewer than 500 (in baseline samples) or 250 valid ER-binding sites (in EOT samples, because of the expected decrease in ER binding with AI treatment) were eliminated from subsequent analyses and likely represent samples with insufficient tumor material for ChIP-seq.

### DNA sample collection and processing

In a subset of participants participating in the ongoing PBCP, blood samples were collected using 9-ml EDTA-coated Vacutainer tubes (Sarstedt 02.1066.001) without gel separators. Immediately after collection, tubes were gently inverted 5–6 times to ensure proper mixing and centrifuged at 1,600*g* for 10 min at room temperature. The centrifugation used high-speed acceleration and low-speed braking to minimize disruption of the buffy coat. Using a pipette, 0.5 ml of the buffy coat was transferred to DNase-free and RNase-free 2-ml screw-cap tubes and stored at −80 °C. Fresh tumor biopsies were performed using a 14-gauge biopsy needle and transferred on wet ice to the preparation area. Tissue samples were placed in 2-ml microtubes and either snap-frozen in liquid nitrogen for a minimum of 5 min or moved directly to a −80 °C freezer within 30 min of resection.

#### Nucleic acid isolation and quantification

DNA was isolated from tumor biopsies using the Qiagen AllPrep DNA/RNA micro kit (80204) at the Cambridge Cancer Molecular Diagnostics Lab. Two core biopsies were combined for each extraction to ensure a minimum of 20% tumor cells. For homogenization, OCT-embedded samples were first dissolved in 1 ml of distilled water, followed by transfer into 2-ml tubes containing a 5-mm stainless-steel bead and 600 μl of RLT plus buffer. Homogenization was conducted using the TissueLyser at 25 Hz for two 1-min rounds. Germline DNA was extracted from 200 μl of the buffy coat using the DSP DNA mini kit (61304) on a QIAsymphony instrument (Qiagen). The concentration of DNA samples was assessed using the Qubit assay kits (Thermo Fisher Scientific) and Tapestation (Agilent Technologies).

#### Library preparation and sequencing

DNA samples were quantified using the SpectraMax Gemini XPS (Molecular Devices). PCR-free libraries for sequencing were prepared using the TruSeq DNA PCR-free library preparation kit (Illumina). For samples with low DNA input, the TruSeq Nano DNA library preparation kit was used with three cycles of PCR amplification. Sequencing was performed on the Illumina HiSeqX platform, generating paired-end reads (2 × 150 cycles). The target coverage was >30× for germline samples and >75–100× for tumor samples. Samples with an estimated tumor content < 10% (*n* = 4) were excluded from downstream analyses.

#### Variant calling and annotation

DNA sequence reads were aligned to the human reference genome GRCh38 with decoys using the Isaac aligner (version 03.16.02.19). Germline SNVs and indels were identified using Strelka (version 2.4.7) for small variants (≤50 bp). Somatic variants in the tumor were called using a joint-calling mode with matched normal DNA. To annotate variants and determine their driver status, we used the Cancer Genome Interpreter (version 23.12.2), with a configuration tailored to breast cancer.

To calculate TMB, we first quantified the total number of somatic mutations, including base substitutions and indels, detected across the tumor genome. This total was then normalized by dividing by the effective coverage area, expressed in Mb of genome sequenced. This normalization process provides a TMB score expressed as the number of mutations per Mb, allowing for comparisons across samples with varying genomic coverage.

#### Copy-number alteration (CNA) analysis

CNAs were analyzed using Canvas (version 1.3.1.012), applying a methodology designed to correct for sample-specific noise and genomic variability. Copy-number values were normalized by first applying the formula copy number/(ploidy/2) and then rounding the results to the nearest whole number to facilitate categorization. Categories were defined as follows: values of ≥4 were classified as amplifications; values of 3–4 were categorized as gains; values of 2–3 were considered neutral and values of 0–2 were defined as heterozygous deletions. A copy number of 0, absent any SNV in the same gene, was classified as a homozygous deletion. If SNVs were present, the classification was adjusted to heterozygous deletion.

A set of genes previously described as recurrent or associated with antiestrogen resistance in ER^+^ breast cancer was used to inform on SNVs and CNAs: *AKT1*, *ARID2*, *ARID1A*, *ARID1B*, *ATM*, *ATR*, *ATRX*, *BRAF*, *BRCA1*, *BRCA2*, *CBFB*, *CCND1*, *CCNE1*, *CDH1*, *CDKN1B*, *CDKN2A*, *CDKN2B*, *CHEK2*, *CTCF*, *DNMT3A*, *EGFR*, *ERBB2*, *ERBB3*, *ERBB4*, *ESR1*, *FGFR1*, *FGFR2*, *FGFR3*, *FOXA1*, *FOXO3*, *FOXP1*, *GATA3*, *GNAS*, *GPS2*, *HRAS*, *IGF1R*, *JAK1*, *KRAS*, *KDM6A*, *KMT2C*, *MALAT1*, *MAP2K1*, *MAP2K4*, *MAP3K1*, *MDM2*, *MED23*, *MLH1*, *MLLT4*, *MYC*, *NCOR1*, *NF1*, *RB1*, *RARA*, *RUNX1*, *PIK3CA*, *PIK3R1*, *PMS2*, *PTEN*, *SETD2*, *SF3B1*, *SMAD4*, *SMARCA4*, *SPEN*, *SPOP*, *TBX3*, *TP53*, *ZNF703* and *XBP1*.

For visualization of SNVs and CNAs across samples, we used the Complex Heatmap (version 2.15.4) package in R (version 4.3.3).

### Statistical analysis

The study used an enrichment design with an overall significance level of 5% (one-sided) (5% (α) = 2.5% (αall) + 2.5% (αPR^+^)) and a power of 80%. Assuming a common s.d. of 0.242, a two-sample *t*-test comparing arm A versus arms B + C required a total of 189 participants to detect a 66% reduction in arm A and 77.5% reduction in arms B + C for Ki67 according to previous reports^27,48^ and a total of 149 PR^+^ participants to detect a mean reduction of 66% in arm A and 80.0% in arms B + C for Ki67.

The trial recruited sufficient participants to proceed with the planned analysis. Ki67 analyses were performed on a per-protocol population (the evaluable population), including all participants that completed at least 13 days of study treatment, with paired (baseline and EOT) Ki67 assessment available. Slides were all scored centrally for Ki67 by a single expert histopathologist, blinded to treatment allocation and whether they were pretreatment or posttreatment samples. EOT Ki67 values were adjusted by a factor of 1.15 if they were scored from a surgical excision specimen because of inadequate cellularity or unavailability of a core biopsy, as described previously^30^. The primary endpoint was the change in Ki67, based on the GMR of the proportional changes (ratio of EOT and baseline Ki67) between groups. Geometric means were used because of the typically lognormal distribution of Ki67 data. Ki67 suppression was defined as the geometric means of the proportional changes − 1. For analyses requiring log transformation, a single participant in arm C with an EOT Ki67 value of zero was excluded. Safety analyses included all participants who had received at least one dose of study treatment.

Secondary analyses of Ki67 included geometric mean EOT Ki67, a comparison of low-dose and high-dose megestrol, and exploratory analysis of the proportion of participants responding to treatment, with response defined as Ki67 < 10% on day 15 (refs. ^29–31^). Other secondary endpoints included changes in expression of cleaved caspase 3, AURKA, AR and PR. For analyses requiring log transformation, 0.0001 was added to AURKA scores, because of a high frequency of zero values at EOT. In total, six participants (two arm B and four in: n = arm C) with no AURKA expression at baseline were excluded from analyses of change in AURKA expression between baseline and day 15. The comparison of the geometric means of AURKA proportional changes and Ki67 on day 15 was based on a *t*-test. The comparison of the difference in cleaved caspase 3, AR and PR on day 15 and baseline assessment were based on the Mann–Whitney *U*-test. The proportion of responders (Ki67 < 10%) was compared using a chi-squared test. The 95% CI of the response rate was based on the Clopper–Pearson method and the difference in the proportion was based on 1,000 bootstrap samples. All statistical tests were two-sided and analysis was performed with R (version 4.3.1). The data met the assumptions of the statistical tests used, with formal testing for normality and equal variance where appropriate. CONSORT guidelines^62^ were followed for the reporting of this trial (Supplementary Information).

### Reporting summary

Further information on research design is available in the Nature Portfolio Reporting Summary linked to this article.

## Supplementary information

Supplementary Information Full protocol for the PIONEER Trial, CONSORT Checklist for the PIONEER Trial and PBCP study group and extended acknowledgements for the PBCP.

Reporting Summary

Supplementary Tables Supplementary Tables 1–10.

## Source data

Source Data Figs. 2, 3 and 5 and Extended Data Figs. 1–3 and 5 Statistical source data.

## Data Availability

ChIP-seq data that support the findings of this study were deposited to the Gene Expression Omnibus under accession code GSE296953. Data collected within the PIONEER study will be made available to researchers whose full proposal for their use of the data has been approved by the PIONEER Trial Management Group and whose research includes a clear and comprehensive research plan with statistical considerations adequately completed. The data required will be provided for the approved, specified purposes after completion of a data sharing agreement. Data sharing agreements will be set up by the Trial Management Group and will include clear instructions on publication, reporting and usage policy. A minimum dataset of anonymized data will be made available after full publication of the trial and related work. Requests for data should be addressed to R.D.B. (rdb39@cam.ac.uk). Source data are provided with this paper.

## Code availability

Code was deposited to GitHub (https://github.com/igorchern/jclabcode).
