# Supplementary material for: Evaluating progesterone receptor agonist megestrol plus letrozole for women with early-stage estrogen-receptor-positive breast cancer: the window-of-opportunity, randomized, phase 2b, PIONEER trial
Source: Nat Cancer. 2026 Jan 5;7(1):194–206. doi: 10.1038/s43018-025-01087-x (PMC12858400; doi:10.1038/s43018-025-01087-x)
Supplement: Supplementary file 1 — Full protocol for the PIONEER Trial, CONSORT Checklist for the PIONEER Trial and PBCP study group and extended acknowledgements for the PBCP. [file 43018_2025_1087_MOESM1_ESM.pdf]

nature cancer

Supplementary information

https://doi.org/10.1038/s43018-025-01087-x

Evaluating progesterone receptor agonist megestrol plus letrozole for women with early-stage estrogen-receptor-positive breast cancer: the window-of-opportunity, randomized, phase 2b, PIONEER trial

In the format provided by the authors and unedited

Clinical Trial Protocol-

PIONEER

Randomised Phase II clinical trial **PIONEER-** A **P**re-operative w**I**nd**O**w study of Letrozole plus PR agonist (Megestrol Acetate) versus Letrozole alo**NE** in post-menopausal patients with **ER**-positive breast cancer

EudraCT Number: 2016-003752-79

ISRCTN Number: 15621797

Investigational Product: Letrozole and Megestrol Acetate

Protocol Version: PIONEER_Protocol_v9.0_28.03.2022

Chief Investigator: Dr Richard Baird

CI Address: Breast Cancer Research Unit, Box 97, Addenbrooke's Hospital
Hills Rd, Cambridge, CB2 0QQ

Telephone: +44 (0) 1223 768434

Trial Sponsor: Cambridge University Hospitals NHS Foundation Trust and the University of Cambridge
R&D Department, Box 277, Addenbrooke's Hospital
Hills Road, Cambridge, CB2 0QQ
✉ research@addenbrookes.nhs.uk

SAE Reporting: PIONEER Office – CCTU – Breast Cancer Theme
Direct line: +44(0) 1223 348073

✉ pioneer@addenbrookes.nhs.uk

1 Protocol Signatures:

I give myapproval for the attached protocol entitled PIONEER dated 28.03.2022

Chief Investigator

Name: Richard Baird

Signature: ____

Date: ____

Site Signatures

I have read the attached protocol entitled 'Randomised Phase II clinical trial **PIONEER**-A **P**re-operative w**I**nd**O**w study of Letrozole plus PR agonist (Megestrol Acetate) versus Letrozole alo**NE** in post-menopausal patients with **ER**-positive breast cancer' dated 04.02.2020and agree to abide by all provisions set forth therein.

I agree to comply with the conditions and principles of Good Clinical Practice as outlined in the European Clinical Trials Directives 2001/20/EC and the GCP Directive 2005/28/EC.

I agree to ensure that the confidential information contained in this document will not be used for any other purpose other than the evaluation or conduct of the clinical investigation without the prior written consent of the Sponsor

Principal Investigator

Name: ____

Signature: ____

Date: ____

2 Trial Management Committee and Protocol Contributors

Chief Investigator: Dr Richard Baird
Phone: +44 (0)1223 768434
e-mail: rdb39@medschl.cam.ac.uk

Co-Investigator (Translational): Dr Jason Carroll
Phone: +44 (0)1223 769 64
e-mail: Jason.Carroll@cruk.cam.ac.uk

Coordinating Investigator: Dr Rebecca Burrell
Phone: +44 (0)1223 769649
e-mail: rebecca.burrell@addenbrookes.nhs.uk

Senior Trial Coordinator: Gemma Young
Phone: +44 (0)1223 256022
e-mail:gemma.young@addenbrookes.nhs.uk

Trial Coordinator: Tulay Gulsen
Phone: +44 (0)1223 348073
e-mail: tulay.gulsen@addenbrookes.nhs.uk

Lead Breast Histopathologist: Dr Elena Provenzano
Phone: +44 (0)1223 256154
e-mail: elena.provenzano@addenbrookes.nhs.uk

Trial Statistician: Nikos Demiris
Phone: +44 (0)1223 256363
e-mail: Nikos.Demiris@addenbrookes.nhs.uk

Trial Pharmacist: Anita Chhabra
Phone: +44 (0)1223 596233
e-mail: anita.chhabra@addenbrookes.nhs.uk

Previous protocol contributors:

Greig Dougall
Wendi Qian
Angels Kateb Castellnou
Louise Grybowicz
Dr Sanjeev Kumar

Table of Contents

1 Protocol Signatures: ... 2
2 Trial Management Committee and Protocol Contributors ... 3
Table of Contents ... 4
3 Abbreviations ... 6
4 Trial Synopsis ... 8
5 Trial Flow Chart ... 13
6 Introduction ... 14
6.1 Background ... 14
7 Rationale for Trial ... 14
7.1 Data from non-clinical trials ... 14
7.2 Clinical Data ... 16
8 Trial Design ... 17
8.1 Statement of design ... 17
8.2 Number of Centres ... 17
8.3 Number of Subjects ... 17
8.4 Subjects Trial duration ... 17
8.5 Trial Objectives ... 18
8.6 Trial Endpoints ... 18
9 Selection and withdrawal of subjects ... 19
9.1 Inclusion Criteria ... 19
9.2 Exclusion Criteria ... 20
9.3 Treatment Assignment and Randomisation Number ... 20
9.4 Discontinuation of trial treatment and patient withdrawal ... 20
10 Procedures and assessments ... 21
10.1 Screening evaluation ... 22
10.2 Baseline assessments – Prior to Treatment (Day -15 to Day 1) ... 23
10.3 Trial assessments during protocol treatment ... 23
10.4 Long-Term Follow-up Assessments ... 25
10.5 End of Trial Participation ... 25
10.6 Schedule of Assessments ... 26
11 Trial Treatments ... 27
11.1 Treatment summary ... 27
11.2 Maximum duration of treatment of a patient ... 27
11.3 Procedures for monitoring treatment compliance ... 28
11.4 Supply, accountability and dispensing ... 28
11.5 Investigational Medicinal Products ... 29
11.6 Letrozole ... 29
11.7 Megestrol acetate 40mg tablets ... 30
11.8 Megestrol acetate 160mg tablets ... 31
11.9 IMP delivery ... 32
11.10 Dosage modifications ... 32
11.11 Concomitant therapy ... 32
12 Assessment of Safety ... 33
12.1 Definitions ... 33
12.2 Expected Adverse Reactions/Serious Adverse Reactions (AR /SARs) ... 34
12.3 Expected Adverse Events/Serious Adverse Events (AE/SAE) ... 34
12.4 Evaluation of adverse events ... 35
12.5 Reporting serious adverse events ... 35
12.6 Reporting of Suspected Unexpected Serious Adverse Reactions (SUSARs) ... 36

13 Evaluation of Results (Definitions and response/evaluation of outcome measures) ... 37
13.1 Trial Outcome Measures ... 37
14 Statistics ... 39
14.1 Statistical methods ... 39
14.2 Number of Subjects to be enrolled ... 39
14.3 Enriched data monitoring ... 40
14.4 Criteria for the premature termination of the trial ... 40
14.5 Procedure to account for missing or spurious data ... 40
14.6 Definition of the end of the trial ... 41
15 Data handling and record keeping ... 41
15.1 Case Report Form (CRF) ... 41
15.2 Source Data ... 41
15.3 Data Protection & Patient Confidentiality ... 42
15.4 Conduct of trial ... 42
16 Storage and Analysis of Samples ... 42
17 Independent Data Monitoring Committee/Trial Steering Committee ... 43
17.1 Trial Management Team (TMT) ... 43
17.2 Trial Management Group (TMG) ... 44
17.3 Independent Data Monitoring Committee (IDMC) ... 44
17.4 Trial Steering Committee (TSC) ... 44
17.5 Relationship between Trial Committees ... 44
18 Ethical & Regulatory considerations ... 45
18.1 Consent ... 45
18.2 Ethical committee review ... 45
18.3 Regulatory Compliance ... 45
18.4 Protocol Amendments ... 46
18.5 Peer Review ... 46
18.6 Declaration of Helsinki and Good Clinical Practice ... 46
18.7 GCP Training ... 46
19 Sponsorship, Financial and Insurance ... 46
20 Monitoring, Audit & Inspection ... 46
21 Protocol Compliance and Breaches of GCP ... 47
22 Publications policy ... 47
23 References ... 48
24 Appendices ... 51
Appendix 1 - Safety Reporting Flow Chart ... 51
Appendix 2 - ECOG performance status ... 52
Appendix 3 - TNM Staging System for Breast Cancer ... 53
Appendix 4 – ^{18}F-FLT PET/MR Imaging Sub-Study (Addenbrooke's Hospital only) ... 55

3 Abbreviations

AE	Adverse Event
AI	Aromatase Inhibitor
ALT	Alanine Aminostransferase
ALP	Alkaline phosphatase
AR	Adverse Reaction
ANC	Absolute Neutrophil Count
CCTU-CT	Cambridge Clinical Trials Unit – Cancer Theme
ChIP-seq	Chromatin Immunoprecipitation-sequencing
CI	Chief Investigator
CRF	Case Report Form
CRUK	Cancer Research UK
CTA	Clinical Trial Authorisation
CTCAE	Common Terminology Criteria for Adverse Events
DNA	Deoxyribose Nucleic Acid
DSUR	Development Safety Update Report
ECOG	Eastern Cooperative Oncology Group
ED	Effective dose
ER	Oestrogen receptor
ERα	Oestrogen receptor alpha
FLT	Fluorothymidine
FSH	Follicle Stimulating Hormone
GCP	Good Clinical Practice
GMP	Good Manufacturing Practice
GP	General Practitioner
HER2	Human Epidermal Growth Factor Receptor 2
HRA	Health Research Authority
IC	Informed Consent
IDMC	Independent Data Monitoring Committee
IHC	Immuno-histochemistry
IMP	Investigational Medicinal Product
MDT	Multi-disciplinary team
MHRA	Medicines and Healthcare products Regulatory Agency
MRI	Magnetic Resonance Imaging
NHS	National Health Service
PET	Positron Emission Tomography
PI	Principal Investigator
PIS	Patient Information Sheet
PR	Progesterone Receptor
R&D	Research and Development
REC	Research Ethics Committee
RSI	Reference Safety Information
SAE	Serious Adverse Event
SAR	Serious Adverse Reaction
SERD	Selective Oestrogen Receptor Downregulator
SERM	Selective Oestrogen Receptor Modulator
SmPC	Summary of Product Characteristics
SOP	Standard Operating Procedures
SUSAR	Suspected Unexpected Serious Adverse Reaction

SUV	Standardised uptake value
TMG	Trial Management Group
TNM	Tumour Node Metastasis (Classification)
TSC	Trial Steering Committee
TV	Tumour volume
UK	United Kingdom
USA	United States of America
ULN	Upper Limit of Normal
WBIC	Wolfson Brain Imaging Centre

4 Trial Synopsis

Title of clinical trial	Randomised phase II clinical trial **PIONEER**- **P**re-operative w**I**nd**O**w study of Letrozole plus PR agonist [Megestrol Acetate] versus Letrozole alo**NE** in post-menopausal patients with **ER**-positive breast cancer
Sponsor name	Cambridge University Hospitals NHS Foundation Trust and the University of Cambridge
EudraCT number	2016-003752-79
Medical condition or disease under investigation	Early-stage, Oestrogen receptor (ER) positive, Human Epidermal Growth Factor receptor 2 (HER2) negative, ≥1cm, invasive primary breast cancer in post-menopausal women.
Purpose of clinical trial	To test if the addition of Megestrol Acetate increases the anti-proliferative effect of Letrozole when given for 15 days pre-operatively in patients with early-stage, ER-positive breast cancer.
Primary Objective:	To determine if the addition of Megestrol Acetate increases the anti-proliferative effect of Letrozole when given for 15 days pre-operatively in patients with early-stage, ER-positive breast cancer, as measured by change in Ki67.
Secondary Objectives:	• To compare and correlate the biological effects of Letrozole alone compared to Letrozole plus Megestrol Acetate using other immunohistochemical markers of tumour response: Caspase 3, Aurora kinase A, change in expression of the androgen and progesterone receptors, and the absolute value of Ki67 at Day 15<br>• To compare and correlate the change in Ki67 and the biological effects of low dose Megestrol Acetate compared to high dose Megestrol Acetate using other immunohistochemical markers of tumour response: Caspase 3, Aurora kinase A, change in expression of the androgen and progesterone receptors, and the absolute value of Ki67 at Day 15<br>• To assess the safety and tolerability of the combination of Letrozole +/- Megestrol Acetate by recording and assessing adverse and serious adverse events.
Exploratory Objectives:	• To assess progestin-induced ER reprogramming following treatment with Letrozole and Megestrol Acetate, using transcription factor mapping (ChIP-seq) of ER, and RNA sequencing.<br>• To compare and correlate the biological effects of Letrozole alone compared to Letrozole plus Megestrol Acetate and also the effects of low dose Megestrol Acetate compared to high dose Megestrol Acetate using epithelial mesenchymal transition markers.<br>• To correlate PIONEER efficacy and exploratory findings of this trial with breast cancer genomic profiling datasets.<br>• Optional Imaging sub-study (Addenbrooke's patients only): to noninvasively assess the anti-proliferative

	effect of Letrozole vs. Letrozole + Megestrol Acetate using 18-FLT-PET/MRI imaging indices, and to correlate this with the changes observed in Ki67.
Primary endpoint:	Change in tumour proliferation measured by Ki67 immuno-histochemical (IHC) assessment (%) between baseline and day 15 (+≤4 Days).
Secondary endpoints:	**Biological Endpoints**<br>• Change in tumour apoptosis between baseline and Day 15 (+≤4 Days), measured by Caspase 3 (IHC)<br>• Change in expression of androgen receptor and progesterone receptor (PR) (IHC) between baseline and Day 15 (+≤4 Days)<br>• Change in proliferation by Aurora Kinase A (IHC) between baseline and Day 15 (+≤4 Days)<br>• Absolute value of Ki67 at Day 15 (+≤4 Days)<br>**Safety Endpoints**<br>• Incidence of Serious Adverse Events (SAEs)<br>• Incidence of Adverse Events (AEs) of all grades (CTCAE Version 4.03)
Exploratory endpoints:	• Transcription factor mapping (ChIP-seq) of ER.<br>• Gene expression changes associated with treatment, measured by RNA-sequencing.<br>• Change in epithelial mesenchymal transition markers (IHC) between baseline and Day 15 (+≤4 Days)<br>• Correlate differences in response to treatments with breast cancer genome profiling datasets.<br>• Changes in tumour proliferation measured by ^{18}FLT-PET/MRI (optional imaging sub-study only) and correlate these changes with changes observed in Ki67.
Trial Design	A three-arm, open label, multi-centre randomised phase II pre-surgical window trial
Sample Size	N = 189 evaluable patients
Summary of eligibility criteria	**Inclusion Criteria:**<br>• Histologically confirmed breast adenocarcinoma<br>• Postmenopausal women<br>• Core biopsy confirmation of invasive carcinoma on core biopsy, ≥T1c, either clinical NX or N0-N3<br>• ER positive (Allred≥3) and HER2 negative<br>• 2 groups of patients are potentially eligible:<br>o Cohort A: Patients whose cancers have been deemed to be operable by the Multi-Disciplinary Team (MDT), with tumour excisionplanned for the next 2-6 weeks<br>o Cohort B: Patients with early or locoregionally advanced breast cancer planned for primary endocrine therapy, either in lieu of tumour excision or as neoadjuvant therapy prior to tumour excision– such patients must begin PIONEER trial therapy prior to starting any other endocrine therapy.<br>• ECOG performance status of 0, 1 or 2<br>• Adequate Liver, Renal and Bone marrow function, defined as:

<table>
<tr><td></td><td>
- Adequate liver function where bilirubin is ≤1.5 x ULN
- Adequate renal function with serum creatinine ≤ 1.5 x ULN
- Adequate bone marrow function with ANC ≥1.0 x 10^9/L and Platelet count ≥100 x 10^9/L
• Written informed consent (IC) to participate in the trial and to donation of tissue.

Exclusion Criteria
• History of hormone replacement therapy in the last 6 months
• Previous treatment with Tamoxifen or an aromatase inhibitor in the last six months
• Known hypersensitivity or contraindications to aromatase inhibitors or Megestrol acetate
• Known allergy to lactose
• Known to have a progestogen-containing intrauterine system in situ, unless removed prior to randomisation
• Known metastatic disease on presentation
• Recurrent breast cancer (patients with a <u>new</u> primary invasive breast cancer will be eligible to participate)
• Serious concomitant disorders that would compromise the safety of the patient or compromise the patient's ability to complete the trial, at the discretion of the investigator
• Treatment with an investigational drug within 4 weeks before randomisation
• Inability to swallow orally administered medication and patients with gastrointestinal disorders likely to interfere with absorption of the trial medication
• Inability to give informed consent (IC)
</td></tr>
<tr><td>Investigational medicinal product and dosage:</td><td>Patients will be randomised into 1 of 3 arms:
Arm A: 15 days of Letrozole 2.5mg daily
Arm B: 15 days of Letrozole 2.5mg daily + Megestrol acetate 40mg daily
Arm C: 15 days of Letrozole 2.5mg daily + Megestrol acetate 160mg daily.</td></tr>
<tr><td>Route(s) of administration</td><td>Oral</td></tr>
<tr><td>Maximum duration of treatment of a participant</td><td>15 days (+ ≤4 days) of single agent or combined endocrine therapy followed by tumour excision and/or core biopsy.</td></tr>
</table>

Screening & enrolment	Potentially eligible patients with histologically confirmed ER-positive, HER2-negative early breast cancer will be approached and consented.<br><br>Screening procedures include:<br>• Standard assessment prior to tumour excision, including blood tests<br>• ECOG performance score<br>• Standard staging to exclude metastatic disease if this is deemed clinically appropriate by the investigator<br>• Patients will be stratified as part of the randomisation based on their tumour characteristics: tumour grade, ductal vs. lobular subtype, and intensity of ER staining on IHC<br><br>When eligibility is confirmed, patients will be randomised via a web-based central system to one of the three treatment arms.
Baseline	Patients will have to undergo standard assessments prior to treatment.<br><br>Standard assessments include:<br>• Baseline symptoms<br>• Weight in kg and height in cm<br>• AEs/SAEs and concomitant medication assessment<br><br>If patient does not have available diagnostic tissue block, a pre-treatment research core biopsy of breast tumour will be obtained.
Treatment period	Main assessments/procedures during treatment period:<br>• Patient treatment compliance check by telephone on Day 5 (+/- 1 day) and Day 10 (+/- 1 day)<br>• AEs/SAEs and concomitant medication assessment by telephone on Day 5 (+/- 1 day), Day 10 (+/- 1 day), and Day 15 (+≤4 days)<br>• Research core biopsy collection and/or breast tumour excision on Day 15 (+≤4 days)<br><br>Post-treatment follow up visit/phone call on Day 28 (+/- 5 days)<br>• ECOG performance status and weight (if visit performed on site)<br>• Review to assess AEs/SAEs<br>• Concomitant medications
End of Trial	The end of trial will be 12 months after the last patient's last visit, which will allow sufficient time for the translational endpoints to be investigated and the data cleaned for primary analyses and reports.

Procedures for safety monitoring during trial	The Trial Management Group and the Independent Data and Safety Monitoring Committee will regularly review the patient safety data. Pharmacovigilance will be performed by the PIONEER Trial Office.
Criteria for withdrawal of patients from protocol treatment	- Severe toxicity or inter-current illness, requiring cessation in the judgement of the treating clinician.<br>- Patient unable to complete at least 13 of the full 15 days of allocated treatment.<br>- Patient unable to comply with trial procedures.<br>- Disease progression while on trial treatment.

5 Trial Flow Chart

2 groups of patients are potentially eligible for the PIONEER trial:

- Cohort A: Patients whose cancers have been deemed to be operable by the Multi-Disciplinary Team (MDT) with tumour excision planned for the next 2-6 weeks

- Cohort B: Patients with early or locoregionally advanced breast cancer planned for primary endocrine therapy either in lieu of tumour excision or as neoadjuvant therapy before tumour excision - such patients must begin PIONEER trial therapy prior to starting any other endocrine therapy.

For all patients who consent to enter PIONEER and are planned for tumour excision, a date for tumour excision should be booked prior to randomisation. The date of tumour excision will determine when the patient should start treatment, as the day of tumour excision should coincide with Day 15 (+≤4 days) of treatment.

For patients who consent to enter PIONEER but are planned for primary endocrine therapy, the date the patient should start trial treatment must be as soon as it is feasible.

Patients will be stratified according to their baseline characteristics of histological tumour grade (1,2,3), ductal vs. lobular subtype, and intensity of ER staining on IHC of their initial, diagnostic core biopsy. In terms of ER status, patients will be stratified on the basis of the Allred ER score (3-6 moderate, 7-8 strong). This will allow even distribution of these characteristics between the three randomized arms.

Figure 1.

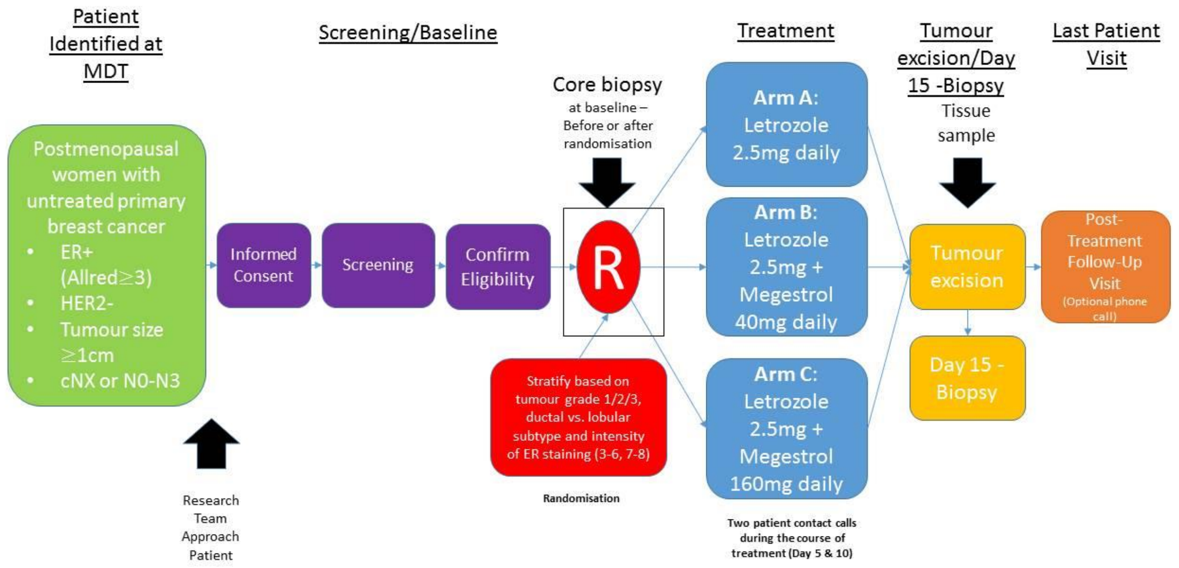

Treatment in all arms A/B/C will be for 15 days (+ ≤4 days if required).

6 Introduction

6.1 Background

Breast cancer is the most common cancer in women worldwide, and the second most common cancer overall, with more than 1,676,000 people newly diagnosed in 2012 (25% of all female cancer cases and 12% of the total) [1]. Although advances in screening, surgical intervention, radiotherapy and systemic therapies have improved survival, worldwide approximately 522,000 women died as a result of their breast cancer in 2012 [2].

Breast cancer is a heterogeneous disease and there are many ways to classify it, including using traditional histopathological features, immunohistological and molecular classifications [3]. Around 75% of breast cancers are defined and driven by Oestrogen receptor alpha (ERα) transcriptional activity. A number of established endocrine treatments already exist, including Selective Oestrogen Receptor Modulators (SERMs) such as tamoxifen, Selective Oestrogen Receptor Downregulators (SERDs) such as fulvestrant, and Aromatase inhibitors (AIs) such as Letrozole, anastrozole (both non-steroidal) and exemestane (steroidal). However, clinical outcomes vary considerably, and a proportion of women with early breast cancer driven by ERα transcriptional activity develop drug resistance, and relapse with incurable, metastatic disease. There is an urgent need for better treatment strategies.

7 Rationale for Trial

7.1 Data from non-clinical trials

It has been known for many years that Oestrogen receptor (ER) directly induces expression of the Progesterone Receptor (PR) in normal and malignant breast epithelial cells, so that these steroid receptors commonly co-localise. Historically, the accepted explanation was that PR-positivity was a passive consequence of a functional ER, and PR was established as a biomarker of ER functionality in breast cancer [4]. However in recent years, the Carroll laboratory (Cambridge Institute, Cancer Research UK) has published preclinical findings exploring ERfunction in breast cancer, providing new insights into progestogen action and functional ‘cross-talk’ between ER and PR in breast cancer [5].

These preclinical discoveries have provided an alternative explanation to the previous over-simplistic assumption that PR is just a passive consequence of an active ER. In fact, PR can be the cause and not the consequence of altered ER activity.

In the presence of agonist ligands, progesterone-activated PR causes rapid redistribution or sequestration of ERα chromatin binding sites in breast cancer cells, resulting in a unique gene expression program that is associated with a good clinical outcome, via inhibition of ERα-mediated transcriptional activity and the oestrogen-mediated proliferative response (Figure 2). Thus, PR functions as a molecular rheostat to control ERα chromatin binding and transcriptional activity, with important implications for prognosis and therapeutic interventions.

This functional ‘cross-talk’ between ERα and PR was explored preclinically by the demonstration that progesterone inhibited oestrogen-mediated growth in primary ERα+ breast tumour explants and ERα+ cell line xenografts [5]. In both MCF-7 and T-47D xenograft models, progesterone alone antagonized oestrogen-induced tumour

formation, as did tamoxifen alone. However, the combination of tamoxifen and progesterone had the greatest tumour inhibitory effect.

The potential clinical significance of exploiting this interaction between ER and PR signaling in breast cancer affords the possibility that the addition of a progesterone agonist might enhance the anti-proliferative effect of anti-oestrogen therapies and therefore prove a more effective combination therapy.

Figure 2. [6]

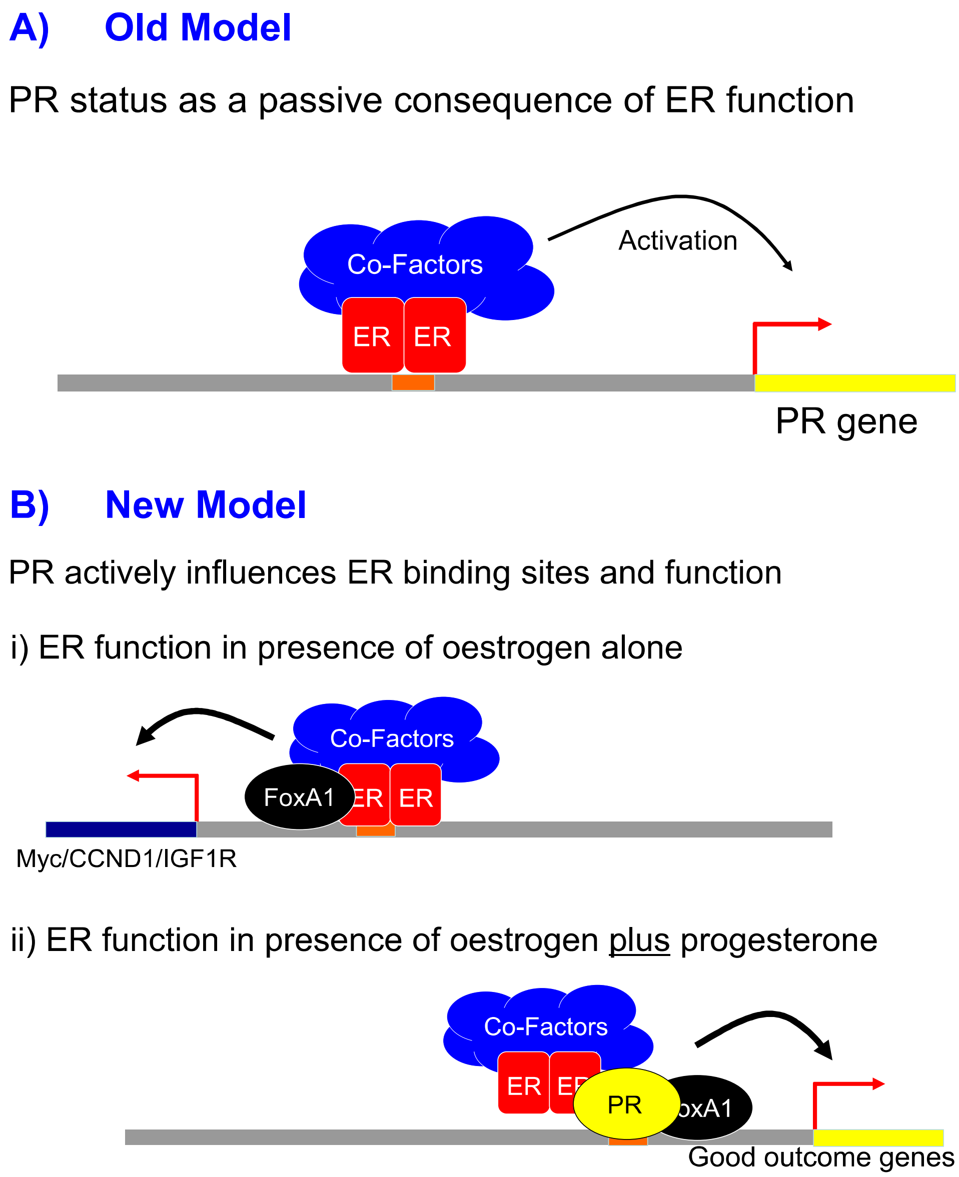

7.2 Clinical Data

7.2.1 Efficacy and tolerability

A wealth of data already exists confirming the clinical benefit of the addition of Megestrol acetate, a semi-synthetic derivative of progesterone, as a single agent in ER+ breast cancer. Overall, 9 trials have reported survival benefit from treatment with Megestrol acetate in ER+ metastatic breast cancer. Older trials have demonstrated that the benefits of Megestrol acetate are comparable to tamoxifen as first line therapy [7-9]. More recently, Megestrol acetate was found to be efficacious in patients with ER-positive metastatic breast cancer after Aromatase Inhibitor (AI) treatment failure [10]. In this single arm Phase II trial, 48 postmenopausal women were treated with Megestrol acetate 160mg daily. The treatment was well-tolerated, and yielded a clinical benefit rate of 40%, with a median duration of 10 months.

Combination treatment with Megestrol acetate and tamoxifen has previously been found to be a tolerable combination in a randomised comparison of the effects of tamoxifen, Megestrol acetate, or tamoxifen plus Megestrol acetate (11). The combined treatment did not result in a higher response rate, however tamoxifen, as opposed to an AI, was used in a post-menopausal population with metastatic (not early) breast cancer, in a trial that was halted prematurely due to poor patient accrual.

A primary rationale for conducting PIONEER, in addition to seeking an increased anti-proliferative effect, is to help decide whether or not there is value in conducting a larger follow-on adjuvant trial investigating the longer term benefit of the combination of an AI +/- Megestrol acetate, and if so, at what dose of Megestrol Acetate (40mg vs. 160mg).

A second important rationale for a larger adjuvant trial of combination therapy, is to potentially improve the quality of life of women taking anti-oestrogens, at a time in which the intensity [12] and duration [13] of adjuvant endocrine therapy is increasing. The use of low dose progesterone as a supportive therapy has been shown to completely and rapidly ameliorate anti-oestrogen therapy-related hot flushes in 75-85% of patients [14]. This could hopefully prevent patients from prematurely ceasing their adjuvant endocrine therapy thereby improving clinical outcomes.

The efficacy of Letrozole as a single agent has been well established, supporting its current registered use in the following settings:

- Adjuvant and Neoadjuvant treatment of postmenopausal women with hormone receptor positive early breast cancer
- Extended Adjuvant treatment of Early Breast Cancer
- First and Second-Line Treatment of Advanced Breast Cancer

7.2.2 Concept of short-term pre-operative treatment

Short-term preoperative ‘window’ trials of 2-4 weeks treatment are a validated strategy offering rapid and cost-efficient proof-of-concept for novel treatment approaches by assessing the direct effects of the trial treatment on tumour tissue. These trials allow access to tumour tissue before and after treatment, providing critical insight into differences in activity and mechanisms between agents, the optimal patient population,

influence of the tumour biology on sensitivity, and molecular mechanisms of response or resistance.

Multiple trials in the neoadjuvant setting have demonstrated the utility and validity of changes in Ki67 as a predictor of benefit from treatment and of long-term outcome. In the neoadjuvant IMPACT trial, suppression of Ki67 at 2 weeks was greater with anastrozole than with either tamoxifen or the combination of anastrozole plus tamoxifen [15-17], mirroring the results of the much larger adjuvant ATAC trial without the requirement of a long follow-up [18].

Although Ki67 measurements in preoperative trials cannot replace the need for adjuvant trials with clinical endpoints, they can be highly instructive in selecting or rejecting candidate approaches for phase III trials, and defining the most appropriate patient populations. Over recent years, the perioperative window setting of this trial together with the incorporation of primary biological endpoints has been established in the UK as a new approach for breast cancer research. The POETIC trial, a UK NCRN phase III randomised clinical trial with approximately 4000 patients, is currently testing prospectively whether short-term perioperative endocrine therapy with an AI followed by standard adjuvant therapy can improve outcome in postmenopausal women with ER-positive breast cancer. It is also testing whether the proliferation marker Ki67, as measured by immunohistochemistry (IHC) after 2 weeks of AItherapy, will predict for relapse free survival and whether molecular profiling 2 weeks after starting endocrine therapy predicts better for long-term outcome than at diagnosis.

8 Trial Design

8.1 Statement of design

This is a three arm, open-label, multicentre, randomized, window of opportunity, phase II trial which will evaluate the effects of 15 days (+ ≤4 days) preoperative therapy with Letrozole, or Letrozole plus low dose Megestrol acetate (40mg), or Letrozole plus high dose Megestrol acetate (160mg) in postmenopausal women with newly diagnosed, ER-positive, HER2-negative, invasive primary breast cancer of at least 1 cm size.

8.2 Number of Centres

Patients will be recruited from surgical or oncology clinics with approximately 15 participating UK centres.

8.3 Number of Subjects

A total of 189 evaluable patients are required.
Evaluable patients are defined as eligible patients who have completed at least 13 (or 80%) of the full 15 Day (+≤4 Days) trial dosing schedule. It is anticipated this will require approximately 10% more patients to be randomised, to account for patients that may not be evaluable.

8.4 Subjects Trial duration

The estimated trial duration for each participant is approximately 8 weeks from first contact, including a 15 Day (+≤4 Days) treatment duration and a 2-week post-treatment follow-up visit.

8.5 Trial Objectives

8.5.1 Primary Objective

- To determine if the addition of Megestrol Acetate increases the anti-proliferative effect of Letrozole when given for 15 days pre-operatively in patients with early-stage, ER-positive breast cancer, as measured by change in Ki67.

8.5.2 Secondary Objectives

- To compare and correlate the biological effects of Letrozole alone compared to Letrozole plus Megestrol Acetate using other immunohistochemical markers of tumour response: Caspase 3, Aurora kinase A, change in expression of the androgen and progesterone receptors, and the absolute value of Ki67 at Day 15
- To compare and correlate the change in Ki67 and the biological effects of low dose Megestrol Acetate compared to high dose Megestrol Acetate using other immunohistochemical markers of tumour response: Caspase 3, Aurora kinase A, change in expression of the androgen and progesterone receptors, and the absolute value of Ki67 at Day 15
- To assess the safety and tolerability of the combination of Letrozole +/- Megestrol Acetate by recording and assessing adverse and serious adverse events.

8.5.3 Exploratory objectives

- To assess progestin-induced ER reprogramming following treatment with Letrozole and Megestrol Acetate, using transcription factor mapping (ChIP-seq) of ER, and RNA sequencing (Addenbrooke's patients only).
- To compare and correlate the biological effects of Letrozole alone compared to Letrozole plus Megestrol Acetate and also the effects of low dose Megestrol Acetate compared to high dose Megestrol Acetate using epithelial mesenchymal transition markers.
- To correlate efficacy and exploratory findings of this trial with breast cancer genomic profiling datasets [22].

Optional Imaging sub-study (Addenbrooke's patients only): to noninvasively assess the anti-proliferative effect of Letrozole vs. Letrozole + Megestrol Acetate using ^{18}-FLT-PET/MRI imaging indices, and to correlate this with the changes observed in Ki67. (Please see Appendix 4 ^{18}F-FLT PET/MR Imaging Sub-Study for further details).

8.6 Trial Endpoints

8.6.1 Primary Endpoint

Change in tumour proliferation measured by Ki67 immuno-histochemical (IHC) assessment (%) between baseline and day 15 (+≤4 Days).

8.6.2 Secondary endpoints:

Biological Endpoints

- Change in tumour apoptosis between baseline and Day 15 (+≤4 Days), measured by Caspase 3 (IHC)
- Change in expression of androgen receptor and PR (IHC) between baseline and Day 15 (+≤4 Days)
- Change in proliferation by Aurora Kinase A (IHC) between baseline and Day 15 (+≤4 Days)
- Absolute value of Ki67 at Day 15 (+≤4 Days)

Safety Endpoints

- Incidence of Serious Adverse Events (SAEs)
- Incidence of Adverse Events (AEs) of all grades (CTCAE Version 4.03)

8.6.3 Exploratory Endpoints

Other translational research questions will be addressed:

- Transcription factor mapping (ChIP-seq) of ER
- Gene expression changes associated with treatment, measured by RNA-sequencing
- Change in epithelial mesenchymal transition markers (IHC) between baseline and Day 15 (+≤4 Days)
- Correlate differences in response to treatments with breast cancer genomic profiling datasets
- Changes in tumour proliferation measured by ^{18}FLT-PET/MRI (optional imaging sub-study only) and correlate these changes with changes observed in Ki67.

9 Selection and withdrawal of subjects

9.1 Inclusion Criteria

To be included in the trial the patient must satisfy all of the following:

- Histologically confirmed breast adenocarcinoma
- Postmenopausal women, defined as having experienced at least one of the following:
  - 12 months of natural (spontaneous) amenorrhea with an appropriate clinical profile (e.g. ≥50 years, history of vasomotor symptoms) or
  - six months of spontaneous amenorrhea with serum FSH and oestradiol levels consistent with postmenopause or
  - surgical bilateral oophorectomy (with or without hysterectomy) at least six weeks ago.
- Core biopsy confirmation of invasive carcinoma on core biopsy, ≥T1c, either clinical NX or N0-N3
- ER positive (Allred≥3) and HER2 negative
- 2 groups of patients are potentially eligible:
  - Cohort A: Patients whose cancers have been deemed to be operable by the Multi-Disciplinary Team (MDT) with tumour excision planned for the next 2-6 weeks
  - Cohort B: Patients with early or locoregionally advanced breast cancer planned for primary endocrine therapy either in lieu of tumour excision or as neoadjuvant therapy before tumour excision – such patients must begin PIONEER trial therapy prior to starting any other endocrine therapy.
- ECOG performance status of 0, 1 or 2
- Adequate Liver, Renal and Bone marrow function, defined as:
  - Adequate liver function where bilirubin is ≤1.5 x ULN
  - Adequate renal function with a serum creatinine ≤ 1.5 x ULN
  - Adequate bone marrow function with ANC ≥1.0 x 10^9/L and Platelet count ≥100 x 10^9/L
- Written informed consent (IC) to participate in the trial and to donation of tissue

9.2 Exclusion Criteria

The presence of any of the following will preclude patient inclusion:

- History of hormone replacement therapy in the last 6 months
- Previous treatment with tamoxifen or an aromatase inhibitor in the last 6 months
- Known hypersensitivity or contraindications to Aromatase Inhibitors (AIs) or Megestrol acetate
- Known allergy to lactose
- Known to have a progestogen-containing intrauterine system in situ, unless removed prior to randomisation
- Known metastatic disease on presentation
- Recurrent breast cancer (patients with a new primary invasive breast cancer will be eligible to participate)
- Serious concomitant disorders that would compromise the safety of the patient or compromise the patient's ability to complete the trial, at the discretion of the investigator
- Treatment with an investigational drug within 4 weeks before randomisation
- Inability to swallow orally administered medication and patients with gastrointestinal disorders likely to interfere with absorption of the trial medication
- Inability to give informed consent (IC)

9.3 Treatment Assignment and Randomisation Number

Eligible patients will be randomly assigned, using a minimization method with a random element, to either the control arm (Letrozole alone) or one of the two research arms (Letrozole with Megestrol Acetate at low and high dose) in a 1:1.5:1.5 ratio.

The stratification factors are:

- tumour grade (1,2,3),
- ductal vs. lobular subtype,
- intensity of ER staining (3-6, 7-8), reported on the initial diagnostic core biopsy, to ensure that each arm of the trial is well balanced.

Randomisation must be complete prior to the start of trial treatment.

Randomisation will be implemented using a web-based central randomisation system by the relevant participating site staff. The web-based central randomisation system will allocate patient treatment arm and trial numbers sequentially in the order in which the patients are randomised.

At the site initiation, the trial coordinator will train site staff in how to access and use the randomisation system.

This is an open-label trial. The histopathologist assessing the biological response (primary endpoint, Ki67 expression by IHC; secondary endpoint, Caspase-3 by IHC) will, however, be blinded as to treatment allocation.

9.4 Discontinuation of trial treatment and patient withdrawal

Discontinuation of trial treatment or withdrawal of consent by patients for any reason should be communicated to the PIONEER office as soon as possible by telephone, fax or email.

9.4.1 Discontinuation

Patients should discontinue trial treatment in the following circumstances and this should be reported in the End of Trial Medications Case Report Form (CRF):

1. If the Investigator decides that the patient should be discontinued from the treatment at any point due to an inter-current illness or if continued participation in the trial will result in delay to planned tumour excision.

2. Any CTCAE Grade ≥ 3 drug related toxicity which does not resolve to Grade 1 or less within 72 hours (except where supportive measures exist to control the toxicity which had not been adequately instigated at the time of the AE, e.g. antiemetics for nausea and vomiting or anti-motility agents for diarrhoea).

3. If the patient withdraws from the trial, refuses baseline core biopsy (only applies when diagnostic tissue is not available), opts to discontinue the treatment or chooses not to comply with trial procedures.

4. If the patient's disease is progressing while on trial treatment.

Patients who have been discontinued from the trial treatment and are experiencing ongoing toxicity from Letrozole and/or Megestrol acetate will be followed up as clinically indicated until the toxicity resolves to Grade 1, or less. Patients who have completed the minimum 13 days of treatment (or ≥ 80% days of dosing if 16 to 19 days of treatment) prior to developing toxicity will continue to attend scheduled follow ups and procedures (unless decided otherwise by the clinician) and their samples/data will be used for analysis.

However, patients who do not complete at least 13 days of the full 15 day pre-operative window dosing schedule (or ≥ 80% days of dosing if 16 to 19 days of treatment) won't be considered evaluable for the primary endpoint. For patients whose treatment is extended beyond 15 days (up to 19 days), the minimum number of days in which the patient should dose with trial treatment must be greater than 80% of the full treatment window e.g. for 19 days of treatment a minimum of 15 days of study drug must be taken. These patients' samples and data will be retained and may be used for analysis of the secondary and exploratory endpoints. Patients whose treatment is scheduled for 13 days must complete the full treatment window to be evaluable for the primary endpoint (i.e. no treatment doses can be missed).

9.4.2 Withdrawal of consent

Patients may withdraw their consent to participate in the trial at any time. If the patient explicitly states their wish to withdraw from receiving any further trial–specific treatment, the Investigator should inform the PIONEER office promptly and complete the Withdrawal Form. If the patient withdraws consent for participation in the trial, then no further trial procedures will be undertaken and no data or samples will be collected from the time of withdrawal
However, data and samples collected up to the time of consent withdrawal will be included in the data reported for the trial.

10 Procedures and assessments

Patient recruitment, consent and screening will occur within the oncology or surgical unit, depending on the participating site.

Patients will be identified as potentially eligible for participation at a breast cancer surgical MDT meeting, and approached for discussion after being informed of the results of their diagnostic core biopsy confirming ER-positive breast cancer. This initial discussion may occur with the Principal Investigator (PI) or a qualified member of the research team.
If patients are agreeable to participation in the trial they will be provided with a Participant Information Sheet (PIS) and another appointment will be booked by the trial team to discuss participation further and to undertake written Informed Consent (IC) if required.
Once written IC for all study procedures and treatments has been obtained, the participants will undergo screening assessments, including blood tests.
Eligible patients will be randomised to a trial treatment (eligibility confirmation required from central coordinating office). A research tissue from the diagnostic tissue block (up to 2 months before patient starting treatment) will be required. If patient does not have available tissue from diagnosis, they will undergo a baseline research biopsy. Baseline research biopsy may occur before or after randomisation but must be after central coordinating office confirmation.

Trial treatment will then begin on Day 1, patient treatment compliance and an assessment of AE’s will be checked by telephone on Day 5 (+/- 1) and Day 10 (+/- 1), and then the patient will undergo tumour excision and/or a core biopsy on Day 15 (+ ≤4 days). Patients will return for a post-treatment follow-up visit/ phone call on Day 28 (+/- 5 days), two weeks after tumour excision and/or core biopsy. Treatment can be planned for 13-14 days in the exceptional circumstance that tumour excision or Day 15 biopsy can not be scheduled on Day 15 (+ ≤4 days).

Patients in Cohort A will follow standard post-operative procedures as per local policy and SOPs. If standard local practice includes a two week post-operative visit, this two week visit will be arranged on the same day (where possible).

SAEs should be reported from the point of IC, so safety/AE/SAE review should be included during every visit.

10.1 Screening evaluation

10.1.1 Screening Assessments (Day -28 to Day 1)

Trial specific assessments will only be conducted after patients have given written IC. This includes:

a) Medical history
b) Concomitant medication
c) ECOG performance score
d) Full blood count
e) Biochemical series
f) Assessment of menopausal status if it is not clinically evident (confirmed with LH, FSH and Oestradiol)
g) AEs/SAEs (from time of consent)

Results from blood tests within 6-8 weeks prior to informed consent can be used in place of repeated blood results, unless repeat bloods tests are felt to be clinically indicated at the investigator’s discretion.

10.1.2 Subject Registration/Randomisation

Registration and randomisation must be complete prior to the start of trial treatment. Patients will be registered following confirmation of eligibility (including results of screening assessments). Local site staff will complete the 'patient details', 'informed consent' (CRF not original consent form) and 'eligibility' CRFs and forward them to the central coordinating office to register the patient and provide authorisation to randomise. Appropriate staff at the local site will then randomise the patient upon confirmation of central approval. Data will be entered into a web-based central randomisation system (Sealed Envelope) by the relevant local site staff. The web-based central randomisation system will allocate patient trial numbers sequentially in the order in which the patients are randomised.

Supporting documentation required for patient randomisation will include results of an initial diagnostic core biopsy, specifically delineating ER status, histological subtype (ductal or lobular) and tumour grade, in addition to the patient details, IC and eligibility forms. These must be medically reviewed to confirm randomisation. Any queries must be resolved prior to randomisation. The PI or co-investigator will sign the eligibility CRF to confirm eligibility.

10.2 Baseline assessments – Prior to Treatment (Day -15 to Day 1)

Baseline assessments may be performed on the same day as screening provided patient eligibility has been confirmed. For patients in Cohort A, the date of tumour excision must be planned carefully to be performed 15 days (+ ≤4 days) from the start of the trial treatment.
The following data points are to be recorded:

a) Baseline symptoms (non treatment-related, disease-related)
b) Weight in kg and height in cm
c) Concomitant medications
d) AEs/SAEs
e) Baseline research core biopsy of breast tumour (before or after randomisation) if patient does not have available research tissue from the diagnosis (see section 16).
f) Trial treatment may be dispensed (up to Day 1 of trial involvement)

Please refer to Section 16 for more information regarding the **Storage and Analysis of Samples.**

10.3 Trial assessments during protocol treatment

10.3.1 Timing of assessments

10.3.2 After the baseline review, the trial treatment will commence on Day 1. Patient treatment compliance and an assessment of AE's will be checked by telephone on Day 5 (+/- 1) and Day 10 (+/- 1), and then the patient will undergo review and tumour excision and/or core biopsy on Day 15 (+ ≤4 days). In exceptional circumstances tumour excision and/or core biopsy can be scheduled on Day 13-14. Assessments at time points

Day 1:
- Trial treatment to commence.

Day 5 (+/- 1):

- Patient treatment compliance check by telephone
- Telephone call to assess AEs/SAEs
- Concomitant medications

Day 10 (+/- 1):
- Patient treatment compliance check by telephone
- Telephone call to assess AEs/SAEs
- Concomitant medications

Day 15 (+ ≤4 days):
- Last dose of trial drugs
- Return of unused trial drugs
- Review to assess AEs/SAEs
- Tumour excision (for patients in Cohort A)
- Tissue collection (research core biopsy)

10.3.3 Day 15 core biopsy

The effects of the trial treatment will be assessed on tumour tissue specimens taken at baseline and after 15 days of the trial treatment.

For patients in Cohort A (pre-operative patients)

Patients will undergo breast cancer tumour excision (e.g. Lumpectomy or mastectomy +/- lymph node dissection) as determined by the local MDT, in accordance with both local protocols and the patient's consent. Tumour excision should be performed 15 days (+ ≤4 days) from the start of the trial treatment. Trial treatment should be continued until tumour excision or biopsy.

The biopsy should be taken during breast tumour excision provided this is performed on Day 15 (+ ≤4 days). If for logistical reasons, it is not possible to take the biopsy during the tumour excision OR if the patient does not have breast tumour excisionscheduled on Day 15 (+ ≤4 days), then the patient will be asked to undergo a core biopsy on Day 15 (+ ≤4 days) to assess the effect of the trial treatment and can continue treatment with Letrozole alone (regardless of randomised arm) until tumour excision. These patients will continue with the study schedule and complete the study two weeks after their rescheduled date of tumour excision at the end of trial visit.

Patients in Cohort B of the trial
(primary endocrine therapy - either instead of tumour excision ***or as neoadjuvant therapy before*** tumour excision***-)***

Patients will be asked to undergo a core biopsy on Day 15 (+ ≤4 days) to assess the effect of the trial treatment. Subsequently they will start standard of care (i.e. off-trial) endocrine therapy after the "Day 15" research biopsy. This standard of care endocrine therapy will be determined by their treating physician.

10.3.4 Assessments at the Post-Treatment Follow-Up Visit (May be substituted for telephone call)

Day 28 (+/- 5 days)
- ECOG performance status and weight (if visit performed on site)
- Review to assess AEs/SAEs

- Concomitant medications

10.4 Long-Term Follow-up Assessments

Patients will not undergo any planned, long term follow up after their end of trial visit (which may be substituted for telephone call).

10.5 End of Trial Participation

The patient's trial participation ends following completion of their post-treatment follow-up visit or withdrawal of consent for the trial. Patients will be expected to return to their normal, standard pathways of care and follow up after the post-treatment follow-up visit.

10.5.1 Adjuvant endocrine therapy

Following the end of the patient's participation in the trial they will be treated in accordance with local policy based on the national clinical guidelines that prevail at the time. It is expected that patients in Cohort A will receive adjuvant endocrine therapy with either tamoxifen or an AI, or each sequentially, for a minimum of 5 years. It is recognised that choice and duration of endocrine therapy is a changing field and national policies may change during the course of the trial.

10.5.2 Chemotherapy, Radiotherapy, and additional Treatments

Subsequent chemotherapy, radiotherapy, or additional local or systemic therapies after discontinuation or completion of trial therapy will be left to the discretion of the treating clinician/ MDT. PIONEER patients may be offered further clinical trials if they are eligible.

10.6 **Schedule of Assessments**

**DAY OF TRIAL**	Screening (Day -28 to Day 1)	Baseline visit (Day -15 to Day 1)	Day 1 - no contact necessitated (unless screening on D1)	Day 5 +/- 1 (Phone call)[6]	Day 10 +/- 1 (Phone call)[6]	Day 11 (+/- 1)	Day 15 +≤4 Tumour excision/ Core biopsy	Post-Treatment Follow-Up Visit/phone call (Day 28 +/-5 days)
	Visit may occur same day – eligibility must be confirmed prior to baseline visit (see 10.2).							
Written informed consent	X							
Confirmation of Eligibility	X							
Registration and Randomisation	X							
Complete medical history	X							
ECOG performance status [1]	X							X
AEs/SAEs	X	X		X	X		X	X
Haematology and biochemistry[1]	X							
Blood tests for menopause (LH, FSH, oestradiol) [1] [3]	X							
Baseline symptoms (non treatment-related, disease-related)		X						
Height and Weight		X						X[2]
Tissue collection (research core biopsy)		X[5]					X[7]	
Dispense Letrozole (+/- Megestrol acetate		X						
Dosing Letrozole (+/- Megestrol acetate)			Day 1 – Day 15+≤4					
Telephone treatment compliance check				X			X	
Tumour excision[4]							X[7]	
Concomitant medications	X	X		X			X	X
PET/MRI scan (optional sub-study for Cambridge patients only)		X					X	

[1]- Results from blood test within 6-8 weeks before Informed consent can be used in place of repeated blood results, unless repeat bloods tests are felt to be clinically indicated at the investigator's discretion [2]- Weight only will be assessed if visit performed in person, will not be assessed by telephone. Height only assessed at Baseline visit. [3]- Only if menopausal status is not clinically determined. [4]- Only patients in Cohort A will undergo tumour excision. [5]- Baseline research core biopsies of breast tumour (before or after randomisation) if patient does not have available research tissue from diagnostic tissue blocks (see section 16). [6]- Visits that fall during Bank holidays +/-2 days window is allowed. [7]- In exceptional circumstances tumour excision/core biopsy can be scheduled on Day 13-14.

11 Trial Treatments

For the purpose of this trial, Letrozole and Megestrol acetate are both considered as Investigational Medicinal Products (IMPs) conducted within a Clinical Trial Authorisation (CTA).

11.1 Treatment summary

Patients will be stratified by histological grade, ductal vs. lobular subtype and intensity of ER IHC staining, and randomised 1:1.5:1.5 in favour of the 2 combination arms of Letrozole + Megestrol acetate. The trial is powered to confirm our preclinical findings that the combination of Letrozole and Megestrol acetate will have a greater anti-proliferative effect than Letrozole alone [5].

Eligible patients will be randomized to treatment with:

Arm A (Control): 15 days of treatment with Letrozole 2.5mg daily

Arm B (Research Arm 1): 15 days of treatment with Letrozole 2.5mg daily + Megestrol acetate 40mg daily

Arm C (Research Arm 2): 15 days of treatment with Letrozole 2.5mg daily + Megestrol acetate 160mg daily

11.2 Maximum duration of treatment of a patient

Treatment in all three arms should be continued until tumour excision and/or core biopsy on day 15 (+ ≤4 days) unless there is evidence of unacceptable toxicity, progression or the patient requests to discontinue. Treatment can be planned for 13-14 days if exceptional circumstances mean that tumour excision or Day 15 biopsy can not be scheduled on Day 15 (+ ≤4 days).

In exceptional circumstances where tumour excision is delayed beyond the planned 15 day treatment period, we allow treatment to be continued for up to an additional 4 days.

Should patients experience significant AEs that could potentially impact on the operability, Megestrol acetate should be discontinued permanently whereas Letrozole should be continued until breast tumour excision is performed. If breast tumour excisioncannot be performed on day 15 (+ ≤4 days) patients will be required to undergo an additional core biopsy on day 15 (+ ≤4 days) to assess the effect of the trial treatment.

Following the 19 days of study treatment, in the event of surgical delay beyond this time, patients may choose to continue treatment with Letrozole alone until the day of tumour excision.

Patients will be excluded from the final analysis if they do not complete at least 13 of the full 15 day pre-operative window dosing schedule, necessitating recruitment of an additional patient.

11.3 Procedures for monitoring treatment compliance

Letrozole and Megestrol acetate are planned as a self-administered outpatient treatment. Daily dosing should be recorded by the patient in their patient diary. The patient should be encouraged to take the required doses according to the treatment plan. Any omissions should be reported to the investigator or the trials nurse and recorded in the patient diary and in the CRF by the site staff together with the reason for the omission. Any dispensed but unused drug at the end of the treatment period should be counted and noted on the CRF to ensure the minimum requirement was achieved.

11.4 Supply, accountability and dispensing

11.4.1 Pharmacy responsibilities

All pharmacy aspects of the trial at the participating sites are the responsibility of the PI who will delegate this responsibility to the local pharmacist, or other appropriately qualified personnel. This delegation of duties must be recorded on the Site Staff Delegation Log. The PI or a delegated individual, e.g. the trial pharmacist, must ensure that the trial medications are stored and dispensed in accordance with local practice, applicable regulatory requirements and trial-specific prescriptions.

11.4.2 Drug accountability

The site pharmacy must maintain accountability records for all of the IMPs which may include receipt, dispensing, returned medication, storage conditions and destruction of returned/unused medication. Template accountability forms will be supplied however, sites are permitted to use their own drug accountability records as long as the same information is recorded and is available to the Sponsor. Copies of completed drug accountability logs must be submitted for all trial patients upon request from the PIONEER office for monitoring purposes.

Following the termination of the trial and at the request of the Sponsor, all unused IMPs will be accounted for and destroyed locally at the trial sites in accordance with local practice. See the latest version of the pharmacy manual for details.

11.4.3 Dispensing

Letrozole, Megestrol acetate 40mg and Megestrol acetate 160mg will be dispensed by the pharmacy at the participating site in accordance with a trial specific prescription. Sites are permitted to use their own clinical trial template prescription. Prior approval of the final prescription template by the Sponsor is required.
See pharmacy manual for further details.

11.5 Investigational Medicinal Products

11.6 Letrozole

11.6.1 Legal status

The Investigational Medicinal Product (IMP) Letrozole is currently licenced in the UK for the treatment of postmenopausal women with hormone receptor positive breast cancer. Letrozole is off-patent and has generic availability. It has registered indications in the neoadjuvant, adjuvant and advanced breast cancer settings.

Description: Letrozole is an oral non-steroidal AI for the treatment of hormonally-responsive breast cancer

Supply: Commercial Letrozole tablets will be obtained locally by the investigating sites in keeping with standard local practice. Its handling and management will be subject to standard procedures of the pharmacy.

11.6.2 Dose and Route and Administration

Patient will self-administer Letrozole by mouth. Letrozole 2.5mg tablets to be taken ONCE a day for 15 (+ ≤4) days prior to tumour excision.
It can be taken with or without food. There is no specific brand of Letrozole that must be used for this trial. Sites must ensure that the active substance used is Letrozole.

If vomiting occurs shortly after the Letrozole tablet is swallowed, the dose should only be replaced if all of the intact tablet can be seen. Should any patient miss a scheduled dose for whatever reason (e.g. as a result of forgetting to take the tablets), the patient will be allowed to take the scheduled dose up to a maximum of 12 hours after that scheduled dose time. If greater than 12 hours after the scheduled dose time, the missed dose is not to be taken and the patient should take their next dose at the next scheduled time.

11.6.3 Labelling

Letrozole will be labeled according to Eudralex Volume 4: Annex 13 Investigational Medicinal Products of the EU guide to Good Manufacturing Practice (GMP) by the participating sites. An example of the label can be found in the Pharmacy Manual.

11.6.4 Storage Conditions

Letrozole must be stored, handled and dispensed as detailed in the current Summary of Product Characteristics (SmPC) of the brand being used.

11.6.5 Returns and destructions

All previously dispensed, unused Letrozole should be destroyed once the amount of remaining tablets has been counted and recorded in the relevant accountability forms. Refer to the pharmacy manual.

11.6.6 Adverse Events

The most common Adverse Events (AEs): Vascular disorders, flushing, cerebrovascular accident, hypertension, thromboembolic event, angina, skin rash, increased sweating, increased hepatic enzymes and arthralgia/arthritis.
See package insert for complete list of adverse effects and the current SmPC for the brand being used.

11.6.7 Contraindications

Avoid co-administration of Letrozole with tamoxifen, other anti-oestrogens or oestrogen-containing therapies as these substances may diminish the pharmacological action of Letrozole

11.7 Megestrol acetate 40mg tablets

11.7.1 Legal status

Megestrol acetate 40mg is not licenced in the UK but is available in the USA.
This trial is being carried out under a CTA. Megestrol 40mg is therefore only to be used by the named Investigators, for the patients specified in this protocol, within the trial.

Description: Megestrol acetate is a potent progestogen that exerts significant anti-oestrogenic effects. It has no androgenic or oestrogenic properties. It has anti-gonadotropic, anti-uterotropic and anti-androgenic/anti-myotropic actions. It has a slight but significant glucocorticoid effect and a very slight mineralocorticoid effect.

Supply: Megestrol 40mg will be supplied free of charge to participating sites. Megestrol 40mg will be labelled and distributed by Mawdsley-Brooks & Co Ltd in accordance with local regulations and Good Manufacturing Practice.
Megestrol 40mg tablets will be distributed directly to site following confirmation that all necessary regulatory and ethical approvals are in place.
Further details on the ordering of supplies and dispensing procedures are provided in the pharmacy manual.

11.7.2 Dose, Route and Administration

Patient will self-administer Megestrol 40mg by mouth. Patients in Arm B will take Megestrol 40mg tablets to be taken ONCE a day for 15 (+ ≤4) days prior to tumour excision. It can be taken with or without food.

If vomiting occurs shortly after the Megestrol tablet is swallowed, the dose should only be replaced if all of the intact tablet can be seen. Should any patient miss a scheduled dose for whatever reason (e.g. as a result of forgetting to take the tablets), the patient will be allowed to take the scheduled dose up to a maximum of 12 hours after that scheduled dose time. If greater than 12 hours after the scheduled dose time, the missed dose is not to be taken and the patient should take their next dose at the next scheduled time.

11.7.3 Storage conditions

Megestrol 40mg tablets is stored at room temperature (25°C), excursions are permitted to 15-30°C, and kept in the original package in order to protect from moisture or as per instructions on the label.

11.7.4 Labelling

Megestrol 40mg tablets will be provided to participating sites already labeled according to Eudralex Volume 4: Annex 13 Investigational Medicinal Products of the EU guide to GMP. Sample of the label can be found in the Pharmacy Manual.

11.7.5 Returns and destructions

All previously dispensed, unused Megestrol acetate 40mg should be destroyed once the amount of remaining tablets has been counted and recorded in the relevant

accountability forms. Refer to the pharmacy manual.

11.7.6 Adverse Events (AEs)

The most common Adverse Events (AEs):
Weight gain, thromboembolic phenomena, glucocorticoid effects, heart failure, nausea and vomiting, edema, breakthrough menstrual bleeding, dyspnea, tumor flare (with or without hypercalcemia), hyperglycemia, glucose intolerance, alopecia, hypertension, carpal tunnel syndrome, mood changes, hot flashes, malaise, asthenia, lethargy, sweating and rash.
See the current SIMPD for complete list of adverse effects.

11.7.7 Contraindications

History of hypersensitivity to Megestrol acetate or any component of the formulation.

11.8 Megestrol acetate 160mg tablets

11.8.1 Legal status

Megestrol acetate 160mg daily is currently licenced in the UK for the treatment of hormone-dependant breast cancer.

Description: Megestrol acetate is a potent progestogen that exerts significant anti-oestrogenic effects. It has no androgenic or oestrogenic properties. It has anti-gonadotropic, anti-uterotropic and anti-androgenic/anti-myotropic actions. It has a slight but significant glucocorticoid effect and a very slight mineralocorticoid effect.

Supply: Commercial Megestrol 160mg tablets will be obtained locally by the investigating sites in keeping with standard local practice. Its handling and management will be subject to standard procedures of the pharmacy.

11.8.2 Dose and Route and Administration

Patient will self-administer Megestrol by mouth. Patients in Arm C will take Megestrol 160mg tablets to be taken ONCE a day for 15 (+ ≤4) days prior to tumour excision.
It can be taken with or without food. There is no specific brand of Megestrol 160mg that must be used for this trial. Sites must ensure that the active substance used is Megestrol Acetate.

If vomiting occurs shortly after the Megestrol tablet is swallowed, the dose should only be replaced if all of the intact tablet can be seen. Should any patient miss a scheduled dose for whatever reason (e.g. as a result of forgetting to take the tablets), the patient will be allowed to take the scheduled dose up to a maximum of 12 hours after that scheduled dose time. If greater than 12 hours after the scheduled dose time, the missed dose is not to be taken and the patient should take their next dose at the next scheduled time.

11.8.3 Storage conditions

Megestrol 160mg must be stored, handled and dispensed as detailed in the current SmPC of the brand being used. Megestrol 160mg is stored at room temperature below

25°C, and kept in the original package in order to protect from moisture or as per SmPC for the brand used.

11.8.4 Labelling

Megestrol 160mg tablets will be labeled according to Eudralex Volume 4: Annex 13 Investigational Medicinal Products of the EU guide to GMP at the participating sites. An example of the label can be found in the Pharmacy Manual.

11.8.5 Returns and destructions

All previously dispensed, unused Megestrol acetate 160mg should be destroyed once the amount of remaining tablets has been counted and recorded in the relevant accountability forms. Refer to the pharmacy manual for further details.

11.8.6 Adverse Events (AEs)

The most common Adverse Events (AEs):
Weight gain, thromboembolic phenomena, glucocorticoid effects, heart failure, nausea and vomiting, edema, breakthrough menstrual bleeding, dyspnoea, tumor flare (with or without hypercalcemia), hyperglycemia, glucose intolerance, alopecia, hypertension, carpal tunnel syndrome, mood changes, hot flashes, malaise, asthenia, lethargy, sweating and rash. See package insert for complete list of adverse effects and the current SmPC for the brand being used.

11.8.7 Contraindications

History of hypersensitivity to Megestrol acetate or any component of the formulation.

11.9 IMP delivery

In the event of local guidance advising that hospital visits should be avoided or the participant is unable to collect the medication in person, the trial treatment can be delivered directly to the participant's home from the site by courier. Please see the Pharmacy Manual for more information.

11.10 Dosage modifications

There will be no dose reductions for Letrozole or Megestrol acetate. Letrozole +/- Megestrol acetate may be held or discontinued at the discretion of the treating physician for endocrine-related toxicity, however patients will be excluded from the final analysis if they do not complete at least 13 days of the full 15 day pre-operative window dosing schedule (or ≥ 80% days of dosing if 16 to 19 days of treatment).

11.11 Concomitant therapy

Treatment with concomitant, systemic or investigational anti-cancer agents are not allowed in this trial.

Avoid Co-administration of Letrozole with Tamoxifen, other anti-oestrogens or oestrogen-containing therapies as these substances may diminish the pharmacological action of Letrozole.

Supportive care, including but not limited to anti-emetic medications, may be administered at the discretion of the site PI, as medically indicated.

All concomitant medication must be recorded on the CRF. Standard intra- and peri-operative medications routinely administered at the study site, including anesthesia,

prophylactic antibiotics and anticoagulants, should not be recorded on the CRF. However, these medications should be recorded on the CRF if considered medically relevant by the PI.

12 Assessment of Safety

12.1 Definitions

12.1.1 Adverse Event (AE)

Any untoward medical occurrence in a patient or clinical trial subject administered a medicinal product and which does not necessarily have a causal relationship with this treatment.
An AE can therefore be any unfavourable and unintended sign (including an abnormal laboratory finding), symptom, or disease temporally associated with the use of an investigational medicinal product, whether or not considered related to the investigational medicinal product.

Please note: Recording of all AEs must start from the point of IC regardless of whether a patient has yet received a medicinal product.

12.1.2 Adverse Reaction to an investigational medicinal product (AR)

All untoward and unintended responses to an investigational medicinal product related to any dose administered. All AEs judged by either the reporting investigator or the sponsor as having a reasonable causal relationship to a medicinal product qualify as Adverse Reactions (ARs). The expression reasonable causal relationship means to convey in general that there is evidence or argument to suggest a causal relationship.

12.1.3 Unexpected adverse reaction

An Adverse Reaction (AR), the nature or severity of which is not consistent with the applicable Reference Safety Information (RSI), as evident from the SmPC or SIMPD.

When the outcome of the AR is not consistent with the applicable RSI this AR should be considered as unexpected.

The term "severe" is often used to describe the intensity (severity) of a specific event. This is not the same as "serious," which is based on patient/event outcome or action criteria.

12.1.4 Serious Adverse Event or Serious Adverse Reaction (SAE / SAR)

Any untoward medical occurrence that at any dose:
- results in death
- is life-threatening
- requires hospitalisation or prolongation of existing inpatients hospitalisation,
- results in persistent or significant disability or incapacity
- is a congenital anomaly or birth defect.
- is an important medical event - Some medical events may jeopardise the patient or may require an intervention to prevent one of the above characteristics/ consequences. Such events (hereinafter referred to as 'important medical events') should also be considered as 'serious'.

Life-threatening in the definition of a SAE or SAR refers to an event in which the patient was at risk of death at the time of event; it does not refer to an event which hypothetically might have caused death if it were more severe.

12.1.5 Suspected Unexpected Serious Adverse Reaction (SUSAR)

A Serious Adverse Reaction (SAR), the nature and severity of which is not consistent with the information set out in the RSI.

12.1.6 Reference Safety Information (RSI)

A list of medical events that defines which reactions are expected for the IMP within a given trial and thus determining which SARs require expedited reporting.

The Reference Safety Information (RSI) approved by Medicines and Healthcare products Regulatory Agency (MHRA) for use in this trial is:

- Letrozole: Table 1 of section 4.8 of SmPC for Letrozole 2.5mg film-coated tablets (Accord)
- Megestrol acetate (40mg): "Adverse Reactions" section of sIMPD for Megestrol acetate tablet Par Pharmaceutical Inc.
- Megestrol acetate (160mg): Table in section 4.8 of the SmPC Megace 160 mg tablets (Bausch & Lomb U.K Limited).

12.2 **Expected Adverse Reactions/Serious Adverse Reactions (AR /SARs)**

All expected ARs are listed in the latest MHRA approved version of the reference safety information as specified in section 12.1.6. This must be used when making a determination as to the expectedness of the AR. If the AR meets the criteria for seriousness, this must be reported as per section 12.6.

12.3 **Expected Adverse Events/Serious Adverse Events (AE/SAE)**

All AEs/SAEs should be recorded in the trial documentation and CRF excepting minor side effects of the biopsy such as grade 1 bleeding, bruising or pain, as well as skin reactions to the plaster following biopsy or phlebotomy.

Hospitalisation for scheduled breast tumour excision should not be treated as an AE and as such should not be recorded or reported.

All AEs/SAEs should be recorded in the trial documentation and CRF excepting minor side effects related to standard tumour excision and unrelated to the study drug. These include grade ≤2:

- Bleeding
- Pain
- Bruising
- Infection
- Post-Op Drain Complications

Any incidence of thromboembolism, however, will need to be recorded and reported if the event fulfills the criteria for seriousness as per section 12.1.4.

12.4 Evaluation of adverse events

The Sponsor expects that AEs are recorded from the point of IC regardless of whether a patient has yet received a medicinal product. Individual AEs should be evaluated by the investigator. This includes the evaluation of its seriousness and any relationship between IMP(s) and/or concomitant therapy and the AE (causality).

12.4.1 Assessment of seriousness

Seriousness is assessed against the criteria in section 12.1.4. This defines whether the event is an AE, SAE or a SAR.

12.4.2 Assessment of causality

Definitely: A causal relationship is clinically/biologically certain. **This is therefore an Adverse Reaction**

Probable: A causal relationship is clinically / biologically highly plausible and there is a plausible time sequence between onset of the AE and administration of the investigational medicinal product and there is a reasonable response on withdrawal. **This is therefore an Adverse Reaction.**

Possible: A causal relationship is clinically / biologically plausible and there is a plausible time sequence between onset of the AE and administration of the investigational medicinal product. **This is therefore an Adverse Reaction.**

Unlikely: A causal relation is improbable and another documented cause of the AE is most plausible. **This is therefore an Adverse Event.**

Unrelated: A causal relationship can be definitely excluded and another documented cause of the AE is most plausible. **This is therefore an Adverse Event.**

Unlikely and Unrelated causalities are considered NOT to be trial drug related
Definitely, Probable and Possible causalities are considered to be trial drug related

A pre-existing condition must not be recorded as an AE or reported as an SAE unless the condition worsens during the trial and meets the criteria for reporting or recording in the appropriate section of the CRF.

12.4.3 Clinical assessment of severity

All Adverse Events (AEs) should be graded for severity according to the NCI-CTCAE Toxicity Criteria (Version 4.03). CTCAE v4.03 can be downloaded from the following URL:

http://evs.nci.nih.gov/ftp1/CTCAE/CTCAE_4.03_2010-06-14_QuickReference_8.5x11.pdf

12.4.4 Recording of adverse events

Adverse Events (AEs) and Adverse Reactions (ARs) should be recorded in the medical notes and the appropriate section of the CRF and/or AE/AR log. SAEsand SARs should be reported to the sponsor as detailed in section 12.5.

12.5 Reporting serious adverse events

Each Principal Investigator needs to record all AEs and report SAEs to the Chief Investigator (CI) using the trial specific SAE form within 24 hours of their awareness of the event. The CI is responsible for ensuring the assessment of all SAEs for

expectedness and relatedness is completed and the onward notification of all SAEs to the Sponsor immediately but not more than 24 hours of first notification. The sponsor has to keep detailed records of all SAEs reported to them by the trial team.

The Chief Investigator (CI) is also responsible for prompt reporting of all SAE findings to the competent authority (e.g. MHRA) of each concerned Member State if they could:

- adversely affect the health of patients
- impact on the conduct of the trial
- alter the risk to benefit ratio of the trial
- alter the competent authority's authorisation to continue the trial in accordance with Directive 2001/20/EC.

The completed SAE form can be faxed or emailed. Details of where to report the SAE's can be found on the PIONEER SAE form and the front cover of the protocol.

SAE/SARs should be recorded and reported up to the post-treatment follow-up visit and any SAEs will be followed up until resolved to grade 1.

12.6 Reporting of Suspected Unexpected Serious Adverse Reactions (SUSARs)

All suspected adverse reactions related to an investigational medicinal product (the tested IMP and comparators) which occur in the concerned trial, and that are both unexpected and serious (SUSARs) are subject to expedited reporting. Please see section 12.1.6 for the Reference Safety Information to be used in this trial.

12.6.1 Who should report and whom to report to?

The Sponsor delegates the responsibility of notification of SUSARs to the CI. The CI must report all the relevant safety information previously described, to the:

- Sponsor
- competent authorities in the concerned member states (e.g. MHRA)
- Ethics Committee in the concerned member states

The CI shall inform all investigators concerned of relevant information about SUSARs that could adversely affect the safety of patients.

12.6.2 When to report?

12.6.2.1 Fatal or life-threatening SUSARs

All parties listed in 12.6.1 must be notified as soon as possible but no later than **7 calendar days** after the trial team and Sponsor has first knowledge of the minimum criteria for expedited reporting.
In each case relevant follow-up information should be sought and a report completed as soon as possible. It should be communicated to all parties within an additional **8 calendar days**.

12.6.2.2 Non-fatal and non-life-threatening SUSARs

All other SUSARs and safety issues must be reported to all parties listed in 12.6.1 as soon as possible but no later than **15 calendar days** after first knowledge of the minimum criteria for expedited reporting. Further relevant follow-up information should be given as soon as possible.

12.6.3 How to report?

12.6.3.1 Minimum criteria for initial expedited reporting of SUSARs

Information on the final description and evaluation of an AR report may not be available within the required time frames for reporting. For regulatory purposes, initial expedited reports should be submitted within the time limits as soon as the minimum following criteria are met:
a) a suspected investigational medicinal product,
b) an identifiable patient (e.g. trial patient code number),
c) an adverse event assessed as serious and unexpected, and for which there is a reasonable suspected causal relationship,
d) an identifiable reporting source,

and, when available and applicable:
- an unique clinical trial identification (EudraCT number or in case of non-European Community trials the sponsor's trial protocol code number)
- an unique case identification (i.e. sponsor's case identification number).

12.6.3.2 Follow-up reports of SUSARs

In case of incomplete information at the time of initial reporting, all the appropriate information for an adequate analysis of causality should be actively sought from the reporter or other available sources. Further available relevant information should be reported as follow-up reports.
In certain cases, it may be appropriate to conduct follow-up of the long-term outcome of a particular reaction.

12.6.3.3 Format of the SUSARs reports

Electronic reporting is the expected method for expedited reporting of SUSARs to the competent authority. The format and content as defined by the competent authority should be adhered to.

13 Evaluation of Results (Definitions and response/evaluation of outcome measures)

13.1 Trial Outcome Measures

13.1.1 Primary efficacy outcome measure

Change in tumour proliferation measured by Ki67 immuno-histochemical assessment (%) at baseline compared to Day 15 (+ ≤4 days).

Ki67 expression will be measured centrally, blinded with treatment allocation, using formalin-fixed, paraffin embedded histopathology sections of the tumour tissue specimens taken at baseline and at Day 15 (+ ≤4) days. Additional analyses will also be done on the tumour biopsy specimens taken at breast tumour excision (or the additional research biopsy if tumour excision is not performed on Day 15 + ≤4 days). IHC for the Ki67-proliferation associated antigen will be carried out on 3-4 µm sections of the routinely processed material following heat mediated antigen retrieval and with the appropriate primary antibodies.

The tumour-cell Ki67 antigen labeling index will be recorded following the

recommendations from the International Ki67 working group [19]. Sections from the diagnostic or baseline core biopsy, Day 15 (+ ≤4) tumour biopsy/post-surgical specimen will be stained for Ki67. Tumour cell nuclei showing any intensity of staining will be regarded as positive. The stained slide will be reviewed at low power and representative high power fields (x40) will be selected for counting; if there are clear areas with an increased proportion of cells staining (hot spots) these will be included in the count. One thousand tumour cell nuclei will be counted; if there are less than 1000 tumour cells in a biopsy a minimum of 500 will be counted. If there are less than 500 cell nuclei then further sections will be stained at a depth of 25μm and counted until the minimum number of cells is reached. Ki67 will be scored as the percentage of tumour nuclei staining. The investigators analyzing Ki67 will be blinded as to treatment allocation. Ki67-response is defined as a 50% or higher fall in Ki67 expression.

13.1.2 Secondary outcome measure

Secondary endpoints:

- Change in tumour apoptosis, measured by Caspase 3 (IHC) between baseline and Day 15 (+≤4 Days)
  Caspase-3 is synthesized as an inactive proenzyme which is activated by cleavage in cells undergoing apoptosis. Apoptotic cells can be detected more easily by IHC with an antibody for activated (cleaved) caspase-3. Capase-3 IHC has been validated as a marker of apoptosis in breast cancer [20]

- Change in expression of Androgen Receptor and PR by IHC between baseline and Day 15 (+≤4 Days)
  IHC of PR will be performed as a surrogate of ER activity, and to correlate Ki67 changes to PR levels. Like PR, Androgen Receptor influences ER-alpha activity in breast cancer, and has been shown to be a predictor of response to medroxyprogesterone acetate (a synthetic progestin) in breast cancer. We will correlate Ki67 changes to Androgen Receptor levels.

- Change in proliferation by Aurora Kinase A labeling by IHC between baseline and Day 15 (+≤4 Days)
  In a series of 3093 women with breast cancer, Aurora Kinase A by IHC was found to outperform other proliferation markers as an independent predictor of breast cancer specific survival in ER-positive breast cancer [21]. Given that Ki67 remains the current accepted proliferative marker, however, Aurora Kinase A will be analysed alongside

- Absolute value of Ki67 at day 15 (+≤4 Days)
  This is included as a secondary endpoint with a view to inform the development of a larger adjuvant trial following PIONEER. The absolute value of Ki67 at Day 15 has been found to be better predictive of recurrence free survival [16, 17]

Secondary safety endpoints:
- incidence of SAEs
- incidence of AEs of all grades (CTCAE V4.03)

13.1.3 Exploratory outcome measures

Other translational research questions will be addressed:

- Transcription factor mapping (ChIP-seq) of ER, conducted to assess the progestin-induced ER reprogramming, as was observed in pre-clinical models [5].
  - ChIP-seq is chromatin immunoprecipitation followed by high throughput DNA sequencing, allowing mapping of the genomic location of transcription factor binding and histone modifications. ChIP-seq will allow demonstration of the robust and predictable ERα binding to novel genomic loci, mediated by PR.
- Change in epithelial mesenchymal transition markers by IHC between baseline and Day 15 (+≤4 Days)
  - To address the question of whether the combination of letrozole and megestrol acetate affects the metastatic potential of ER-positive breast cancer, IHC will be performed to compare the pre- and post-treatment cytoplasmic expression of E-cadherin and N-cadherin.
- Correlate differences in response to treatments with breast cancer genomic profiling datasets
  - To delineate potential underlying germline, somatic and pharmacogenetic reasons for response/non-response to trial treatment. If available, whole genome sequencing data from patients consented and recruited to studies collecting this information may be referenced. We may also aim to correlate our findings within the 4 ER-positive integrative 'clusters' described by the Molecular Taxonomy of Breast Cancer International Consortium [22], based on copy number and gene expression data.

14 Statistics

14.1 Statistical methods

The primary outcome measure is the change in tumour proliferation measured by Ki-67 immuno-histochemical (IHC) assessment (%) at baseline and on day 15 (+ ≤4 days). The standard t-test will be applied for the primary comparison between Arm A vs Arm (B+C) based on evaluable patients.

The primary analysis for the primary endpoint will be based on the evaluable population (defined as eligible patients who have completed at least 13 [or 80%] of the full 15 Day [+ ≤4 Days] dosing schedule).
Adverse events will be summarized for the safety population (defined as all patients who have received at least one dose of protocol treatment) using frequency tables and compared using the Wilcoxon Mann-Whitney test or Kruskal Wallis test. All other secondary outcomes will be summarised using the summary statistics and compared between arms using t-test, ANOVA, Wilcoxon Mann-Whitney test or Kruskal Wallis test where ever is appropriate. A comprehensive statistical-analysis plan will be prepared before any analysis is performed.

14.2 Number of Subjects to be enrolled

In this open-label, three-arm, randomized, phase II trial; patients will be randomised in a ratio 1(A):1.5(B):1.5(C) in favour of the 2 combination arms of Letrozole + Megestrol acetate. Our hypothesis is that the addition of Megestrol acetate increases the anti-proliferative effect of Letrozole when given for 2 weeks pre-operatively in patients with early-stage, ER-positive breast cancer, as measured by Ki-67.

Based on the results from the *Opportune* study [23], where a significantly greater geometric mean Ki-67 suppression of 83.8% (one-sided 95% CI, ≥ 79.0%) was reported for the combination arm, and 66.0% (95% CI, ≤75.4%) for anastrozole alone

arm (geometric mean ratio [combination: anastrozole], 0.48; 95% CI, ≤0.72; P = .004), this translates to the estimated standard deviation is 0.242. We therefore have assumed a mean 66% reduction in Ki67 for the Letrozole alone arm of PIONEER (Arm A). It is anticipated that there is a mean 77.5% reduction in Ki67 in arms B and C for all patients (approximately one third PR negative, and two thirds PR positive), and a mean 80.0% reduction in Ki67 in arms B and C for patients with PR positive, which was based on the anti-proliferative effect seen in preclinical models [5]. Enrichment design is applied with the overall significance level of 5% (one-sided) (5% (α) = 2.5% (α_{all}) + 2.5% (α_{PR+})) and a power of 80%, and common standard deviation of 0.242, using the two-sample t-test comparing Arm A vs. Arm (B+C):

- all patients

a total of 189 patients (Arm A n=47, Arm B n=71, Arm C n=71) is needed to detect the mean reduction in Ki67 of 66% in Arm A to 77.5% in Arm (B+C);

- patients with PR positive breast cancer

a total of 149 PR positive patients (Arm A n=37, Arm B n=56, Arm C n=56) is needed to detect the mean reduction in Ki67 of 66% in Arm A to 80.0% in Arm (B+C);

The total number of evaluable patients required is 189 with 149 PR positive. It is anticipated that this will require approximately 10% more patients to be randomised, to account for patients that may not be evaluable.
It is therefore planned to randomise approximately 210 patients with 166 PR positive (210x90%=189, 166x90%=149).

The comparison of Ki67 reduction between Arm A and Arm (B+C) will be performed for all patients; if $p_{all} \leq 0.025$, it can be concluded that the addition of Megestrol acetate increases the anti-proliferative effect of Letrozole for all patients.

If there is no evidence of Ki67 reduction for all patients, the comparison of Ki67 reduction between Arm A and Arm (B+C) will be performed in patients with PR positive only; if $p_{PR+} \leq 0.025$, it can be concluded that the addition of Megestrol acetate increases the anti-proliferative effect of Letrozole for patients with PR positive.

14.3 **Enriched data monitoring**

The patient population will be monitored (PR positive vs. PR negative) regularly during patient recruitment in order to achieve the required sample size for patients with PR positive. This will be regularly reviewed by the Independent Data Monitoring Committee (IDMC) and the recruitment plan will be amended accordingly if needed.

14.4 **Criteria for the premature termination of the trial**

Patient recruitment will continue until the target of 189 evaluable patients has been reached.

14.5 **Procedure to account for missing or spurious data**

All patients who have completed the entire pre-operative window dosing schedule (i.e. at least 13 out of the 15 dosing Days or at least 80% for patients receiving 16-19 days of dosing) will be included in the final analyses and will be evaluable for assessment of the translational endpoints.

14.6 Definition of the end of the trial

The end of trial will be 12 months after the last patient's last visit, which will allow sufficient time for the translational endpoints to be investigated and the data cleaned for primary analyses and reports.

All essential trial documents (including patient notes) will be retained as per the sponsor's procedures and SOPs following the end of the trial. The trial coordinating team will notify the centres when trial documents may be destroyed.

15 Data handling and record keeping

15.1 Case Report Form (CRF)

All data will be transferred into a Case Report Form (CRF) which will be anonymised. All trial data in the CRF must be extracted from and be consistent with the relevant source documents. The CRFs must be completed, dated and signed by the investigator or designee in a timely manner. It remains the responsibility of the investigator for the timing, completeness, legibility and accuracy of the CRF pages. The CRF will be accessible to trial coordinators, data managers, the investigators, Clinical Trial Monitors, Auditors and Inspectors as required.

Completed originals of the CRFs should be posted to the PIONEER trial coordination centre within 2 weeks of the End of Trial visit:

PIONEER Trial Coordinator
CCTU-Cancer, Cambridge University Hospitals NHS Foundation Trust
Box 279 (S4), Addenbrooke's Hospital
Cambridge Biomedical Campus, Hill's Road
Cambridge CB2 0QQ

The investigator will retain a copy of each completed CRF page at site. If faxed, they will retain the original. The investigator will also supply the trial coordination centre with any required, anonymised background information from the medical records as required.
The investigators must ensure that the CRFs and other trial related documentation is sent to the trial coordination centre containing **no patient identifiable data**.

The investigator will retain all copies of the CRF in the relevant sections of their Investigator Site File with any required anonymised background information from the medical records as required.

All CRF pages must be clear, legible and completed in black ink. Any errors should be crossed with a single stroke so that the original entry can still be seen. Corrections should be inserted and the change dated and initialled by the investigator or designee. If it is not clear why the change has been made, an explanation should be written next to the change. Typing correction fluid must not be used.

Data management queries should be answered and submitted within 1 month.

15.2 Source Data

To enable peer review, monitoring, audit and/or inspection the investigator must agree to keep records of all participating patients (sufficient information to link records e.g.,

CRFs, hospital records and samples), all original signed IC forms and copies of the CRF pages.

Source data and documents to retain include, but not limited to:
- Patient medical records
- Informed consent forms
- On line test results
- Tissue and bloods sample logs
- Patient trial drug diaries
- CRFs: Ki67 assessment results and Androgen Receptor, PR, Caspase-3, Aurora Kinase A, E-Cadherin and N-Cadherin IHC Assessment results.

15.3 Data Protection & Patient Confidentiality

All investigators and trial site staff involved in this trial must comply with the requirements of the General Data Protection Regulation and Data Protection Act 2018 and Trust Policy with regards to the collection, storage, processing and disclosure of personal information and will uphold the Act's core principles.

15.4 Conduct of trial

The Principal Investigator at each participating site is required to supply the PIONEER Office with a current curriculum vitae and evidence of up-to-date GCP training before the trial is opened at the applicable site. They will also be required to complete a signature page for each new version of the PIONEER Protocol issued by the PIONEER office.
All site personnel involved in the conduct of the trial (at a minimum the Principal Investigator, a pharmacist, a pathologist or tissue bank staff, and a lead nurse or coordinator or data manager for the trial) will be asked to complete:
• Registration Forms,
• staff Signature and Delegation Logs,
• attend an initiation meeting which will cover trial rationale, Protocol procedures, and collection and reporting of data.
All staff involved in the trial should be listed on the delegation log and should have up to date training records (including GCP) available on request. Following this, all sites will be provided with an Investigator Site File and Pharmacy Site File containing instructional materials and documentation required for the conduct of the trial. The PIONEER office will offer continued support and training as necessary for all sites via telephone, fax, email, and mail. New site staff that did not complete initiation training will be offered initiation training by the PIONEER office, otherwise site staff present at the initiation training are able to train their new staff, and the trial initiation slides can be provided to sites for this purpose.

16 Storage and Analysis of Samples

The collection of pre and post treatment tumour tissue specimens are mandatory and part of the clinical trial. A research tissue from the diagnostic core biopsy (up to 2 months before patient starting treatment) will be required. Approximately 10 unstained slides are needed.

If archive diagnostic tissue blocks are provided, will be returned to the original pathology department at the end of the trial.

For patients who do not have tissue available from diagnosis, a research pre-treatment breast core biopsy will be obtained at baseline (Day -15 to Day 1). It is recommended that the tissue is obtained by ultrasound guided biopsy, when possible at the same time as surgical clip insertion (if required). For each biopsy, up to 5 passes (and a minimum of 2) will be acquired using a core biopsy needle as per institutional guidelines. At least 1 core should be fixed in 10% buffered neutral formalin.

Addenbrooke's patients only:The remaining cores should be snapped frozen in liquid nitrogen or on dry ice, transferred to a properly marked cryovial and stored in a -80°c freezer. Anonymised research samples will be stored in a -80°c freezer in a central facility in the Cambridge Institute (CRUK) Biorepository.

Similarly, post treatment samples will also be obtained, preferably during tumour excision to minimise the impact on the patient. More biopsy tissue may be acquired from the surgical specimen (up to 10 passes and a minimum of 2) depending on the size of the tumour.

It is recommended that tumour biopsies in formalin should remain in 10% neutral buffered formalin to ensure adequate fixation (as per local processes) and compatibility with pre-optimised downstream IHC methods, following which they can either be processed through to paraffin wax and embedded into a wax block immediately or first transferred to a 70% ethanol solution to be processed/embedded within a 72 hour window (as per local processes). Formalin fixed paraffin embedded tissue should be shipped ambient when available or samples can be grouped and sent in batches.

Research samples must be anonymised with a patient trial number, date of birth and initials before being sent by mail to the PIONEER Office.

Please refer to the PIONEER lab manual for details and instructions.

17 Independent Data Monitoring Committee/Trial Steering Committee

The PIONEER trial oversight will be provided by a number of committees, whose remit and relationships are described briefly here. Charters detailing the membership and remit of all committees will be written.

17.1 Trial Management Team (TMT)

The Chief Investigator (CI) and the Cambridge Clinical Trials Unit- Cancer Theme (CCTU-CT) are responsible for the day-to-day running of the trial as detailed in trial-specific procedures. They will work together as the Trial Management Team (TMT). The CCTU-CT will prepare reports for the Trial Management Group (TMG), Independent Data Monitoring Committee (IDMC) and Trial Steering Committee (TSC), including data monitoring of patient recruitment and PR status, and will make safety and progress reports to the Research Ethics Committee (REC) and MHRA and to other groups for their regulatory and ethics requirements as needed.

17.2 **Trial Management Group (TMG)**

The Trial Management Group (TMG) will meet approximately monthly to oversee the running of the trial. TMG members will review SAEs which have occurred in the trial. If there are specific safety concerns these may be raised with the TSC and IDMC. TMG members will be comprised of selected PIONEER trial co-investigators (including: CI, site PI, trial coordinators and others).

17.3 **Independent Data Monitoring Committee (IDMC)**

The Independent Data Monitoring Committee (IDMC) is independent of the PIONEER investigators. The group will meet approximately 12 months after the first patient is recruited, the meetings will then be held annually thereafter until the end of the randomised Phase II trial.

The IDMC will review reports from the CCTU-CT and give advice on continuing recruitment. There are no formal stopping rules for efficacy. A recommendation to discontinue recruitment (in all patients or in selected subgroups) will be made only if the emerging safety data indicate that the safety of the patients is not maintained. If a decision is made to continue, the IDMC will advise on the frequency of future reviews of the data on the basis of accrual and event rates. The IDMC will make recommendations to the TSC as to the continuation of the trial. Full details of the IDMC membership and remit can be found in the Independent Data Monitoring Committee (IDMC) Charter.

17.4 **Trial Steering Committee (TSC)**

The Trial Steering Committee (TSC) includes the TMG and also members who are independent of the trial investigators. It will provide overall supervision of the trial. It will meet at least once per year and will receive reports from the TMT, TMG and IDMC. Full details of the TSC membership and remit can be found in the TSC Charter.

17.5 **Relationship between Trial Committees**

The relationships between the various trial committees are shown in Figure 3.

Figure 3. Diagram of Relationships between Trial Committees

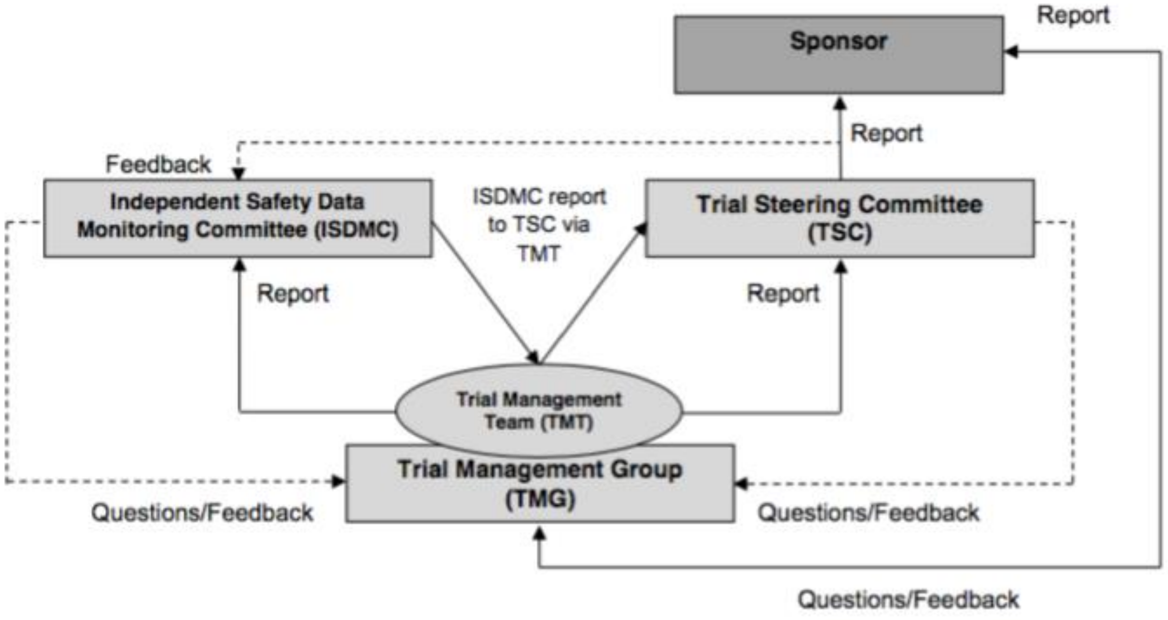

18 Ethical & Regulatory considerations

18.1 Consent

The Informed Consent (IC) form must be approved by the REC and must be in compliance with GCP, local regulatory requirements and legal requirements. The investigator must ensure that each trial subjects fully informed about the nature and objectives of the trial and possible risks associated with their participation.

The investigator or designated member of the local research team will obtain written IC from each patient before any trial-specific activity is performed. The IC form used for this trial and any change made during the course of this trial, must be prospectively approved by the REC. The investigator will retain the original of each patients signed IC form.

Should a patient require a verbal translation of the trial documentation by a locally approved interpreter/translator, it is the responsibility of the individual investigator to use locally approved translators.

If the trial requires documentation in a different language (other than English) the translation and back translation documents need to be reviewed and approved by the Sponsor prior to use. All sections of the approved documents must appear in the translation. The translated version must be appropriately dated and be version controlled.

Any new information which becomes available, which might affect the patient's willingness to continue participating in the trial will be communicated to the patient as soon as possible. If this is highlighted as an urgent safety issue, this information will need to be provided by telephone.

18.2 Ethical committee review

Before the start of the trial or implementation of any amendment we will obtain approval of the trial protocol, protocol amendments, IC forms and other relevant documents e.g., advertisements and General Practitioner (GP) information letters from the REC and the HRA, if applicable. All correspondence with the REC and the Health Research Authority (HRA) will be retained in the Trial Master File/Investigator Site File.

Annual reports will be submitted to the REC in accordance with national requirements. It is the CI's responsibility to produce the annual reports as required.

18.3 Regulatory Compliance

The trial will not commence until a CTA is obtained from the MHRA. The protocol and trial conduct will comply with the Medicines for Human Use (Clinical Trials) Regulations 2004 and any relevant amendments.

Development Safety Update Reports (DSURs) will be submitted to the MHRA in accordance with national requirements. It is the CI responsibility to produce the annual reports as required.

18.4 Protocol Amendments

Protocol amendments must be reviewed and agreement received from the Sponsor for all proposed amendments prior to submission to the REC and/or MHRA.

The only circumstance in which an amendment may be initiated prior to REC and/or MHRA approval is where the change is necessary to eliminate apparent, immediate risks to the patients (Urgent Safety Measures). In this case, accrual of new patients will be halted until the REC and/or MHRA approval has been obtained.

In the event of an Urgent Safety Measure, the central PIONEER trials team will notify each participating site/investigator by email within 24 hours or by the next working day.

18.5 Peer Review

This trial protocol has been reviewed by a departmental trial protocol group pre-approved by the trial sponsor, as well as 2 patient advocates and delegates from the AntiCancer Fund charity.

18.6 Declaration of Helsinki and Good Clinical Practice

The trial will be performed in accordance with the spirit and the letter of the declaration of Helsinki, the conditions and principles of GCP, the protocol and applicable local regulatory requirements and laws.

18.7 GCP Training

All trial staff must hold evidence of appropriate GCP training or undergo GCP training prior to undertaking any responsibilities on this trial. This training should be updated every 2 years or in accordance with participating sites' policy.

19 Sponsorship, Financial and Insurance

The trial is sponsored by Cambridge University Hospitals NHS Foundation Trust and University of Cambridge. The trial will be funded by the AntiCancer fund, with drug supply of Megestrol acetate 40mg supplied by Mawdsley-Brooks & Co Ltd to patients at no expense.

Cambridge University Hospitals NHS Foundation Trust, as a member of the NHS Clinical Negligence Scheme for Trusts, will accept full financial liability for harm caused to patients in the clinical trial caused through the negligence of its employees and honorary contract holders.

The University of Cambridge will arrange insurance for negligent harm caused as a result of protocol design and for non-negligent harm arising through participation in the clinical trial.

20 Monitoring, Audit & Inspection

The investigator must make all trial documentation and related records available should an MHRA Inspection occur. Should a monitoring visit or audit be requested, the investigator must make the trial documentation and source data available to the Sponsor's representative. All patient data must be handled and treated confidentially.

The Sponsor’s monitoring frequency will be determined by an initial risk assessment performed prior to the start of the trial. A detailed monitoring plan will be generated detailing the frequency and scope of the monitoring for the trial. Throughout the course of the trial, the risk assessment will be reviewed and the monitoring frequency adjusted as necessary.

Scheduled monitoring visits to other participating sites with be based on data queries, SAE reporting, protocol deviation reports. Otherwise, remote monitoring will be conducted for all participating sites. The scope and frequency of the monitoring will be determined by the risk assessment and detailed in the Monitoring Plan for the trial.

21 Protocol Compliance and Breaches of GCP

Prospective, planned deviations or waivers to the protocol are not allowed under the UK regulations on Clinical Trials and must not be used.

It is not acceptable to enrol a patient if they do not meet one or more eligibility criteria or restrictions specified in the trial protocol. If the eligibility criteria need to be changed, this amendment will first be approved by MHRA/REC via a substantial protocol amendment before they can be implemented.

Protocol deviations, non-compliances, or breaches are departures from the approved protocol. They can happen at any time, but are not planned. They must be adequately documented on the relevant forms and reported to the CI and Sponsor immediately.

Deviations from the protocol which are found to occur constantly again and again will not be accepted and will require immediate action and could potentially be classified as a serious breach.

Any potential/suspected serious breaches of GCP must be reported immediately to the Sponsor without any delay.

22 Publications policy

Ownership of the data arising from this trial resides with the TMG. On completion of the trial the data will be analysed and tabulated and a Final Trial Report prepared. The main trial results will be presented at national and international conferences and published in a peer-reviewed journal, on behalf of all collaborators. All presentations and publications relating to the trial must be authorised by the TMG.

The manuscript will be prepared by a writing group, appointed from amongst the TMG and high-accruing investigators. The PIONEER Office and all participating centres and Investigators, as well as the CCTU and AntiCancer Fund will be acknowledged in this publication. The first and last authorship of the main trial publication will be given to the trial CI and staff at the PIONEER Office. Other authorship will be given to Investigators ordered by recruitment.

23 References

1. Collaborative Group on Hormonal Factors in Breast Cancer. Breast cancer and breastfeeding: collaborative reanalysis of individual data from 47 epidemiological studies in 30 countries, including 50 302 women with breast cancer and 96 973 women without the disease. *The Lancet* **360**(9328):187-95 (2002).
2. Cancer Research UK Breast Cancer Statistics: http://publications.cancerresearchuk.org/downloads/product/CS_KF_BREAST.pdf
3. Dawson S-J, Rueda OM, Aparicio S and Caldas C. A new genome-driven integrated classification of breast cancer and its implications. *The EMBO J* **32**: 617-628 (2013).
4. Horwitz, K.B. & McGuire, W.L. Estrogen control of progesterone receptor in human breast cancer. Correlation with nuclear processing of estrogen receptor. *J Biol Chem* **253**, 2223-8 (1978).
5. Mohammed, H. et al. Progesterone receptor modulates ERα action in breast cancer. *Nature* **523**, 313-7 (2015).
6. Baird RD, Carroll JS. Understanding Oestrogen Receptor Function in Breast Cancer and its Interaction with the Progesterone Receptor. New Preclinical Findings and their Clinical Implications. *Clinical Oncology* **28**, 1-3 (2016).
7. Ingle, J. N. *et al.* Randomized clinical trial of Megestrol acetate versus tamoxifen in paramenopausal or castrated women with advanced breast cancer. *Am. J. Clin. Oncol.* **5,** 155–160 (1982)
8. Morgan, L. R. Megestrol acetate v tamoxifen in advanced breast cancer in postmenopausal patients. *Semin. Oncol.* **12,** 43–7 (1985)
9. Muss, H. B. *et al.* Megestrol acetate v tamoxifen in advanced breast cancer: a phase III trial of the Piedmont Oncology Association (POA). *Semin. Oncol.* **12,** 55–61 (1985)
10. Bines J, Dienstmann R, Obadia RM, et al. Activity of Megestrol acetate in postmenopausal women with advanced breast cancer after nonsteroidal aromatase inhibitor failure: a phase II trial. *Ann. Oncol.* **25**: 831e836 (2014).
11. Gill, P. G. *et al.* Randomized comparison of the effects of tamoxifen, Megestrol acetate, or tamoxifen plus Megestrol acetate on treatment response and survival in patients with metastatic breast cancer. *Ann. Oncol.* **4,** 741–4 (1993)

12. Francis PA, Regan MM, Fleming GF, et al. Adjuvant ovarian suppression in premenopausal breast cancer. *N Engl J Med* **372**: 436e446 (2015).
13. Davies C, Pan H, Godwin J, et al. Long-term effects of continuing adjuvant tamoxifen to 10 years versus stopping at 5 years after diagnosis of oestrogen receptor-positive breast cancer: ATLAS, a randomised trial. *Lancet* **6736**: 1e12 (2012).
14. Loprinzi CL, Michalak JC, Quella SK, et al. Megestrol acetate for the prevention of hot flashes. *N Engl J Med* **331**: 347e352 (1994).
15. Dowsett M, Ebbs SR, Dixon JM, et al: Biomarker changes during neoadjuvant anastrozole, tamoxifen, or the combination: Influence of hormonal status and HER-2 in breast cancer—A study from the IMPACT trialists. *J Clin Oncol* **23**: 2477-2492 (2005).
16. Dowsett M, Smith IE, Ebbs SR, et al: Prognostic value of Ki67 expression after short-term presurgical endocrine therapy for primary breast cancer. *J Natl Cancer Inst* **99**: 167-170 (2007).
17. Ellis MJ, Tao Y, Luo J, et al: Outcome prediction for estrogen receptor-positive breast cancer based on postneoadjuvant endocrine therapy tumor characteristics. *J Natl Cancer Inst* **100**: 1380-1388 (2008).
18. Baum M, Budzar AU, Cuzick J, Forbes J, Houghton JH, Klijn JG, et al. Anastrozole alone or in combination with tamoxifen versus tamoxifen alone for adjuvant treatment of postmenopausal women with early breast cancer: first results of the ATAC randomised trial. *Lancet* **359**:2131–9 (2002)
19. Provenzano E, Bossuyt V, Viale G, et al. Standardisation of pathological evaluation and reporting of postneoadjuvant specimens in clinical trials of breast cancer: recommendations from an international working group. *Modern Pathology* **28**, 1185-1201 (2015).
20. Sabine SS, Faratian D, Kirkegaard-Clausen T, Bartlett JMS. Validation of activated caspase-3 antibody staining as a marker of apoptosis in breast cancer. *Histopathology* **60**, 357–376 (2011)
21. Ali HR, Dawson S-J, Blows FM, Provenzano E et al. Aurora Kinase A outperforms Ki67 as a prognostic marker in ER-positive breast cancer. *British Journal of Cancer* **106**, 1798–1806 (2012)
22. Curtis C, Shah SP, Chin S-F, Turashvili G et al. The genomic and transcriptomic architecture of 2000 breast cancers reveals novel subgroups. Nature **486**(7403): 346–352 (2012).
23. Schmid P, Pinder SE, Wheatley D, Macaskill J et al. Phase III Randomised Pre-operative Window-of-Opportunity Study of the PI3K inhibitor Pictilisib Plus Anastrazole Compared with Anastrazole Alone in Patients with Estrogen Receptor-Positive Breast Cancer. *J Clin Onc* **34** (2016)
24. AJCC Cancer Staging Manual, Sixth Edition. Springer-Verlag, New York (2002).
25. ACRIN Study 6689 (2011) manual.
26. International Commission on Radiological Protection (ICRP). Radiation Dose to Patients from Radiopharmaceuticals - Addendum 4 to ICRP Publication 53. ICRP Publication 106. 2014 Ann. ICRP 38 (1-2).
27. Public Health England. Ionising Radiation Exposure of the UK Population: 2010 Review. 2016
28. International Commission on Radiological Protection (ICRP). The 2007 Recommendations of the International Commission on Radiological Protection. ICRP Publication 103. 2007 Ann. ICRP 37(24).

24 Appendices

Appendix 1 - Safety Reporting Flow Chart

Figure 1. Safety Reporting Flowchart for Participating Sites

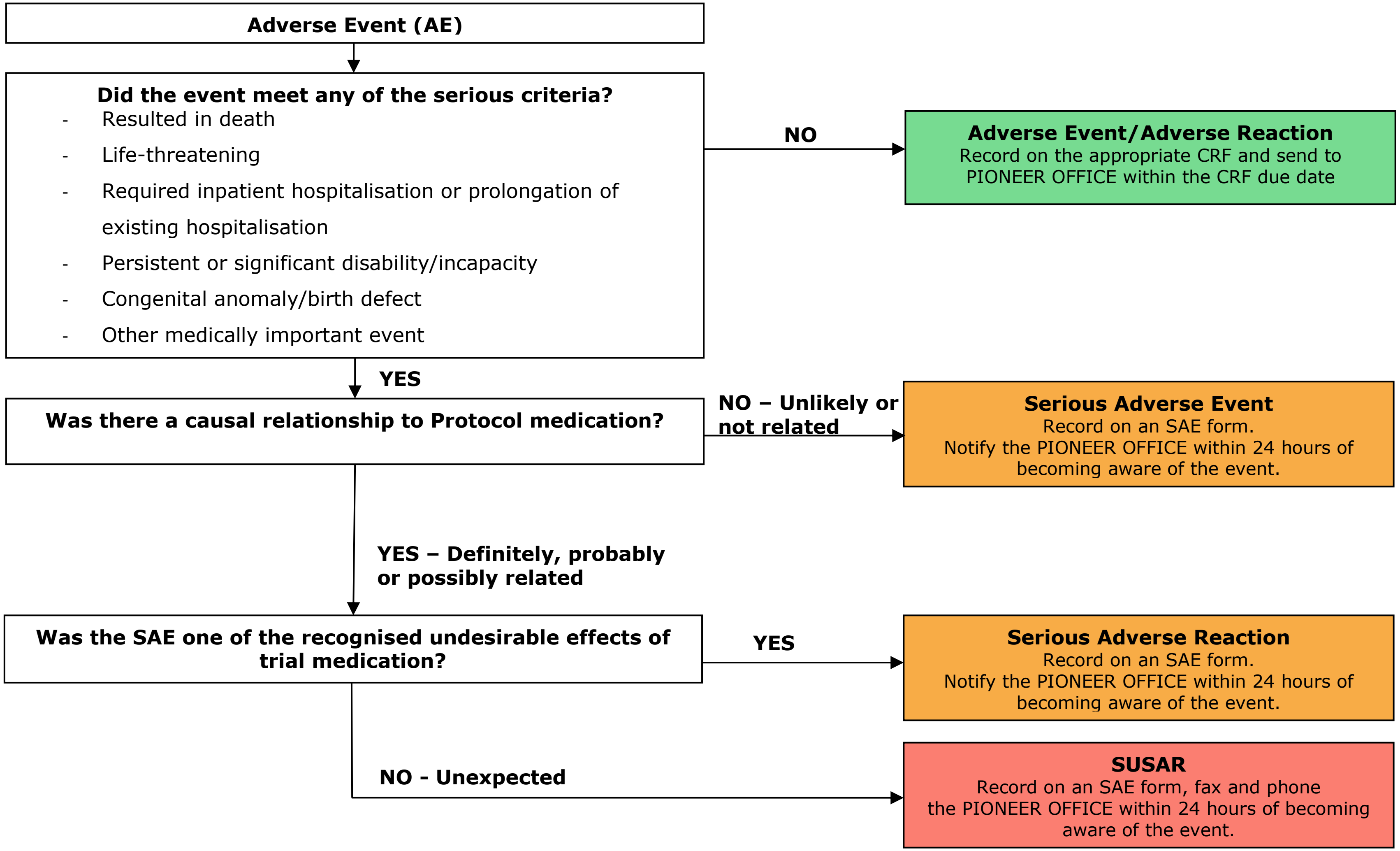

Appendix 2 - ECOG performance status

Grade	Description
0	Asymptomatic: normal activity
1	Symptomatic: fully ambulatory
2	Symptomatic: in bed < 50% of time
3	Symptomatic: in bed > 50% of time - not bedridden
4	100% bedridden
5	Death

Appendix 3 - TNM Staging System for Breast Cancer

Used with the permission of the American Joint Committee on Cancer (AJCC), Chicago, Illinois. The original and primary source for this information is the AJCC Cancer Staging Manual, Sixth Edition (2002) published by Springer-Verlag New York. (For more information, visit www.cancerstaging.net.) Any citation or quotation of this material must be credited to the AJCC as its primary source. The inclusion of this information herein does not authorize any reuse or further distribution without the expressed, written permission of Springer-Verlag New York, Inc., on behalf of the AJCC.

Definitions of TNM

Primary Tumour (T)

- TX Primary tumour cannot be assessed
- T0 No evidence of primary tumour
- Tis Carcinoma *in situ*: intraductal carcinoma, lobular carcinoma *in situ*, or Paget's disease of the nipple with no tumour
- T1 Tumour ≤2 cm in greatest dimension
  - T1mic Micro-invasion ≤0.1cm in greatest dimension
  - T1a Tumour >0.1 but ≤0.5 cm or less in greatest dimension
  - T1b Tumour >0.5 cm but ≤1 cm in greatest dimension
  - T1c Tumour >1 cm but ≤2 cm in greatest dimension
- T2 Tumour >2 cm but ≤5 cm in greatest dimension
- T3 Tumour >5 cm in greatest dimension
- T4 Tumour of any size with direct extension to (a) chest wall or (b) skin, only as described below.
  - T4a Extension to chest wall, not including pectoralis muscle
  - T4b Edema (including peau d'orange) or ulceration of the skin of the breast or satellite skin nodules confined to the same breast
  - T4c Both (T4a and T4b)
  - T4d Inflammatory carcinoma

Note: Paget's disease associated with a tumour is classified according to the size of the tumour.

Regional Lymph Nodes (N)

- NX Regional lymph nodes cannot be assessed (for example, previously removed)
- N0 No regional lymph node metastasis
- N1 Metastasis to movable ipsilateral axillary lymph node(s)
- N2 Metastasis to ipsilateral axillary lymph node(s) fixed or matted, or in clinically apparent ipsilateral internal mammary nodes in the absence of clinically evident* axillary lymph node metastasis
  - N2a Metastasis in ipsilateral axillary lymph nodes fixed (or matted) to one another or to other structures
  - N2b Metastasis only in clinically apparent* ipsilateral internal mammary nodes *and* in the absence of clinically evident axillary lymph node metastasis
- N3 Metastasis to ipsilateral infraclavicular lymph node(s) with or without axillary lymph node involvement, or in clinically apparent* ipsilateral internal mammary lymph node(s) and in the presence of clinically evident axillary or internal mammary lymph node involvement; or metastases in ipsilateral supraclavicular lymph node(s) with or without axillary or internal mammary lymph node involvement
  - N3a Metastasis in ipsilateral infraclavicular lymph node(s)
  - N3b Metastasis in ipsilateral internal mammary lymph node(s) and axillary lymph node(s)
  - N3c Metastasis in ipsilateral supraclavicular lymph node(s)

**** Clinically apparent is defined as detected by imaging trials (excluding lymphoscintigraphy) or by clinical examination or grossly visible pathologically.***

Distant Metastasis (M)

MX	Presence of distant metastasis cannot be assessed
M0	No distant metastasis
M1	Distant metastasis

Appendix 4 – ^{18}F-FLT PET/MR Imaging Sub-Study (Addenbrooke's Hospital only)

The scope of the imaging sub-study is to investigate the feasibility of assessing the effects of letrozole/letrozole+megestrol-acetate therapy utilising imaging biomarkers obtained from ^{18}F-FLT PET (cell proliferation) and MRI (angiogenesis, cellularity, tumour morphology). The relationships and changes observed in the derived imaging biomarkers will be used for the assessment of treatment response in a sub-cohort of patients participating in PIONEER, recruited from Cambridge.

Sub-study objectives

Primary objectives:

I. To measure changes in ^{18}F-FLT-PET and MR imaging indices during letrozole/letrozole+megestrol-acetate therapy;
II. To investigate pre- and post-therapy correlations between the histochemical proliferation marker Ki67 and PET/MR imaging measures;
III. To compare and correlate changes in Ki67 with changes in PET/MR imaging metrics after therapy.

Secondary Objective:

I. To investigate intra-tumoural heterogeneity in PET and MR imaging phenotypes and its correlation with the proliferative biomarker Ki67.

Summary of sub-study design

This is a prospective, exploratory sub-study on up to 15 postmenopausal women diagnosed with breast cancer and fulfilling the eligibility criteria for the PIONEER trial. Combined PET/MR imaging with ^{18}F-FLT will be carried out on the GE Signa PET/MRI, University of Cambridge at the Wolfson Brain Imaging Centre. ^{18}F-FLT will be produced by the Radiopharmaceutical Production Unit (RPU), Wolfson Brain Imaging Centre (WBIC), University of Cambridge. Patients will undergo PET/MRI examinations at two-time points: (*i*) baseline, and (*ii*) 11 (±1) days after the initiation of treatment.

Sub-study inclusion criteria

I. Criteria for inclusion into the PIONEER trial

Sub-study exclusion criteria

I. Exclusion criteria for the PIONEER trial

II. Contraindications to MRI, including implants known to be contraindicated at 3 Tesla
III. History of kidney disease or known allergic reaction to gadolinium-based contrast agent
IV. Previous tumour excision or radiotherapy for cancer to the ipsilateral breast or previous tumour excision to the ipsilateral breast within the past 4 months for benign breast disease
V. History of serious breast trauma within past 3 months
VI. Significant or uncontrolled medical problems which according to the opinion of the Principal Investigator render the participant unsuitable for participation in the sub-study
VII. Underlying conditions, including but not limited to medical or psychiatric conditions, which in the opinion of the Principal Investigator would preclude the participant from adhering to the sub-study protocol or completing the sub-study per protocol

Sub-study procedures and assessments

Patients eligible for inclusion into PIONEER and satisfying the additional criteria for participation into the imaging sub-study will be identified at the breast multi-disciplinary team (MDT) meeting.

Informed consent for this imaging sub-study will be obtained in accordance with the procedures detailed above in section 18 Consent.

Eligible patients will undergo ^{18}F-FLT-PET/MRI scanning on two occasions prior to their planned tumour excisionand/or end of trial core biopsy:

(i) prior to the initiation of PIONEER treatment (PIONEER baseline visit);
(ii) 11 (±1) days after treatment initiation.

<u>^{18}F-FLT-PET/MRI scans:</u>

Patients will not be required to fast prior to each PET/MRI examination. Patient height and weight will be recorded in the scan acquisition log for subsequent ^{18}F-FLT standardised uptake value (SUV) determination.

A venous cannula will be inserted for intravenous administration of 250 MBq ^{18}F-FLT. Syringe residue will be measured, and the corrected injected activity recorded in the scan acquisition log. The injected activity will be restricted to ±10% of the target activity of 250 MBq.

Another venous cannula will be inserted into one arm for intravenous administration of gadolinium-based contrast agent during imaging. This will be separate from the cannula employed for ^{18}F-FLT administration.

Patients will be scanned in the prone position, utilising a breast coil for MRI acquisition. PET/MRI acquisition will involve a single bed position covering the entire breast area. MRI

will include a dynamic contrast-enhanced protocol involving administration of gadolinium-based contrast.

Blood sampling and processing:

During imaging, up to five venous blood samples will be collected in order to determine the radioactivity concentration in whole blood and plasma, and the contribution of metabolites to blood plasma radioactivity. For the separation of ^{18}F-FLT from its metabolite (^{18}F-FLT glucuronide), venous blood samples will be analysed using solid-phase extraction chromatography as delineated in the ACRIN Study 6689 (2011) manual [25]. Analysis of the blood samples will be undertaken at the PET/MRI Scanner Suite, Wolfson Brain Imaging Centre, University of Cambridge. For each participant, the total blood volume acquired during imaging will be ≤50 mL. Owing to the short radioactive half-life of the tracer and the time-limited nature of these measurements, the blood samples acquired during imaging will be labelled with trial ID and timepoint/sample ID but not stored, and will be disposed of immediately after analysis.

Radiation dosimetry

The effective dose (ED) for ^{18}F-FLT has been estimated to be 0.015 mSv/MBq [26] for an adult patient. Hence, for an administered activity of 250 MBq , the effective radiation dose would be 3.75 mSv per PET/MRI examination. The total ED associated with the two ^{18}F-FLT PET/MRI examinations that are part of this research protocol is 7.5 mSv. This ED is equivalent to ~3.3 years of exposure to background radiation in the UK [27]. Using a risk estimate of detriment of 4.2%/Sv [28], the hypothetical risk of cancer (fatal or non-fatal) and of severe hereditary risks for the total research protocol dose is 1 in 3175. This can be considered in light of the natural incidence of fatal cancer, which is of the order of 1 in 4. The injected activity will be restricted to ±10% of the target activity of 250 MBq.

Analysis of ^{18}F-FLT-PET/MRI image data

^{18}F-FLT uptake as standardised uptake values ($SUV_{max/mean/peak}$) and tissue-to-plasma (T/P) ratios will be determined following tracer injection.

- Tumour volume (TV) will be defined on the most-enhancing phase of the dynamic contrast-enhanced MRI component and superimposed onto the PET images. Delineation of the tumour volume will be performed by two radiologists with experience in breast imaging in consensus.

- Thresholds between tumour and normal tissue will be defined using SUV and T/P values within the TV.

- Proliferative volume within the TV (cm^3) will be calculated as the number of voxels above the calculated SUV and T/P thresholds, multiplied by the voxel size (cm^3)

- Intra-tumoural voxel-based correlations will also be derived from MRI and ^{18}F-FLT PET images, and correlations between imaging metrics and the immunohistochemical proliferation marker Ki67 will be derived

Sub-study staff analysing all sub-study data will be blinded to patient to trial allocation.

Data management

Registration of PIONEER sub-study patients:

Patients will be registered into the PIONEER ^{18}F-FLT PET/MRI Imaging sub-study following confirmation of eligibility.

Local site staff will complete the additional sub-study sections of the **'informed consent'** and **'eligibility'CRFs** (not the original consent forms) and send them to the central PIONEER office at the time of patient registration and authorisation for randomisation to the main PIONEER trial (Please refer to PIONEER protocol section 10.1.2 Subject registration/randomisation).

The team should use the main patient's issued PIONEER trial ID for use on all sub-study data.

The sub-study will be coordinated by the PIONEER trial coordinator. Investigators and personnel involved in the sub-study must be listed on the delegation log in the PIONEER site file. Other information relating to the sub-study will be recorded and filed in the site file under the Sub-Study section.

Data collection:

The PET/MRI data will be stored using database management policies of the Wolfson Brain Imaging Centre, University of Cambridge. After acquisition, raw imaging data will be stored in non-anonymised format in a secure computer environment with data encryption and lockable filing cabinets with restricted access in the Wolfson Brain Imaging Centre, University of Cambridge. The data may also be transferred to the Cambridge University Hospital NHS Foundation Trust for clinical purposes. Imaging data will be kept for a minimum of 10 years after acquisition. Data transfers will be performed according to the NHS Code of Practice on Confidentiality.

The data for analysis will be in pseudo-anonymised form with all direct identifiers removed, and heavily restricted to study staff and members of the research team.

A summary of data will be recorded on the PIONEER CRF sub-study form to be provided to the PIONEER coordinator shortly after the patient visit.

Funding

Cancer Research UK – Cambridge Institute will be funding the imaging costs of this sub-study.

Further details of the ^{18}F-FLT PET/MR Imaging Sub-study are outlined in the Imaging Manual.

CONSORT 2010 checklist of information to include when reporting a randomised trial*

Section/Topic	Item No	Checklist item	Reported on page No
**Title and abstract**			
	1a	Identification as a randomised trial in the title	1
	1b	Structured summary of trial design, methods, results, and conclusions (for specific guidance see CONSORT for abstracts)	2
**Introduction**			
Background and objectives	2a	Scientific background and explanation of rationale	3
	2b	Specific objectives or hypotheses	3
**Methods**			
Trial design	3a	Description of trial design (such as parallel, factorial) including allocation ratio	4 and 10
	3b	Important changes to methods after trial commencement (such as eligibility criteria), with reasons	NA
Participants	4a	Eligibility criteria for participants	4 and 10
	4b	Settings and locations where the data were collected	10
Interventions	5	The interventions for each group with sufficient details to allow replication, including how and when they were actually administered	10
Outcomes	6a	Completely defined pre-specified primary and secondary outcome measures, including how and when they were assessed	10
	6b	Any changes to trial outcomes after the trial commenced, with reasons	NA
Sample size	7a	How sample size was determined	11
	7b	When applicable, explanation of any interim analyses and stopping guidelines	NA
Randomisation:			
Sequence generation	8a	Method used to generate the random allocation sequence	10
	8b	Type of randomisation; details of any restriction (such as blocking and block size)	10
Allocation concealment mechanism	9	Mechanism used to implement the random allocation sequence (such as sequentially numbered containers), describing any steps taken to conceal the sequence until interventions were assigned	10
Implementation	10	Who generated the random allocation sequence, who enrolled participants, and who assigned participants to interventions	10
Blinding	11a	If done, who was blinded after assignment to interventions (for example, participants, care providers, those	11

		assessing outcomes) and how	
	11b	If relevant, description of the similarity of interventions	NA
Statistical methods	12a	Statistical methods used to compare groups for primary and secondary outcomes	11-12
	12b	Methods for additional analyses, such as subgroup analyses and adjusted analyses	11-12
**Results**			
Participant flow (a diagram is strongly recommended)	13a	For each group, the numbers of participants who were randomly assigned, received intended treatment, and were analysed for the primary outcome	4
	13b	For each group, losses and exclusions after randomisation, together with reasons	4
Recruitment	14a	Dates defining the periods of recruitment and follow-up	4
	14b	Why the trial ended or was stopped	4
Baseline data	15	A table showing baseline demographic and clinical characteristics for each group	4 & 16
Numbers analysed	16	For each group, number of participants (denominator) included in each analysis and whether the analysis was by original assigned groups	4
Outcomes and estimation	17a	For each primary and secondary outcome, results for each group, and the estimated effect size and its precision (such as 95% confidence interval)	4 -5
	17b	For binary outcomes, presentation of both absolute and relative effect sizes is recommended	NA
Ancillary analyses	18	Results of any other analyses performed, including subgroup analyses and adjusted analyses, distinguishing pre-specified from exploratory	4-7
Harms	19	All important harms or unintended effects in each group (for specific guidance see CONSORT for harms)	5
**Discussion**			
Limitations	20	Trial limitations, addressing sources of potential bias, imprecision, and, if relevant, multiplicity of analyses	8-9
Generalisability	21	Generalisability (external validity, applicability) of the trial findings	8-9
Interpretation	22	Interpretation consistent with results, balancing benefits and harms, and considering other relevant evidence	8-9
**Other information**			
Registration	23	Registration number and name of trial registry	2 & 10
Protocol	24	Where the full trial protocol can be accessed, if available	10
Funding	25	Sources of funding and other support (such as supply of drugs), role of funders	14-15

Citation: Schulz KF, Altman DG, Moher D, for the CONSORT Group. CONSORT 2010 Statement: updated guidelines for reporting parallel group randomised trials. BMC Medicine. 2010;8:18.
© 2010 Schulz et al. This is an Open Access article distributed under the terms of the Creative Commons Attribution License (http://creativecommons.org/licenses/by/2.0), which permits unrestricted use, distribution, and reproduction in any medium, provided the original work is properly cited.

*We strongly recommend reading this statement in conjunction with the CONSORT 2010 Explanation and Elaboration for important clarifications on all the items. If relevant, we also recommend reading CONSORT extensions for cluster randomised trials, non-inferiority and equivalence trials, non-pharmacological treatments, herbal interventions, and pragmatic trials. Additional extensions are forthcoming: for those and for up-to-date references relevant to this checklist, see www.consort-statement.org.

Cambridge Personalised Breast Cancer Programme (PBCP)

Project team (in alphabetical order, by surname):

Addenbrooke's: Jean Abraham (Chief Investigator); Shubha Anand; Steven Bell; Samuel Casford; Elizabeth Cromwell; James Drummond; Alex Fulton; Clare Hannon; Justine Kane; Ollie Kane; Charlotte King; Jonathan Lay; Bin Liu; Rebecca Lucey; Zoe Matthews; Meena Murthy; Claudia Pallucca; Karen Pinilla; Elena Provenzano; Philip Schouten; Patrick Tarpey; Victoria Theobald; Katrina (Jiaqi) Xian; Deborah Whitehorn; Joanna Worley

Illumina: Maya Bajracharya; Jennifer Becq; David Bentley; Stefano Berri; Peter Campbell; Leonardo Carnielli; Kai Jie Chow; Claudiu Creanga; Jordi Denecker; Dagmara Furmanczyk; Terena James; Zoya Kingsbury; Marianna Kyritsi; Uma Maheswari; Martina Mijuskovic; Taksina Newington; Pablo Olmos Aguirre; Christine Rees; Mark Ross; Caleb Shelton; Eleni Tsogka; Mahesh Vasipalli; Dunja Vucenovic; Nicholas Walker

Past members (in alphabetical order, by surname):
Precision Breast Cancer Institute: Heather Biggs; Tracy Cook; Lynsey Drewett; Joanna Griffin; Emma Harrison; Linda Jones; Erica Law; Vanessa Moreira;
Cancer Molecular Diagnostics Laboratory: Mikel Valgañon
Medical Genetics: Howard Martin, Kim Brugger
Cambridge University Hospitals – East Genomics Laboratory Hub: Chris Corbin; Ahn JooWook
Cancer Research UK Cambridge Institute: Chris Boursnell; Carlos Caldas; Kate Eason; Paul Edwards; Suet Feung-Chin; Raquel Manzano Garcia; Oscar Rueda;
Illumina: Rebecca Doherty; Hariett Johnson; Fabrice Kamole; Margarida Lopes; John Peden; Helena West

Site Principal Investigators and team (in alphabetical order):
Jennifer Abaddulay; Katrina Cooke; Mukesh Mukesh [Colchester]; Emma Hanbury; Nicky Levitt; Petra Troszt [Oxford]. Paul Ridley; Chris Rose; Liz Sherwin [Ipswich].

Acknowledgements

Primarily to the patients and the families and friends who supported them for participating in this study.

Also to: NIHR Cambridge BRC for their support for staff and infrastructure costs; The Cancer Research UK Cambridge Centre Cambridge for their support for staff and infrastructure costs; Cancer Molecular Diagnostics Laboratory for their support for sample collection; The clinical trials support staff at all participating sites.

Funders
Addenbrookes Charitable Trust Grant [9800]; The Mark Foundation for Cancer Research and Cancer Research UK [C9685/A25177]; Cancer Research UK [A27657]
