# Supplementary material for: Evaluating progesterone receptor agonist megestrol plus letrozole for women with early-stage estrogen-receptor-positive breast cancer: the window-of-opportunity, randomized, phase 2b, PIONEER trial
Source: Nat Cancer. 2026 Jan 5;7(1):194–206. doi: 10.1038/s43018-025-01087-x (PMC12858400; doi:10.1038/s43018-025-01087-x)
Supplement: Supplementary file 2 — Reporting Summary [file 43018_2025_1087_MOESM2_ESM.pdf]

nature portfolio

Corresponding author(s): Dr. Richard Baird MD PhD

Last updated by author(s): Aug 8, 2025

Reporting Summary

Nature Portfolio wishes to improve the reproducibility of the work that we publish. This form provides structure for consistency and transparency in reporting. For further information on Nature Portfolio policies, see our Editorial Policies and the Editorial Policy Checklist.

Statistics

For all statistical analyses, confirm that the following items are present in the figure legend, table legend, main text, or Methods section.

n/a	Confirmed	
☐	☒	The exact sample size (*n*) for each experimental group/condition, given as a discrete number and unit of measurement
☐	☒	A statement on whether measurements were taken from distinct samples or whether the same sample was measured repeatedly
☐	☒	The statistical test(s) used AND whether they are one- or two-sided<br>*Only common tests should be described solely by name; describe more complex techniques in the Methods section.*
☐	☒	A description of all covariates tested
☐	☒	A description of any assumptions or corrections, such as tests of normality and adjustment for multiple comparisons
☐	☒	A full description of the statistical parameters including central tendency (e.g. means) or other basic estimates (e.g. regression coefficient) AND variation (e.g. standard deviation) or associated estimates of uncertainty (e.g. confidence intervals)
☐	☒	For null hypothesis testing, the test statistic (e.g. *F*, *t*, *r*) with confidence intervals, effect sizes, degrees of freedom and *P* value noted<br>*Give P values as exact values whenever suitable.*
☒	☐	For Bayesian analysis, information on the choice of priors and Markov chain Monte Carlo settings
☒	☐	For hierarchical and complex designs, identification of the appropriate level for tests and full reporting of outcomes
☒	☐	Estimates of effect sizes (e.g. Cohen's *d*, Pearson's *r*), indicating how they were calculated

Our web collection on statistics for biologists contains articles on many of the points above.

Software and code

Policy information about availability of computer code

Data collection	Trial data were collected with the MACRO database.
Data analysis	For analysis of trial/clinical data: R (V4.3.1) software ('coin' [v1.4-3] package, 'DescTools' [v 0.99.52] package, http://www.r-project.org/)<br>Variant calling and annotation: Isaac aligner (V 03.16.02.19), Germline SNV calling: strelka (v2.4.7). Variant annotation: Cancer Genome Interpreter v23.12.2). CNA Annotation: Canvas v1.3.1.012, R V4.3.3 ComplexHeatmapv2.15.4<br>Plots in figures 5,extended data figures 4 and 5: Rstudio 2024.09, RV4.3.3 (Tidyverse v2.0.0, ComplexHeatmap v2.24.1, circlize v0.4.16, ggplot2 v3.5.2, rstatix v0.7.2, emmeans v1.11.2, ggpubr v0.6.1, RColorBrewer v1.1-3, dplyr v1.1.4, forcats v1.0.0)<br>ChIPseq analysis: MATLAB R2015b (bowtie2 v 2.2.6 , MACS2 v 2.0.10.20131216 and 2.1.1.2016, bedtools v 2.26.0-97, 2.2.12, STAR version 2.5.1a. Code has been deposited at https://github.com/igorchern/jclabcode

For manuscripts utilizing custom algorithms or software that are central to the research but not yet described in published literature, software must be made available to editors and reviewers. We strongly encourage code deposition in a community repository (e.g. GitHub). See the Nature Portfolio guidelines for submitting code & software for further information.

Data

Policy information about availability of data

All manuscripts must include a data availability statement. This statement should provide the following information, where applicable:

- Accession codes, unique identifiers, or web links for publicly available datasets
- A description of any restrictions on data availability
- For clinical datasets or third party data, please ensure that the statement adheres to our policy

Data collected within the PIONEER study will be made available to researchers whose full proposal for their use of the data has been approved by the PIONEER Trial Management Group and whose research includes a clear and comprehensive research plan with statistical considerations adequately completed. The data required for the approved, specified purposes and the trial protocol will be provided, after completion of a data sharing agreement. Please address requests for data to rdb39@cam.ac.uk.
Deidentified ChiPseq data has been deposited in the Gene Expression Omnibus under the accession code GSE296953.
Deidentified source data for all figures in the manuscript is provided in the accompanying supplementary information.

Research involving human participants, their data, or biological material

Policy information about studies with human participants or human data. See also policy information about sex, gender (identity/presentation), and sexual orientation and race, ethnicity and racism.

Reporting on sex and gender	This was a clinical trial for female breast cancer patients, due to the rarity of male breast cancer in relation to the planned cohort size
Reporting on race, ethnicity, or other socially relevant groupings	The study included all participants who met eligibility criteria for the study with no data collected on race, ethnicity, or other socially relevant groupings.
Population characteristics	Eligible patients were post-menopausal women with histologically confirmed ER+ (Allred ≥3) HER2 negative breast cancer at least 10mm in size, with an ECOG performance status ≤2, planned for primary surgery or endocrine therapy.
Recruitment	Participants were assessed after written informed consent at 10 UK centres. Eligible participants were randomised 2:3:3 to three arms. All patients across all sites were assessed for eligibility criteria during their standard clinical evaluation. The trial was offered when it was considered clinically appropriate. There were no self selection or site-based biases involved. Participants were able to apply for reimbursement for a contribution towards additional travel expenses associated with trial participation up to a maximum of £20
Ethics oversight	The trial was approved by the United Kingdom Medicines and Healthcare Products Regulatory Agency and the North East - Newcastle & North Tyneside 1 Research Ethics Committee committee (17/NE/0113) (NCT03306472, Eudra-CT 2016-003752-79, IRAS 210677). The study design and conduct complied with all relevant regulations regarding the use of human study participants and was conducted in accordance with the criteria set by the Declaration of Helsinki

Note that full information on the approval of the study protocol must also be provided in the manuscript.

Field-specific reporting

Please select the one below that is the best fit for your research. If you are not sure, read the appropriate sections before making your selection.

☒ Life sciences ☐ Behavioural & social sciences ☐ Ecological, evolutionary & environmental sciences

For a reference copy of the document with all sections, see nature.com/documents/nr-reporting-summary-flat.pdf

Life sciences study design

All studies must disclose on these points even when the disclosure is negative.

Sample size	The PIONEER study used an enrichment design with an overall significance level of 5% (one-sided) (5% (α) = 2.5% (αall) + 2.5% (αPR+)) and a power of 80%. Assuming a common standard deviation of 0.242, a two-sample t-test comparing Arm A vs. Arm (B+C) required a total of 189 patients to detect a 66% reduction in arm A and 77.5% in arm (B+C) for Ki67 based on previous reports, and a total of 149 PR-positive patients on order to detect a mean reduction of 66% in arm A and 80.0% in arm (B+C) for Ki67.
Data exclusions	The primary endpoint, Ki67 analyses, were performed on a per-protocol population, including all patients that completed at least 13 days of study treatment, with paired (baseline and EOT) Ki67 assessment available. Safety analyses included all patients who had received at least one dose of study treatment.
Replication	The main analysis were performed by another independent statistician and checked against the results of the trial statistician at the final stage of the study. All replications were successful.
Randomization	Enrolled patients were randomised on a 2:3:3 ratio to three treatment arms: Arm A (Control) – letrozole 2.5mg only, Arm B (Research Arm 1)

Randomization	– letrozole 2.5mg + lower dose megestrol (40mg), Arm C (Research Arm 2) – letrozole 2.5mg + higher dose megestrol (160mg). Randomisation was stratified by ER ALLRED score, histological subtype (ductal/lobular) and tumour grade.
Blinding	This is an open label study. Slides were all scored centrally by a single expert histopathologist, blinded to treatment allocation and whether they were pre- or post-treatment samples.

Reporting for specific materials, systems and methods

We require information from authors about some types of materials, experimental systems and methods used in many studies. Here, indicate whether each material, system or method listed is relevant to your study. If you are not sure if a list item applies to your research, read the appropriate section before selecting a response.

Materials & experimental systems

n/a	Involved in the study
☐	☒ Antibodies
☒	☐ Eukaryotic cell lines
☒	☐ Palaeontology and archaeology
☒	☐ Animals and other organisms
☐	☒ Clinical data
☒	☐ Dual use research of concern
☒	☐ Plants

Methods

n/a	Involved in the study
☐	☒ ChIP-seq
☒	☐ Flow cytometry
☒	☐ MRI-based neuroimaging

Antibodies

Antibodies used	IHC: Ki67 (Clone MIB-1, Dako M7240 dilution 1/1000,), AURKA (Clone NCL-L-AK2 Novo Castra, 1/100), Progesterone Receptor (Clone PgR 636 M3569 Dako 1/100), Androgen Receptor (Clone AR441 M356201-1 M3562 Agilent, dilution 1/100), Estrogen Receptor (Clone EP1 M3643 Dako 1/40), cleaved Caspase 3 (Asp175) (Clone 5A1E #9664 Cell Signalling, 1/100).<br>ChIPseq: Millipore: 06-935, Abcam: ab3575 Lot numbers GR3217431-9 and 3045593
Validation	All of the above antibodies are either in routine clinical use (ER, PR, AR, Ki67 IHC antibodies) or have been previously validated in other studies (AURKA - Ali et al 2012, Cleaved caspase 3 - Schmid et al 2016). IHC was performed within a GCLP accredited laboratory with appropriate positive controls. ChIPseq antibodies have been previously validated (Glont et al 2019).

Clinical data

Policy information about clinical studies

All manuscripts should comply with the ICMJE guidelines for publication of clinical research and a completed CONSORT checklist must be included with all submissions.

Clinical trial registration	NCT03306472
Study protocol	Available in supplementary materials and uploaded separately as a full protocol.
Data collection	Participants were assessed after signing written informed consent and were randomised from July 2017 to October 2022 across ten UK hospitals. Data were collected and recorded for individual patients using anonymised Case Report Forms at participating sites, and then collated using the MACRO database by the Trial Coordination Team.
Outcomes	Primary Objective: To determine if the addition of Megestrol Acetate increases the anti-proliferative effect of Letrozole when given for 15 days pre-operatively in patients with early-stage, ER-positive breast cancer, as measured by change in Ki67.<br><br>Secondary Objectives:<br>- To compare and correlate the biological effects of Letrozole alone compared to Letrozole plus Megestrol Acetate using other immunohistochemical markers of tumour response: Caspase 3, Aurora kinase A, change in expression of the androgen and progesterone receptors, and the absolute value of Ki67 at Day 15<br>- To compare and correlate the change in Ki67 and the biological effects of low dose Megestrol Acetate compared to high dose Megestrol Acetate using other immunohistochemical markers of tumour response: Caspase 3, Aurora kinase A, change in expression of the androgen and progesterone receptors, and the absolute value of Ki67 at Day 15<br>- To assess the safety and tolerability of the combination of Letrozole +/- Megestrol Acetate by recording and assessing adverse and serious adverse events<br><br>Exploratory Outcomes:<br>-To assess progestin-induced ER reprogramming following treatment with Letrozole and Megestrol Acetate, using transcription factor mapping (ChIP-seq) of ER, and RNA sequencing.<br>- To correlate PIONEER efficacy and exploratory findings of this trial with breast cancer genomic profiling datasets.<br><br>Reporting of the study follows CONSORT 2010 Guidelines and a CONSORT Checklist is included within the Supporting Information

Plants

Seed stocks: NA

Novel plant genotypes: NA

Authentication: NA

ChIP-seq

Data deposition

☒ Confirm that both raw and final processed data have been deposited in a public database such as GEO.

☒ Confirm that you have deposited or provided access to graph files (e.g. BED files) for the called peaks.

Data access links
May remain private before publication.

ChIP-sequencing data that support the findings of this study have been deposited in the Gene Expression Omnibus (GEO) under accession code GSE296953. https://www.ncbi.nlm.nih.gov/geo/query/acc.cgi?acc=GSE296953

Files in database submission

jc5894_ER_letrozole_40mg_megestrol_acetate_CRI04.fq.gz
jc5894_ER_letrozole_40mg_megestrol_acetate_CRI06.fq.gz
jc5894_ER_letrozole_40mg_megestrol_acetate_CRI07.fq.gz
jc5894_ER_letrozole_40mg_megestrol_acetate_CRI08.fq.gz
jc5895_ER_letrozole_40mg_megestrol_acetate_CRI04.fq.gz
jc5896_1004_pre_ER_letrozole_160mg_megestrol_acetate_CRI04.fq.gz
jc5897_1004_post_ER_letrozole_160mg_megestrol_acetate_CRI04.fq.gz
jc5900_input_letrozole_40mg_megestrol_acetate_CRI04.fq.gz
jc5901_input_letrozole_40mg_megestrol_acetate_CRI04.fq.gz
jc5902_1004_pre_input_letrozole_160mg_megestrol_acetate_CRI04.fq.gz
jc5903_1004_post_input_letrozole_160mg_megestrol_acetate_CRI04.fq.gz
jc6000_ER_letrozole_alone_PRE_CRI07.fq.gz
jc6001_ER_letrozole_alone_POST_CRI07.fq.gz
jc6006_ER_letrozole_alone_PRE_CRI07.fq.gz
jc6007_ER_letrozole_alone_POST_CRI07.fq.gz
jc6272_ER_letrozole_160mg_megestrol_acetate_CRI01.fq.gz
jc6272_ER_letrozole_160mg_megestrol_acetate_CRI05.fq.gz
jc6273_ER_letrozole_160mg_megestrol_acetate_CRI01.fq.gz
jc6273_ER_letrozole_160mg_megestrol_acetate_CRI05.fq.gz
jc6274_ER_letrozole_alone_CRI01.fq.gz
jc6274_ER_letrozole_alone_CRI05.fq.gz
jc6275_ER_letrozole_alone_CRI01.fq.gz
jc6275_ER_letrozole_alone_CRI05.fq.gz
jc6276_ER_letrozole_160mg_megestrol_acetate_CRI01.fq.gz
jc6276_ER_letrozole_160mg_megestrol_acetate_CRI05.fq.gz
jc6277_ER_letrozole_160mg_megestrol_acetate_CRI01.fq.gz
jc6277_ER_letrozole_160mg_megestrol_acetate_CRI05.fq.gz
jc6278_input_letrozole_160mg_megestrol_acetate_CRI01.fq.gz
jc6278_input_letrozole_160mg_megestrol_acetate_CRI05.fq.gz
jc6279_input_letrozole_160mg_megestrol_acetate_CRI01.fq.gz
jc6279_input_letrozole_160mg_megestrol_acetate_CRI05.fq.gz
jc6281_input_letrozole_alone_CRI01.fq.gz
jc6281_input_letrozole_alone_CRI05.fq.gz
jc6282_input_letrozole_alone_CRI01.fq.gz
jc6282_input_letrozole_alone_CRI05.fq.gz
jc6283_input_letrozole_160mg_megestrol_acetate_CRI01.fq.gz
jc6283_input_letrozole_160mg_megestrol_acetate_CRI05.fq.gz
jc6284_input_letrozole_160mg_megestrol_acetate_CRI01.fq.gz
jc6284_input_letrozole_160mg_megestrol_acetate_CRI05.fq.gz
jc6520_ER_Arm_A_Letrozole_Only_CRI07.fq.gz
jc6520_ER_Arm_A_Letrozole_Only_CRI08.fq.gz
jc6521_ER_Arm_A_Letrozole_Only_CRI07.fq.gz
jc6521_ER_Arm_A_Letrozole_Only_CRI08.fq.gz
jc6522_ER_Arm_C_Letrozole_and_Megestrol_Acetate_160mg_CRI07.fq.gz
jc6522_ER_Arm_C_Letrozole_and_Megestrol_Acetate_160mg_CRI08.fq.gz
jc6523_ER_Arm_C_Letrozole_and_Megestrol_Acetate_160mg_CRI07.fq.gz
jc6523_ER_Arm_C_Letrozole_and_Megestrol_Acetate_160mg_CRI08.fq.gz

jc6524_ER_Arm_B_Letrozole_and_Megestrol_Acetate_40mg_CRI07.fq.gz
jc6524_ER_Arm_B_Letrozole_and_Megestrol_Acetate_40mg_CRI08.fq.gz
jc6525_ER_Arm_B_Letrozole_and_Megestrol_Acetate_40mg_CRI07.fq.gz
jc6525_ER_Arm_B_Letrozole_and_Megestrol_Acetate_40mg_CRI08.fq.gz
jc6526_ER_Arm_A_Letrozole_Only_CRI07.fq.gz
jc6526_ER_Arm_A_Letrozole_Only_CRI08.fq.gz
jc6527_ER_Arm_A_Letrozole_Only_CRI07.fq.gz
jc6527_ER_Arm_A_Letrozole_Only_CRI08.fq.gz
jc6528_ER_Arm_C_Letrozole_and_Megestrol_Acetate_160mg_CRI07.fq.gz
jc6528_ER_Arm_C_Letrozole_and_Megestrol_Acetate_160mg_CRI08.fq.gz
jc6529_ER_Arm_C_Letrozole_and_Megestrol_Acetate_160mg_CRI07.fq.gz
jc6529_ER_Arm_C_Letrozole_and_Megestrol_Acetate_160mg_CRI08.fq.gz
jc6530_ER_Arm_B_Letrozole_and_Megestrol_Acetate_40mg_CRI07.fq.gz
jc6530_ER_Arm_B_Letrozole_and_Megestrol_Acetate_40mg_CRI08.fq.gz
jc6531_ER_Arm_B_Letrozole_and_Megestrol_Acetate_40mg_CRI07.fq.gz
jc6531_ER_Arm_B_Letrozole_and_Megestrol_Acetate_40mg_CRI08.fq.gz
jc6532_ER_Arm_B_Letrozole_and_Megestrol_Acetate_40mg_CRI07.fq.gz
jc6532_ER_Arm_B_Letrozole_and_Megestrol_Acetate_40mg_CRI08.fq.gz
jc6533_ER_Arm_B_Letrozole_and_Megestrol_Acetate_40mg_CRI07.fq.gz
jc6533_ER_Arm_B_Letrozole_and_Megestrol_Acetate_40mg_CRI08.fq.gz
jc6534_ER_Arm_A_Letrozole_Only_CRI07.fq.gz
jc6534_ER_Arm_A_Letrozole_Only_CRI08.fq.gz
jc6535_ER_Arm_A_Letrozole_Only_CRI07.fq.gz
jc6535_ER_Arm_A_Letrozole_Only_CRI08.fq.gz
jc6538_ER_Arm_B_Letrozole_and_Megestrol_Acetate_40mg_CRI07.fq.gz
jc6538_ER_Arm_B_Letrozole_and_Megestrol_Acetate_40mg_CRI08.fq.gz
jc6539_ER_Arm_B_Letrozole_and_Megestrol_Acetate_40mg_CRI07.fq.gz
jc6539_ER_Arm_B_Letrozole_and_Megestrol_Acetate_40mg_CRI08.fq.gz
jc6540_ER_Arm_A_Letrozole_Only_CRI07.fq.gz
jc6540_ER_Arm_A_Letrozole_Only_CRI08.fq.gz
jc6541_ER_Arm_A_Letrozole_Only_CRI07.fq.gz
jc6541_ER_Arm_A_Letrozole_Only_CRI08.fq.gz
jc6582_ER_Arm_B_Letrozole_and_Megestrol_Acetate_40mg_CRI05.fq.gz
jc6582_ER_Arm_B_Letrozole_and_Megestrol_Acetate_40mg_CRI06.fq.gz
jc6583_ER_Arm_B_Letrozole_and_Megestrol_Acetate_40mg_CRI05.fq.gz
jc6583_ER_Arm_B_Letrozole_and_Megestrol_Acetate_40mg_CRI06.fq.gz
jc6584_ER_Arm_C_Letrozole_and_Megestrol_Acetate_160mg_CRI05.fq.gz
jc6584_ER_Arm_C_Letrozole_and_Megestrol_Acetate_160mg_CRI06.fq.gz
jc6585_ER_Arm_C_Letrozole_and_Megestrol_Acetate_160mg_CRI05.fq.gz
jc6585_ER_Arm_C_Letrozole_and_Megestrol_Acetate_160mg_CRI06.fq.gz
jc6586_ER_Arm_B_Letrozole_and_Megestrol_Acetate_40mg_CRI05.fq.gz
jc6586_ER_Arm_B_Letrozole_and_Megestrol_Acetate_40mg_CRI06.fq.gz
jc6587_ER_Arm_B_Letrozole_and_Megestrol_Acetate_40mg_CRI05.fq.gz
jc6587_ER_Arm_B_Letrozole_and_Megestrol_Acetate_40mg_CRI06.fq.gz
jc6588_ER_Arm_B_Letrozole_and_Megestrol_Acetate_40mg_CRI05.fq.gz
jc6588_ER_Arm_B_Letrozole_and_Megestrol_Acetate_40mg_CRI06.fq.gz
jc6589_ER_Arm_B_Letrozole_and_Megestrol_Acetate_40mg_CRI05.fq.gz
jc6589_ER_Arm_B_Letrozole_and_Megestrol_Acetate_40mg_CRI06.fq.gz
jc6590_ER_Arm_B_Letrozole_and_Megestrol_Acetate_40mg_CRI05.fq.gz
jc6590_ER_Arm_B_Letrozole_and_Megestrol_Acetate_40mg_CRI06.fq.gz
jc6591_ER_Arm_B_Letrozole_and_Megestrol_Acetate_40mg_CRI05.fq.gz
jc6591_ER_Arm_B_Letrozole_and_Megestrol_Acetate_40mg_CRI06.fq.gz
jc6592_ER_Arm_C_Letrozole_and_Megestrol_Acetate_160mg_CRI05.fq.gz
jc6592_ER_Arm_C_Letrozole_and_Megestrol_Acetate_160mg_CRI06.fq.gz
jc6593_ER_Arm_C_Letrozole_and_Megestrol_Acetate_160mg_CRI05.fq.gz
jc6593_ER_Arm_C_Letrozole_and_Megestrol_Acetate_160mg_CRI06.fq.gz
jc6594_ER_Arm_B_Letrozole_and_Megestrol_Acetate_40mg_CRI05.fq.gz
jc6594_ER_Arm_B_Letrozole_and_Megestrol_Acetate_40mg_CRI06.fq.gz
jc6595_ER_Arm_B_Letrozole_and_Megestrol_Acetate_40mg_CRI05.fq.gz
jc6595_ER_Arm_B_Letrozole_and_Megestrol_Acetate_40mg_CRI06.fq.gz
jc6596_ER_Arm_B_Letrozole_and_Megestrol_Acetate_40mg_CRI05.fq.gz
jc6596_ER_Arm_B_Letrozole_and_Megestrol_Acetate_40mg_CRI06.fq.gz
jc6597_ER_Arm_B_Letrozole_and_Megestrol_Acetate_40mg_CRI05.fq.gz
jc6597_ER_Arm_B_Letrozole_and_Megestrol_Acetate_40mg_CRI06.fq.gz
jc6671_ER_Arm_A_Letrozole_alone_CRI02.fq.gz
jc6671_ER_Arm_A_Letrozole_alone_CRI03.fq.gz
jc6671_ER_Arm_A_Letrozole_alone_CRI04.fq.gz
jc6671_ER_Arm_A_Letrozole_alone_CRI05.fq.gz
jc6671_ER_Arm_A_Letrozole_alone_CRI06.fq.gz
jc6672_ER_Arm_A_Letrozole_alone_CRI02.fq.gz
jc6672_ER_Arm_A_Letrozole_alone_CRI03.fq.gz
jc6672_ER_Arm_A_Letrozole_alone_CRI04.fq.gz
jc6672_ER_Arm_A_Letrozole_alone_CRI05.fq.gz
jc6672_ER_Arm_A_Letrozole_alone_CRI06.fq.gz
jc6673_ER_Arm_C_Letrozole_and_Megestrol_Acetate_160mg_CRI02.fq.gz
jc6673_ER_Arm_C_Letrozole_and_Megestrol_Acetate_160mg_CRI03.fq.gz

jc6673_ER_Arm_C_Letrozole_and_Megestrol_Acetate_160mg_CRI04.fq.gz
jc6673_ER_Arm_C_Letrozole_and_Megestrol_Acetate_160mg_CRI05.fq.gz
jc6673_ER_Arm_C_Letrozole_and_Megestrol_Acetate_160mg_CRI06.fq.gz
jc6674_ER_Arm_C_Letrozole_and_Megestrol_Acetate_160mg_CRI02.fq.gz
jc6674_ER_Arm_C_Letrozole_and_Megestrol_Acetate_160mg_CRI03.fq.gz
jc6674_ER_Arm_C_Letrozole_and_Megestrol_Acetate_160mg_CRI04.fq.gz
jc6674_ER_Arm_C_Letrozole_and_Megestrol_Acetate_160mg_CRI05.fq.gz
jc6674_ER_Arm_C_Letrozole_and_Megestrol_Acetate_160mg_CRI06.fq.gz
jc6675_ER_Arm_B_Letrozole_and_Megestrol_Acetate_40mg_CRI02.fq.gz
jc6675_ER_Arm_B_Letrozole_and_Megestrol_Acetate_40mg_CRI03.fq.gz
jc6675_ER_Arm_B_Letrozole_and_Megestrol_Acetate_40mg_CRI04.fq.gz
jc6675_ER_Arm_B_Letrozole_and_Megestrol_Acetate_40mg_CRI05.fq.gz
jc6675_ER_Arm_B_Letrozole_and_Megestrol_Acetate_40mg_CRI06.fq.gz
jc6676_ER_Arm_B_Letrozole_and_Megestrol_Acetate_40mg_CRI02.fq.gz
jc6676_ER_Arm_B_Letrozole_and_Megestrol_Acetate_40mg_CRI03.fq.gz
jc6676_ER_Arm_B_Letrozole_and_Megestrol_Acetate_40mg_CRI04.fq.gz
jc6676_ER_Arm_B_Letrozole_and_Megestrol_Acetate_40mg_CRI05.fq.gz
jc6676_ER_Arm_B_Letrozole_and_Megestrol_Acetate_40mg_CRI06.fq.gz
jc6679_ER_Arm_B_Letrozole_and_Megestrol_Acetate_40mg_CRI02.fq.gz
jc6679_ER_Arm_B_Letrozole_and_Megestrol_Acetate_40mg_CRI03.fq.gz
jc6679_ER_Arm_B_Letrozole_and_Megestrol_Acetate_40mg_CRI04.fq.gz
jc6679_ER_Arm_B_Letrozole_and_Megestrol_Acetate_40mg_CRI05.fq.gz
jc6679_ER_Arm_B_Letrozole_and_Megestrol_Acetate_40mg_CRI06.fq.gz
jc6680_ER_Arm_B_Letrozole_and_Megestrol_Acetate_40mg_CRI02.fq.gz
jc6680_ER_Arm_B_Letrozole_and_Megestrol_Acetate_40mg_CRI03.fq.gz
jc6680_ER_Arm_B_Letrozole_and_Megestrol_Acetate_40mg_CRI04.fq.gz
jc6680_ER_Arm_B_Letrozole_and_Megestrol_Acetate_40mg_CRI05.fq.gz
jc6680_ER_Arm_B_Letrozole_and_Megestrol_Acetate_40mg_CRI06.fq.gz
jc6681_ER_Arm_A_Letrozole_alone_CRI02.fq.gz
jc6681_ER_Arm_A_Letrozole_alone_CRI03.fq.gz
jc6681_ER_Arm_A_Letrozole_alone_CRI04.fq.gz
jc6681_ER_Arm_A_Letrozole_alone_CRI05.fq.gz
jc6681_ER_Arm_A_Letrozole_alone_CRI06.fq.gz
jc6682_ER_Arm_A_Letrozole_alone_CRI02.fq.gz
jc6682_ER_Arm_A_Letrozole_alone_CRI03.fq.gz
jc6682_ER_Arm_A_Letrozole_alone_CRI04.fq.gz
jc6682_ER_Arm_A_Letrozole_alone_CRI05.fq.gz
jc6682_ER_Arm_A_Letrozole_alone_CRI06.fq.gz
jc6687_ER_Arm_B_Letrozole_and_Megestrol_Acetate_40mg_CRI02.fq.gz
jc6687_ER_Arm_B_Letrozole_and_Megestrol_Acetate_40mg_CRI03.fq.gz
jc6687_ER_Arm_B_Letrozole_and_Megestrol_Acetate_40mg_CRI04.fq.gz
jc6687_ER_Arm_B_Letrozole_and_Megestrol_Acetate_40mg_CRI05.fq.gz
jc6687_ER_Arm_B_Letrozole_and_Megestrol_Acetate_40mg_CRI06.fq.gz
jc6688_ER_Arm_B_Letrozole_and_Megestrol_Acetate_40mg_CRI02.fq.gz
jc6688_ER_Arm_B_Letrozole_and_Megestrol_Acetate_40mg_CRI03.fq.gz
jc6688_ER_Arm_B_Letrozole_and_Megestrol_Acetate_40mg_CRI04.fq.gz
jc6688_ER_Arm_B_Letrozole_and_Megestrol_Acetate_40mg_CRI05.fq.gz
jc6688_ER_Arm_B_Letrozole_and_Megestrol_Acetate_40mg_CRI06.fq.gz
jc6691_ER_Arm_A_Letrozole_alone_CRI02.fq.gz
jc6691_ER_Arm_A_Letrozole_alone_CRI03.fq.gz
jc6691_ER_Arm_A_Letrozole_alone_CRI04.fq.gz
jc6691_ER_Arm_A_Letrozole_alone_CRI05.fq.gz
jc6691_ER_Arm_A_Letrozole_alone_CRI06.fq.gz
jc6692_ER_Arm_A_Letrozole_alone_CRI02.fq.gz
jc6692_ER_Arm_A_Letrozole_alone_CRI03.fq.gz
jc6692_ER_Arm_A_Letrozole_alone_CRI04.fq.gz
jc6692_ER_Arm_A_Letrozole_alone_CRI05.fq.gz
jc6692_ER_Arm_A_Letrozole_alone_CRI06.fq.gz
jc6703_input_Arm_A_Letrozole_alone_CRI02.fq.gz
jc6703_input_Arm_A_Letrozole_alone_CRI03.fq.gz
jc6703_input_Arm_A_Letrozole_alone_CRI04.fq.gz
jc6703_input_Arm_A_Letrozole_alone_CRI05.fq.gz
jc6703_input_Arm_A_Letrozole_alone_CRI06.fq.gz
jc6704_input_Arm_A_Letrozole_alone_CRI02.fq.gz
jc6704_input_Arm_A_Letrozole_alone_CRI03.fq.gz
jc6704_input_Arm_A_Letrozole_alone_CRI04.fq.gz
jc6704_input_Arm_A_Letrozole_alone_CRI05.fq.gz
jc6704_input_Arm_A_Letrozole_alone_CRI06.fq.gz
jc6705_input_Arm_C_Letrozole_and_Megestrol_Acetate_160mg_CRI02.fq.gz
jc6705_input_Arm_C_Letrozole_and_Megestrol_Acetate_160mg_CRI03.fq.gz
jc6705_input_Arm_C_Letrozole_and_Megestrol_Acetate_160mg_CRI04.fq.gz
jc6705_input_Arm_C_Letrozole_and_Megestrol_Acetate_160mg_CRI05.fq.gz
jc6705_input_Arm_C_Letrozole_and_Megestrol_Acetate_160mg_CRI06.fq.gz
jc6706_input_Arm_C_Letrozole_and_Megestrol_Acetate_160mg_CRI02.fq.gz
jc6706_input_Arm_C_Letrozole_and_Megestrol_Acetate_160mg_CRI03.fq.gz
jc6706_input_Arm_C_Letrozole_and_Megestrol_Acetate_160mg_CRI04.fq.gz

jc6706_input_Arm_C_Letrozole_and_Megestrol_Acetate_160mg_CRI05.fq.gz
jc6706_input_Arm_C_Letrozole_and_Megestrol_Acetate_160mg_CRI06.fq.gz
jc6707_input_Arm_B_Letrozole_and_Megestrol_Acetate_40mg_CRI02.fq.gz
jc6707_input_Arm_B_Letrozole_and_Megestrol_Acetate_40mg_CRI03.fq.gz
jc6707_input_Arm_B_Letrozole_and_Megestrol_Acetate_40mg_CRI04.fq.gz
jc6707_input_Arm_B_Letrozole_and_Megestrol_Acetate_40mg_CRI05.fq.gz
jc6707_input_Arm_B_Letrozole_and_Megestrol_Acetate_40mg_CRI06.fq.gz
jc6708_input_Arm_B_Letrozole_and_Megestrol_Acetate_40mg_CRI02.fq.gz
jc6708_input_Arm_B_Letrozole_and_Megestrol_Acetate_40mg_CRI03.fq.gz
jc6708_input_Arm_B_Letrozole_and_Megestrol_Acetate_40mg_CRI04.fq.gz
jc6708_input_Arm_B_Letrozole_and_Megestrol_Acetate_40mg_CRI05.fq.gz
jc6708_input_Arm_B_Letrozole_and_Megestrol_Acetate_40mg_CRI06.fq.gz
jc6711_input_Arm_B_Letrozole_and_Megestrol_Acetate_40mg_CRI02.fq.gz
jc6711_input_Arm_B_Letrozole_and_Megestrol_Acetate_40mg_CRI03.fq.gz
jc6711_input_Arm_B_Letrozole_and_Megestrol_Acetate_40mg_CRI04.fq.gz
jc6711_input_Arm_B_Letrozole_and_Megestrol_Acetate_40mg_CRI05.fq.gz
jc6711_input_Arm_B_Letrozole_and_Megestrol_Acetate_40mg_CRI06.fq.gz
jc6712_input_Arm_B_Letrozole_and_Megestrol_Acetate_40mg_CRI02.fq.gz
jc6712_input_Arm_B_Letrozole_and_Megestrol_Acetate_40mg_CRI03.fq.gz
jc6712_input_Arm_B_Letrozole_and_Megestrol_Acetate_40mg_CRI04.fq.gz
jc6712_input_Arm_B_Letrozole_and_Megestrol_Acetate_40mg_CRI05.fq.gz
jc6712_input_Arm_B_Letrozole_and_Megestrol_Acetate_40mg_CRI06.fq.gz
jc6713_input_Arm_A_Letrozole_alone_CRI02.fq.gz
jc6713_input_Arm_A_Letrozole_alone_CRI03.fq.gz
jc6713_input_Arm_A_Letrozole_alone_CRI04.fq.gz
jc6713_input_Arm_A_Letrozole_alone_CRI05.fq.gz
jc6713_input_Arm_A_Letrozole_alone_CRI06.fq.gz
jc6714_input_Arm_A_Letrozole_alone_CRI02.fq.gz
jc6714_input_Arm_A_Letrozole_alone_CRI03.fq.gz
jc6714_input_Arm_A_Letrozole_alone_CRI04.fq.gz
jc6714_input_Arm_A_Letrozole_alone_CRI05.fq.gz
jc6714_input_Arm_A_Letrozole_alone_CRI06.fq.gz
jc6719_input_Arm_B_Letrozole_and_Megestrol_Acetate_40mg_CRI02.fq.gz
jc6719_input_Arm_B_Letrozole_and_Megestrol_Acetate_40mg_CRI03.fq.gz
jc6719_input_Arm_B_Letrozole_and_Megestrol_Acetate_40mg_CRI04.fq.gz
jc6719_input_Arm_B_Letrozole_and_Megestrol_Acetate_40mg_CRI05.fq.gz
jc6719_input_Arm_B_Letrozole_and_Megestrol_Acetate_40mg_CRI06.fq.gz
jc6720_input_Arm_B_Letrozole_and_Megestrol_Acetate_40mg_CRI02.fq.gz
jc6720_input_Arm_B_Letrozole_and_Megestrol_Acetate_40mg_CRI03.fq.gz
jc6720_input_Arm_B_Letrozole_and_Megestrol_Acetate_40mg_CRI04.fq.gz
jc6720_input_Arm_B_Letrozole_and_Megestrol_Acetate_40mg_CRI05.fq.gz
jc6720_input_Arm_B_Letrozole_and_Megestrol_Acetate_40mg_CRI06.fq.gz
jc6723_input_Arm_A_Letrozole_alone_CRI02.fq.gz
jc6723_input_Arm_A_Letrozole_alone_CRI03.fq.gz
jc6723_input_Arm_A_Letrozole_alone_CRI04.fq.gz
jc6723_input_Arm_A_Letrozole_alone_CRI05.fq.gz
jc6723_input_Arm_A_Letrozole_alone_CRI06.fq.gz
jc6724_input_Arm_A_Letrozole_alone_CRI02.fq.gz
jc6724_input_Arm_A_Letrozole_alone_CRI03.fq.gz
jc6724_input_Arm_A_Letrozole_alone_CRI04.fq.gz
jc6724_input_Arm_A_Letrozole_alone_CRI05.fq.gz
jc6724_input_Arm_A_Letrozole_alone_CRI06.fq.gz
jc5894_er_letrozole_40mg_megestrol_acetate.bed.gz
jc5895_er_letrozole_40mg_megestrol_acetate.bed.gz
jc5896_er_letrozole_160mg_megestrol_acetate.bed.gz
jc5897_er_letrozole_160mg_megestrol_acetate.bed.gz
jc5898_er_letrozole_alone.bed.gz
jc5899_er_letrozole_alone.bed.gz
jc5996_er_letrozole_160mg_megestrol_acetate_pre.bed.gz
jc5997_er_letrozole_160mg_megestrol_acetate_post.bed.gz
jc5998_er_letrozole_40mg_megestrol_acetate_pre.bed.gz
jc5999_er_letrozole_40mg_megestrol_acetate_post.bed.gz
jc6000_er_letrozole_alone_pre.bed.gz
jc6001_er_letrozole_alone_post.bed.gz
jc6272_er_letrozole_160mg_megestrol_acetate.bed.gz
jc6273_er_letrozole_160mg_megestrol_acetate.bed.gz
jc6274_er_letrozole_alone.bed.gz
jc6275_er_letrozole_alone.bed.gz
jc6276_er_letrozole_160mg_megestrol_acetate.bed.gz
jc6277_er_letrozole_160mg_megestrol_acetate.bed.gz
jc6520_er_letrozole_only.bed.gz
jc6521_er_letrozole_only.bed.gz
jc6522_er_letrozole_and_megestrol_acetate_160mg.bed.gz
jc6523_er_letrozole_and_megestrol_acetate_160mg.bed.gz
jc6524_er_letrozole_and_megestrol_acetate_40mg.bed.gz
jc6525_er_letrozole_and_megestrol_acetate_40mg.bed.gz

jc6532_er_letrozole_and_megestrol_acetate_40mg.bed.gz
jc6533_er_letrozole_and_megestrol_acetate_40mg.bed.gz
jc6534_er_letrozole_only.bed.gz
jc6535_er_letrozole_only.bed.gz
jc6536_er_letrozole_and_megestrol_acetate_160mg.bed.gz
jc6537_er_letrozole_and_megestrol_acetate_160mg.bed.gz
jc6582_er_letrozole_and_megestrol_acetate_40mg.bed.gz
jc6583_er_letrozole_and_megestrol_acetate_40mg.bed.gz
jc6584_er_letrozole_and_megestrol_acetate_160mg.bed.gz
jc6585_er_letrozole_and_megestrol_acetate_160mg.bed.gz
jc6586_er_letrozole_and_megestrol_acetate_40mg.bed.gz
jc6587_er_letrozole_and_megestrol_acetate_40mg.bed.gz
jc6588_er_letrozole_and_megestrol_acetate_40mg.bed.gz
jc6589_er_letrozole_and_megestrol_acetate_40mg.bed.gz
jc6669_er_letrozole_and_megestrol_acetate_160mg.bed.gz
jc6670_er_letrozole_and_megestrol_acetate_160mg.bed.gz
jc6671_er_letrozole_alone.bed.gz
jc6672_er_letrozole_alone.bed.gz
jc6673_er_letrozole_and_megestrol_acetate_160mg.bed.gz
jc6674_er_letrozole_and_megestrol_acetate_160mg.bed.gz
jc6675_er_letrozole_and_megestrol_acetate_40mg.bed.gz
jc6676_er_letrozole_and_megestrol_acetate_40mg.bed.gz
jc6677_er_letrozole_and_megestrol_acetate_160mg.bed.gz
jc6678_er_letrozole_and_megestrol_acetate_160mg.bed.gz
jc6679_er_letrozole_and_megestrol_acetate_40mg.bed.gz
jc6680_er_letrozole_and_megestrol_acetate_40mg.bed.gz
jc6681_er_letrozole_alone.bed.gz
jc6682_er_letrozole_alone.bed.gz
jc6683_er_letrozole_and_megestrol_acetate_40mg.bed.gz
jc6684_er_letrozole_and_megestrol_acetate_40mg.bed.gz
jc6685_er_letrozole_alone.bed.gz
jc6686_er_letrozole_alone.bed.gz
jc6687_er_letrozole_and_megestrol_acetate_40mg.bed.gz
jc6688_er_letrozole_and_megestrol_acetate_40mg.bed.gz
jc6689_er_letrozole_and_megestrol_acetate_160mg.bed.gz
jc6690_er_letrozole_and_megestrol_acetate_160mg.bed.gz
jc6691_er_letrozole_alone.bed.gz
jc6692_er_letrozole_alone.bed.gz

Genome browser session (e.g. UCSC): No longer applicable

Methodology

Replicates: One sample per patient per trial visit.

Sequencing depth: Illumina HiSeq 4000 was used for the ChIP-seq experiments. 20-30 million reads were aimed for most samples incorporated into the study.

Antibodies: A 50:50 mix of Millipore: 06-935, Abcam: ab3575, Lot numbers GR3217431-9 and 3045593

Peak calling parameters: macs2 callpeak -t <chip> -c <input> -f BAM -g hs -n <chip name>-X-<input name> -q 0.05 -m 5 50 --nomodel

Data quality: Peak quality was assessed with an in-house built quality evaluation tool which is an integral part of the analysis pipeline (see deposited code)

Software: Bowtie2 version 2.2.6. MACS2 version 2.1.1.20160309, bedtools v 2.26.0-97, DiffBind v 2.2.12, Meme 4.9.1,5.0.5
